# Future prospects of high-entropy alloys as next-generation industrial electrode materials

**DOI:** 10.1039/d3sc06784j

**Published:** 2024-05-08

**Authors:** Saikat Bolar, Yoshikazu Ito, Takeshi Fujita

**Affiliations:** a School of Science and Engineering, Kochi University of Technology 185 Miyanokuchi, Tosayamada Kami City Kochi 782-8502 Japan fujita.takeshi@kochi-tech.ac.jp; b Institute of Applied Physics, Graduate School of Pure and Applied Sciences, University of Tsukuba Tsukuba 305-8573 Japan

## Abstract

The rapid advancement of electrochemical processes in industrial applications has increased the demand for high-performance electrode materials. High-entropy alloys (HEAs), a class of multicomponent alloys with unique properties, have emerged as potential electrode materials owing to their enhanced catalytic activity, superior stability, and tunable electronic structures. This review explores contemporary developments in HEA-based electrode materials for industrial applications and identifies their advantages and challenges as compared to conventional commercial electrode materials in industrial aspects. The importance of tuning the composition, crystal structure, different phase formations, thermodynamic and kinetic parameters, and surface morphology of HEAs and their derivatives to achieve the predicted electrochemical performance is emphasized in this review. Synthetic procedures for producing potential HEA electrode materials are outlined, and theoretical discussions provide a roadmap for recognizing the ideal electrode materials for specific electrochemical processes in an industrial setting. A comprehensive discussion and analysis of various electrochemical processes (HER, OER, ORR, CO_2_RR, MOR, AOR, and NRR) and electrochemical applications (batteries, supercapacitors, *etc.*) is included to appraise the potential ability of HEAs as an electrode material in the near future. Overall, the design and development of HEAs offer a promising pathway for advancing industrial electrode materials with improved performance, selectivity, and stability, potentially paving the way for the next generation of electrochemical technology.

## Abstract 2

High-entropy alloys hold significant promise as electrode materials, even from industrial aspect. This potential arises from their ability to optimize electronic structures and reaction sites, stemming from their complex and adjustable composition.

## Graphical abstract

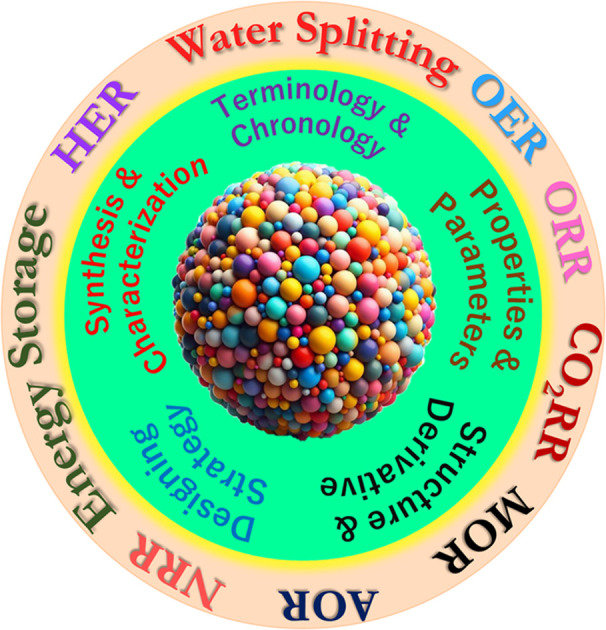

## Introduction

The development of industrial electrode materials to meet the requirements of modern civilization, which is dependent on the industry, is a pressing issue, and research in this field has been rapidly escalating in recent years.^1^ With the continuous development of industrial methods, electrochemical technology was inevitably applied to various industrial processes more than a century ago.^2,3^ More recently, industrial revolutions such as the development of the hydrogen energy sector, battery industry, and fuel cell technology have relied heavily on electrochemical processes. The extensive application of electrochemical processes is considered to be the most convenient way to achieve industrial achievements and benefits for the human society.^4^ The advancement of electrochemical processes and their associated electrode materials is essential for realizing state-of-the-art benefits, particularly in industrial applications where the selection of electrode materials significantly impacts performance outcomes.^5–9^ The design and development of electrocatalysts (electrodes for technical applications) are founded on innovative concepts such as controlled surface roughness, topographic profiles of atoms, catalytically defined active sites, modified electronic structures, atomic rearrangements, and phase transitions during electrochemical reaction processes.^4,6–8^

**Fig. 1:**
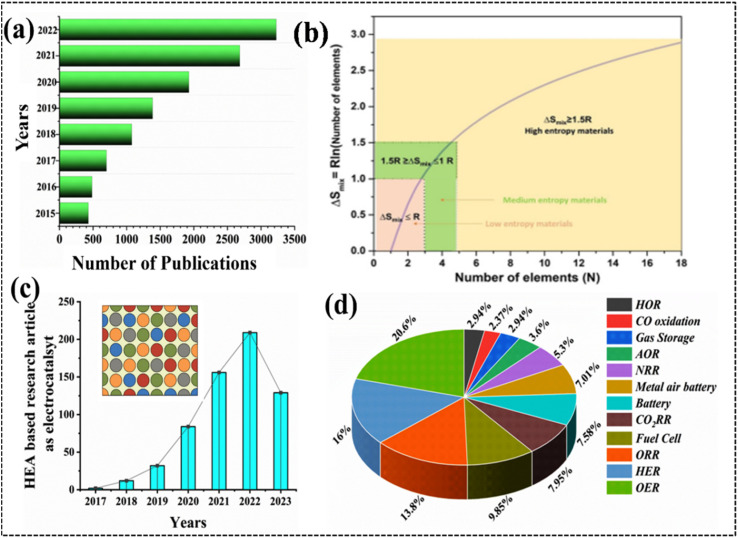
(a) Number of publications on high-entropy alloys (HEAs) according to the data collected from Scifinder Scholar, (b) mixing entropy of equimolar alloys as a function of the number of the components, reproduced with permission.^27^ Copyright 2021, Wiley-VCH. (c) Research articles on HEA (inset)-based electrocatalysts. Reproduced with permission.^29^ Copyright 2016, Elsevier Ltd. (d) HEA electrode materials for electrochemical application.

The advancement of novel electrode materials for industrial applications is a dynamic field of research; consequently, scientists are continuously striving to create electrode materials with higher efficiency, selectivity, stability, and cost effectiveness to meet the demands of industrial processes.^10^ Electrochemical research is immensely significant for transforming electrochemical technology into practical applications and driving transformative advancements across various industries. Electrocatalysts and electrode materials serve as significant components of industrial applications, encompassing energy storage, energy conversion, chemical synthesis, sensors, corrosion protection, electroplating, water treatment and numerous other areas.^10,11^ The selection of electrode materials for a particular application is guided by a combination of factors, including the required conductivity, desired electrochemical properties, and economic considerations. Consequently, as the demand for electrochemical technology surges owing to the rapid industrialization of electrochemical processes, the demand for prospective electrode materials is also anticipated to rise (Fig. 1a).^12–19^ The selection of appropriate electrode materials is a crucial aspect of electrochemical processes, guided by the mechanistic pathways of electrochemical conversion and following the principles of modern electrocatalysis.^14–18^ Precious metals, especially Pt, Ru, Ir, and Au, are frequently employed in electrochemical processes because of their favorable electronic structure, abundance of active sites, high conductivity, selective catalytic activity, and exceptional durability under the respective electrochemical conditions.^20^ However, these properties may vary depending on the specific application within the respective electrochemical process. Despite their advantages, noble metals also exhibit certain limitations in both industrial and lab-scale applications.^21–23^ Their relative scarcity and expense hinder their widespread adoption in large-scale applications. Moreover, they may poison certain contaminants that appear during the electrochemical process. Researchers are actively exploring alternative catalyst materials and designing strategies to reduce the reliance on noble metals while maintaining or improving catalytic performance.^22–24^

Over the past few decades, researchers have endeavored to reduce the price-to-performance ratio of electrocatalysts to enhance their commercial viability and applicability in various industrial processes. This goal can be achieved through two primary approaches: (a) minimizing the utilization of noble metals in the catalyst formulation and (b) developing noble-metal-free electrocatalysts. Noble-metal electrocatalysts often exhibit operational instability under harsh working conditions, resulting in susceptibility to dissolution, agglomeration, and poisoning effects.^10,16,20^ To date, only a limited number of electrocatalysts have been proposed that meet the stringent requirements for industrial applications. Many traditional electrode materials struggle with stability and cost, demanding the exploration of alternatives. Alloy-based materials offer a promising solution, providing a cost-effective and often more stable alternative to expensive noble metal-based electrodes for various electrochemical processes like the OER, HER, and ORR. Recent research efforts have focused on exploring cost-effective alloy-based electrode materials to address the diverse design requirements of various electrochemical processes, considering the limited selection of available metals.^24^ Conventional alloys typically consist of one or two elements, and they often fail in obtaining the specific properties required for an effective electrocatalyst.^25^ With advancements in alloy preparation techniques, a variety of alloy materials have emerged, including low-, medium-, and high-entropy alloys (HEAs) (Fig. 1b).^26–29^ To date, thousands of research papers have been published annually highlighting the promising potential of HEAs in various fields, including electrochemical industrial applications (Fig. 1c).^30–36^

**Fig. 2:**
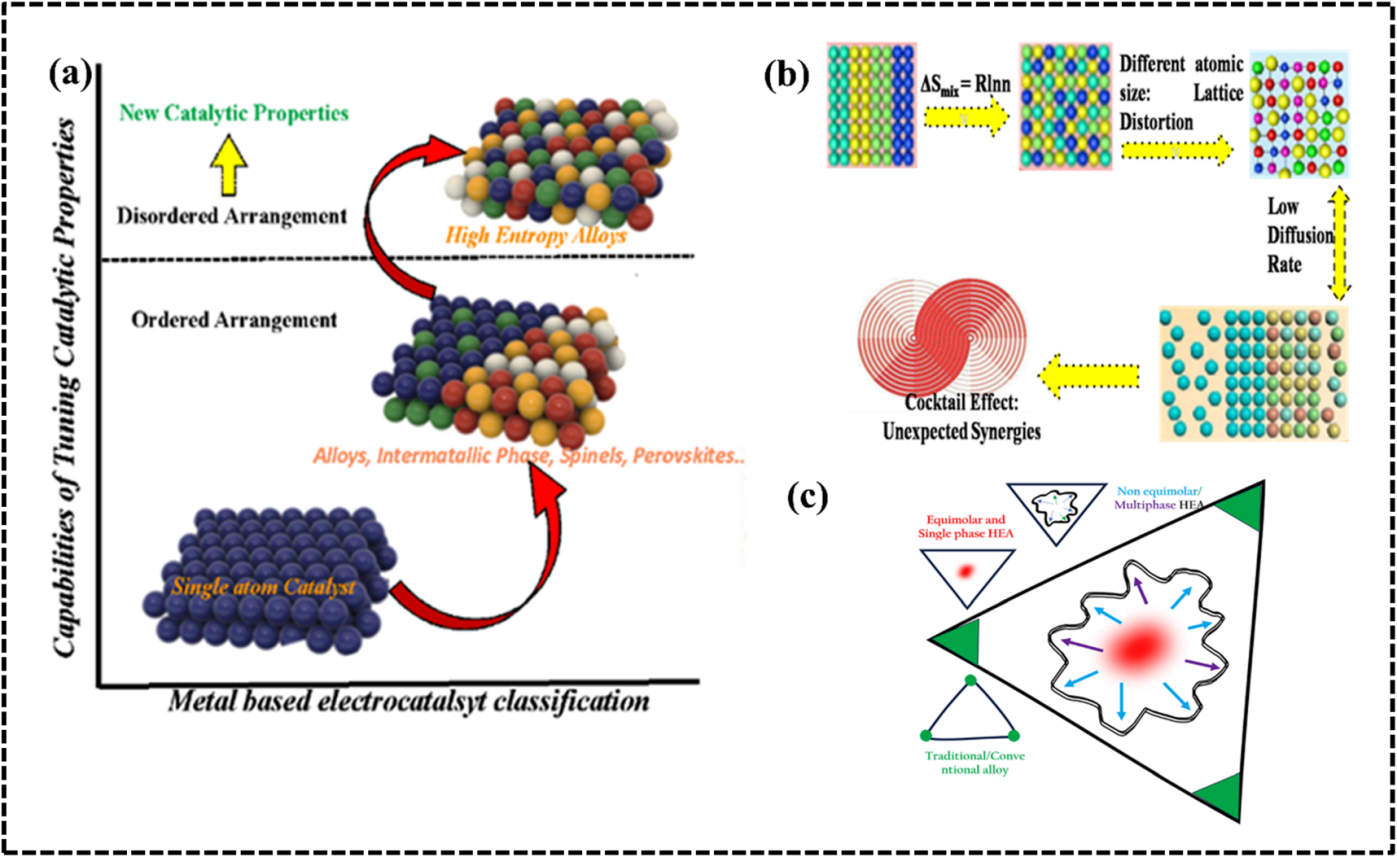
(a) Structural and electronic modification to improve the catalytic performance of HEAs. Reproduced with permission.^40^ Copyright 2021, Wiley-VCH. (b) HEAs with core effects. Reproduced with permission.^5^ Copyright 2016, Elsevier Ltd. (c) Topographies of different alloys and their phase structure.

A HEA consists of at least five or more elements with equal or unequal proportions with atomic percentages in the range of 5–35% of each element (Fig. 1d).^30–34^ Owing to their atomic randomness and superior chemical and physical properties in comparison with those of conventional alloys, HEAs are more appealing for industrial applications. They exhibit several important properties such as the high-entropy effect (HEE), lattice distortion, slow diffusion, and the cocktail effect (CE), which provide considerable versatility and uniqueness compared to other developed materials (Fig. 2a and b).^27,30,31^ The selection of elements and their relative proportions in HEAs are crucial factors for determining their properties and performance. Owing to their wide range of possible compositions, HEAs can be tailored with specific properties, such as phase formation, stability, and processing conditions.^19,22,26,33^ HEAs offer several advantages compared to conventional alloys, including enhanced operational efficiency, productivity, and sustainability.^31–37^ The study of high-entropy alloys (HEAs) in materials science and engineering is exciting due to their unique characteristics and potential applications, driven by the fundamental principle of maximizing configurational entropy within the alloy system to yield desirable properties, including enhanced mechanical properties like strength, hardness, and wear resistance crucial for industrial electrode material design.^33–36^ The versatility of HEAs is one of the key advantages for multiple electrode designs. HEAs can be tailored to exhibit specific properties suitable for a wide range of applications by carefully selecting the constituent elements. This tunability allows for the development of HEAs with tailored electronic and structural features, enabling their application in various electrochemical processes through selective choice of periodic elements, structural features, and synthetic routes. Harnessing their unique properties, HEAs show promise for revolutionizing industrial processes by unlocking exceptional combinations of efficiency, selectivity, stability, and cost-effectiveness in electrochemical applications.

This review delves into the terminology, governing parameters, properties, derivatives, synthesis procedures, structural behavior, and applications of HEA-based materials in diverse electrochemical processes. The discussion extends to theoretical considerations for designing electrode materials tailored to specific applications, providing a comprehensive overview of the potential applications, opportunities, and limitations of HEAs as promising industrial electrode materials.

It further elaborates on the current advancements and potential challenges associated with HEA-based electrode materials from an industrial perspective. The field of HEAs has witnessed continuous expansion, with researchers exploring novel alloy systems, developing advanced processing techniques, and investigating a broad spectrum of properties and applications. The evolving nature of HEAs demonstrates immense promise for further breakthroughs and discoveries in the years to come. The exploration of HEAs remains an active area of research, with scientists continuously pursuing the development of new alloy systems with enhanced properties.

## Terminology and chronology of the development of high-entropy alloys (HEAs)

Conventional alloys are generally referred to as binary, tri- or multi-component alloys depending on the number of metals they comprise; a HEA is a new class of alloy characterized by high constitutive entropy and, which more importantly, does not conform to conventional alloy properties or nomenclature. Compared to other metallic electrode materials (monoatomic catalysts, alloys, intermetallic compounds, spinels, and perovskites), HEAs demonstrate superior catalytic efficiency due to the disordered arrangement of atoms, which gives them new catalytic properties (Fig. 2a). The term HEA has originated from the large magnitude of mixing entropy (*S*_mix_) that is measured during alloying or mixing in a multicomponent system with equiatomic or near-equiatomic proportion (Fig. 2b). In 2004, two individual scientists (J.-W. Yeh and B. Cantor) coined the term HEA for the alloy obtained by randomly mixing elements with high configurational entropy.^27,30,31,38^ In general, there are two definitions of high entropy, one based on configurational entropy and the other on compositional change. It was subsequently proposed that alloys that form solid solutions without intermetallic phases should be considered true high-entropy alloys, since the formation of ordered phases decreases the entropy of the system. However, from a thermodynamic point of view, the two are closely related, since the configurational entropy of the HEA system varies with composition. From a compositional point of view, a HEA is defined as a system containing at least five major elements, each of which is present in the alloy from 5 to 35 atomic (at.) percent (%) of the alloy.^31,38,39^ However, HEAs need not be defined in such a rigid manner as several HEA systems have been developed with one or more elements having concentrations of <5 at%; moreover, even the single solid solution phase concept is not compulsory herein. This elementary definition can be expressed as follows:*n*_major_ ≥ 5, 5 at% ≤ *C*_*i*_ ≤ 35 at%, and *n*_minor_ ≥ 0 *C*_*j*_ ≤ 5,where *n*_major_ and *n*_minor_ denote the number of primary and secondary elements, respectively, and *C*_*i*_ and C_*j*_ denote the atomic percentages of the primary and secondary elements, respectively. An alternative definition of a HEA focuses on the configurational entropy with a ground value of 1.5*R* (previously, it was considered to be 1.6*R* for an equimolar alloy before refinement) in a random state irrespective of whether they are single or multiphase at room temperature (298 K).^40^ Theoretically, HEAs are considered as high-entropy class for materials with Δ*S* ≥ 1.5*R*, and alloys with 1.0*R* ≤ Δ*S* ≤ 1.5*R* and Δ*S* ≤ 1.5*R* are classified as medium- and low-entropy classes, respectively (Fig. 1b).^31–38^ The entropy of the system increases with an increase in the level of disorderness. According to the statistical principle of Boltzmann thermodynamics, the entropy of a system can be expressed as:

1 *S* = *K*_B_ ln *W*

where *K*_B_ is the Boltzmann constant (1.38 × 10^−23^ J K^−1^) related to the molar gas constant *R* (*R* = 8.314 J K^−1^ mol^−1^) and *W* is the thermodynamic probability, which represents the total number of microscopic states contained in the macroscopic state.^37–42^ The total mixing entropy includes configurational entropy (Δ*S*^conf^_mix_), vibrational entropy (Δ*S*^vib^_mix_), magnetic dipole entropy (Δ*S*^mag^_mix_), and electronic randomness entropy (Δ*S*^elec^_mix_) and the relationship is represented as

2 Δ*S*_mix_ = Δ*S*^conf^_mix_ + Δ*S*^vib^_mix_ + Δ*S*^mag^_mix_ + Δ*S*^elec^_mix_

The mixing entropy is completely dependent on the configurational entropy. The configurational entropy of equimolar alloys increases with the increase in the number of elements in the HEA. In the context of the relationship between entropy and complexion, Boltzmann theory formulates the configurational entropy for liquid and solid solutions of equimolar alloys using the following equation:^27,39^3
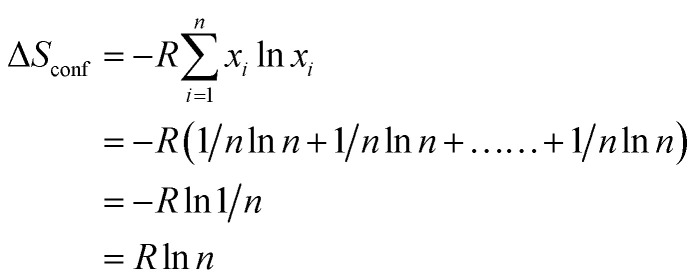
where *R* is the ideal gas constant and *x*_*i*_ represents the molar fraction of the *i*th component. For a given number of components (*n*), the configurational entropy reaches the largest value when the atomic fraction of all components is the same (*i.e.*, equimolar) (Table 1).^27,31,37,39^ Interestingly, the Gibbs–Helmholtz equation provides the relation between enthalpy of mixing (Δ*H*_mix_), Gibbs free energy (Δ*G*_mix_), and entropy of mixing (Δ*S*_mix_). Therefore, as the value of *T*Δ*S*_mix_ increases to more than that of Δ*H*_mix_, the entropy stabilization of the crystal structure is established; therefore, the stability of the compound increases with higher configurational entropy because of the more negative Δ*G*_mix_. HEAs cannot be strictly distinguished by their phases (intermetallic/multiple/single phase).^39,40^

**Table 1:** Ideal configurational entropies in terms of *R* for equiatomic alloys with constituent elements^23^

*N*	1	2	3	4	5	6	7	8	9	10	11	12	13
Δ*S*_mix_	0	0.69*R*	1.1*R*	1.39*R*	1.61*R*	1.79*R*	1.95*R*	2.08*R*	2.2*R*	2.3*R*	2.4*R*	2.49*R*	2.57*R*

In particular, HEAs with a single phase can be distinguished as compositionally complex solid solutions (CCSSs), which are not fully covered by the aforementioned definition of a HEA. All HEAs are CCSSs, but not all CCSSs are HEAs, since HEAs are a special subset of CCSSs, with the main difference being the emphasis on having multiple main elements in approximately equal molar or higher concentrations. In contrast, complex concentrated alloys, multi-principal element alloys (MPEAs), compositionally complex alloys (CCAs), and multi-element alloys are all subsets of CCSSs and differ in elemental integrity, elemental homogeneity, phase structure, lattice structure, and solid solution behavior.^40–44^ The use of these terms in the context of electrocatalysts is often based on the assumption that the properties of both materials coexist. As a result, a HEA can be used as an established electrode material and its electrochemical performance can be considered for use as an industrial electrode material. The molar concentration (equimolar/quasi-equimolar) and phase structure (single or multi-phase) are two important parameters that determine the type of HEA, and based on these parameters, HEAs can be divided into first and second generation HEAs.^27,29,39,44^ Conversely, non-equimolar complex phase (matrix containing solid solution) alloys have attracted potential research attention due to their structural, compositional, and electronic diversity, which may affect their electrochemical performance compared to conventional alloys.

First-generation HEAs consist of at least five components with equal atomic ratios, and the phase structure is a single-phase solid solution. Second-generation HEAs consist of at least four main components with unequal atomic ratios and have a two- or multiphase structure.^45,46^ HEAs are a developing research area, and efforts are being focused on developing non-equimolar multiphase solid solution alloys from equimolar single-phase solid solution alloys in order to improve catalytic performance. Non-equimolar multiphase solid solution alloys are referred to as second generation HEAs and are being analyzed to design and develop more selective materials for electrochemical applications (Fig. 2c).^45,46^

Studies have identified that transition metals generally form single face-centered cubic (FCC) arrangements in a typical solid solution.^40,43,46^ The number of phases in a HEA is always less than the maximum equilibrium number based on the Gibbs phase rule, offering a new perspective on the strategies used for their synthesis. Yeh *et al.* determined that incorporating more elements generally increases the hardness of an alloy and investigated the corrosion inhibition behavior of HEAs, identifying that small passive metals and a lower mixing enthalpy contribute to corrosion resistance.^23,27,31,39,43^

**Fig. 3:**
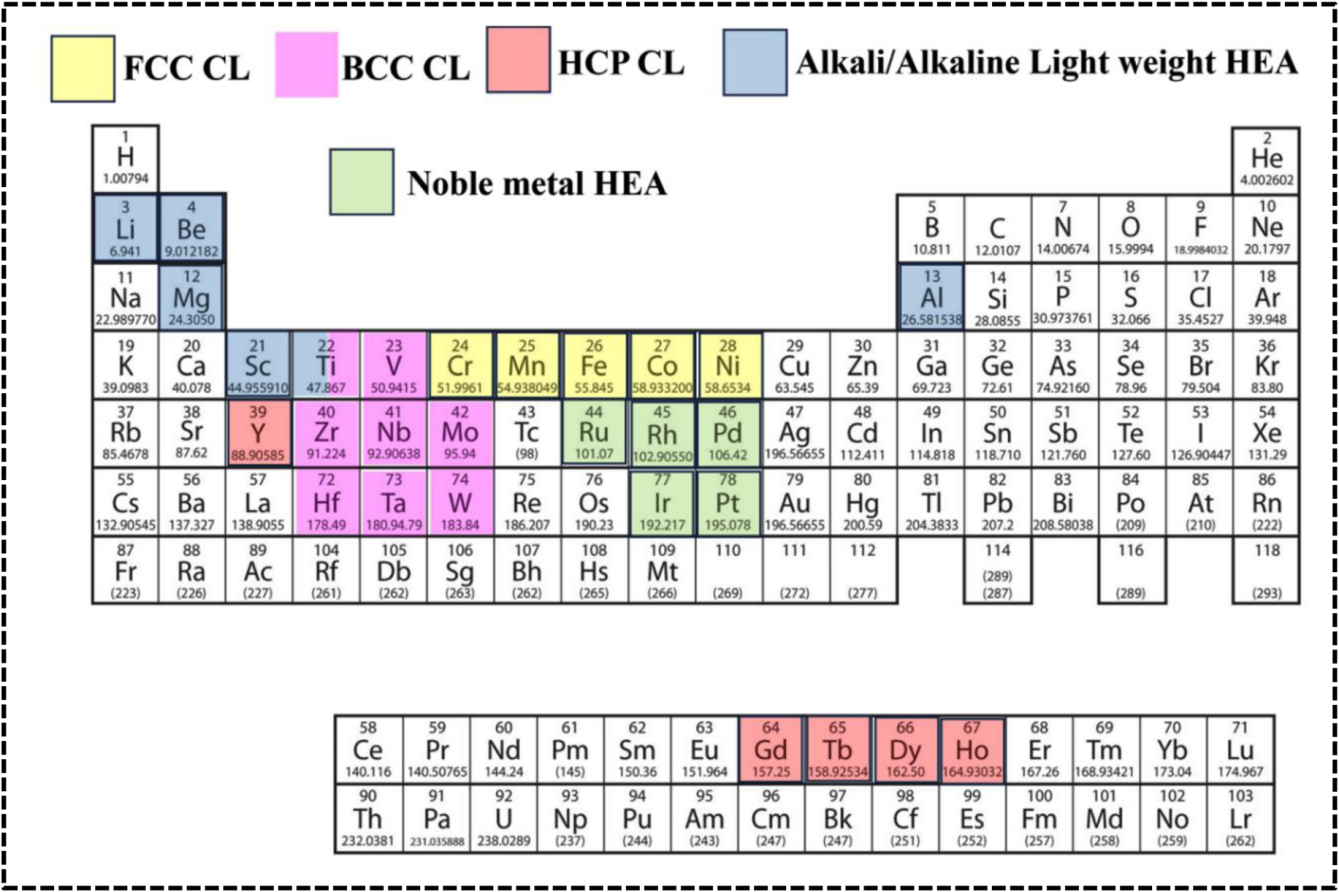
Periodic positions of constituent elements in HEA crystal structures.

The scientific understanding of HEAs has been broadly extended, employed, and accepted to various high-entropy materials (HEMs). In brief, HEA systems broadly encompass high-entropy solid solution alloys, high-entropy amorphous alloys, and narrowly defined HEA ceramics. High-entropy ceramics (HECs) are a specific class of ceramics that exhibit the principles of HEAs but in a ceramic context, where ceramics are solid solutions comprising five or more cation and anion sublattices with high configurational entropy.^47,48^ A variety of HEMs have been synthesized, including metal–organic frameworks (MOFs), carbonaceous materials, MXenes, oxides, chalcogenides, nitrides, phosphides, borides, and fluorides. However, high-entropy derivatives do not completely satisfy the parameters of a HEA system and should be defined as HEMs.^49,50^ The HEAs can be both amorphous and crystalline in nature, similar to conventional alloys. Therefore, the common crystal structures of HEAs and their derivatives can include FCC, body-centered cubic (BCC), hexagonal close-packed (HCP), and amorphous structures; therefore, the structural types are also varied by altering the synthesis process and, most notably, based on the periodic position of the elements (Fig. 3a).^27,29,39,46,51^ Recently, certain mixed-phase crystal structures have been reported with BCC and FCC lattices in the single phase.^51,52^ Elemental selection strategically influences the crystal structure, affecting multiple electrochemical processes diversely, with recent attention drawn to high-entropy alloy nanoparticles (HEA NPs) due to their superior activities and stability, offering opportunities for innovations in various aspects alongside HEAs and their derivatives, whereby HEA NPs with tunable electronic features, optimized morphology, surface-active sites, selective crystal structure, phase integrity, and comparable synthesis techniques significantly impact catalytic objectives.^33^

Depending on the periodic position of the elements, HEAs can be distinguished as noble-metal-based HEAs (NHEAs), non-noble-metal-based HEAs (NMHEAs), and refractory HEAs (RHEAs).^39,47,49^ In contrast, RHEAs are composed of refractory metals (Mo, Ti, V, Nb, Hf, Ta, W, and Cr) exhibiting significant application prospects owing to their superior mechanical properties and their potential in replacing Ni-based superalloys as the next generation of high-temperature electrode materials.^53^ Phase structure modification of HEAs has evolved as an effective approach to increase the strength of the RHEA systems.^39,40,52,54^ Eutectic HEAs (EHEAs) typically have FCC and BCC crystal lattice structures and exhibit two different properties, such as ductility and strength. Sumanta *et al.* reported an equiatomic CoCuFeNiTi EHEA with BCC and FCC structures, which exhibited optimum workability at high temperature and can be used in high temperature electrochemical applications such as high-temperature solid oxide electrolyzer cells (SOECs).^55^ However, no study reporting the application of EHEA- and high-entropy superalloy-based electrode materials in the electrochemical process has been published.

## Thermodynamic features and kinetic properties and parameters of HEAs

The multi-element nature of HEAs and their derivatives results in significant effects compared to conventional bi- and tri-metallic alloys, leading to superior kinetic and thermodynamic properties in HEAs compared to other metallic materials. The thermodynamic and kinetic parameters are directly or indirectly interrelated and must be analyzed to understand the overall electrochemical performance of HEAs. Four core effects such as the (a) HEE, (b) lattice distortion effect (LDE), (c) sluggish diffusion effect (SDE), and (d) CE are exhibited as a result of the thermodynamic and kinetic properties of HEA-based materials (Fig. 2b and d).^27,36,40,46^ The introduction of equimolar, near-equimolar, or non-equimolar components increases the coordination entropy (eqn (3) and (4)) and stabilizes the single-phase structure at elevated temperatures. In HEAs, atoms of different sizes randomly occupy lattice sites, which causes a large lattice distortion. This distortion slows the diffusion of atoms, causing a kinetic effect called slow diffusion. Slow diffusion can lead to nanoprecipitation and amorphous phase formation during HEA growth. Finally, the CE is caused by the presence of multiple elements with different properties. Interestingly, thermodynamic parameters such as the HEE can affect the HEA matrix and cause other core effects which can be distinguished based on kinetic parameters.

### High-entropy effect

The stability of multi-metallic materials can be represented with the help of the Gibbs free energy equation: Δ*G*_mix_ = Δ*H*_mix_ − *T*Δ*S*_mix_, where *G*, *H*, *T*, and *S* represent the Gibbs free energy, enthalpy, temperature, and entropy, respectively. The entropy of metals is higher in the melted state than in the solid state, and the difference is approximately equal to the gas constant *R* according to Richard's law.^56,57^ Therefore, the formation of high-entropy solid solution alloys is favored when the mixing entropy of equimolar-ratio alloys sufficiently surpasses the mixing enthalpy, leading to increased coordination and random distribution of alloy atoms in lattice positions, thereby minimizing atomic ordering and segregation.^55–58^

**Fig. 4:**
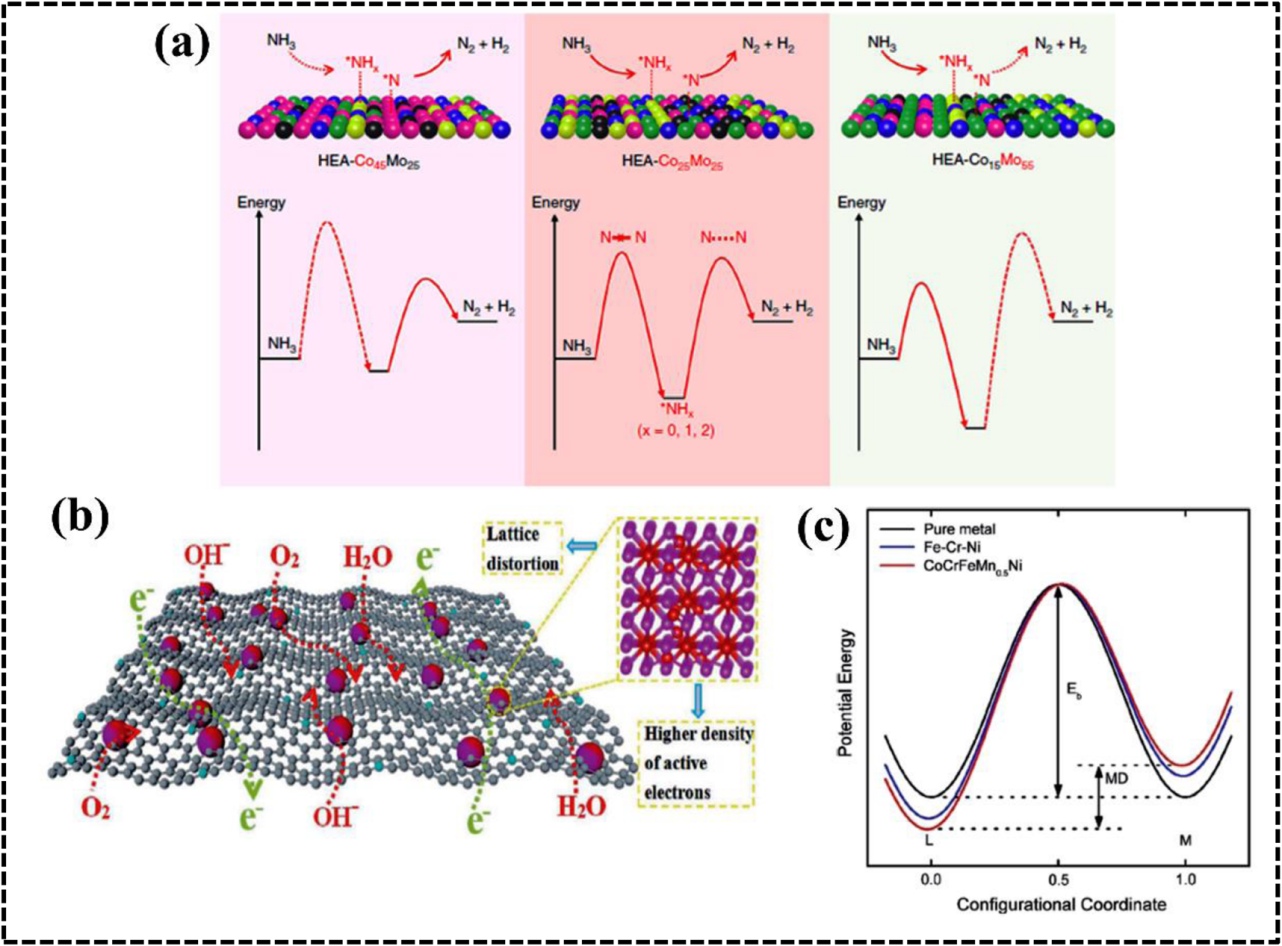
(a) Schematic illustration of the rate-limiting factors in NH_3_ decomposition. Reproduced with permission.^64^ Copyright 2019, Springer Nature. (b) Schematic diagram of the variation of lattice potential energy and mean difference during the migration of a Ni atom in different matrices. Reproduced with permission.^65^ Copyright 2013, Elsevier Ltd. (c) Schematic exhibition of the advantages of Fe-enriched FeNi_3_ intermetallic nitrogen-doped carbon for bifunctional oxygen electrocatalysts. Reproduced with permission.^69^ Copyright 2021, Elsevier Ltd.

As configurational entropy increases in HEAs, Gibbs free energy decreases, enhancing system stability and promoting compatibility, favoring solid solution formation over intermetallic compounds, thereby improving thermodynamic stability.^29,58–60^ Moreover, the increase in configurational entropy significantly reduces the mixing kinetic energy barrier of constituent elements in HEAs, enabling adjustment and optimization of electronic and crystal structures for the development of HEA-based materials as potential electrocatalysts.^61–63^ For instance, random surface mixing in CoMoFeNiCu-HEA nanostructures, with uniform distribution of Co and Mo sites, facilitates the dehydrogenation of NH_3_ molecules and desorption of N_2_ from the HEA surface in catalytic decomposition reactions of NH_3_ (Fig. 4a).^64^

### Lattice distortion effect

This effect arises from the creation of a high-entropy solid solution involving multiple principal elements, leading to lattice distortion due to the random and disordered orientation of these elements within the same lattice matrix during HEA system growth. In brief, in a solid solution lattice structure of a multicomponent alloy, each lattice point is surrounded by others with different atomic sizes, shapes, electronic features, and coordination environments; this typically results in a lattice mismatch, leading to the distortion of the lattice structure.^27,39,64,65^ The LDE creates strain and stress over the crystal lattice leading to an increase in the overall free energy and influence over the electrical and thermal conductivities of the system. Furthermore, the apparent lattice mismatch leading to lattice distortion implies that HEAs exist in a thermodynamically nonequilibrium state, which can reduce the energy barrier for adsorption, activation, and conversion of molecules in electrocatalytic reactions.^63–66^ Moreover, strain and stress engineering serve as established methods for enhancing electrochemical performance by reducing activation energy requirements for active intermediates.^67,68^ Consequently, HEA-based materials can be tailored to explore strain and stress engineering, yielding improved electrode materials for specific electrochemical processes.^27,39,40,68^ The lattice distortion in an Fe-enriched alloy promoted a higher density of active electrons around the Fermi level, resulting in faster electronic mobility at the electrode–electrolyte interface (Fig. 4c).^69^

### Sluggish diffusion effect

The growth of HEAs and their derivatives is impacted by the SDE, resulting in slower phase transformation rates compared to that of conventional alloys, stemming from distinct neighboring arrangements and bonding characteristics of constituent atoms, wherein the diffusion phenomenon relies on the energy of lattice sites for jumping atoms.^29,39,68^ The diffusion rate varies with the periodic position of the elements, affecting the jumping ability of the atoms. Notably, a higher diffusion rate results in a higher jumping possibility and *vice versa*.^27–29,39^ The distinctive sluggish diffusion kinetics of high-entropy alloys (HEAs), primarily attributed to the HEE, account for their superior electrochemical properties compared to conventional alloys, as evidenced by experimental studies demonstrating lower diffusion coefficients and higher activation energies in HEAs relative to those of constituent metals.^68–70^ The sluggish diffusion in HEAs is explained by the fact that larger lattice potential energy fluctuations produce more significant atomic traps and blocks, leading to higher activation energies.^71^ The slow diffusion in HEAs can increase the energy barrier for variations in atomic diffusion, which facilitate their application as electrocatalysts by preserving the relative stability of single-phase structures.

### Cocktail effect

The properties of HEA materials include those of all the constituent elements rather than those of only an individual element.^40^ This comprehensive performance due to the synergistic effect results in unexpected properties that can optimize the catalytic performance of HEAs by reducing reaction energy barriers and enhancing reaction kinetics. This fascinating property is termed as the CE and was introduced by Ranganathan *et al.* in 2003.^47^ The composite effect is observed owing to atomic-to micro-scale multiphase conversion of the constituent elements. Notably, the CE involves strong interactions within the elements due to the formation of single/multiphase solid solutions with thermodynamically acceptable low Gibbs free energy. The interactions of the elements in a HEA modify the electronic and structural properties, resulting in an improved catalytic performance.^72^

The different elemental compositions redistribute the d-band structure of HEAs and modified density of states (DOS) altering the electronic structure and enhanced active surface sites, which was reflected in their alcohol oxidation efficiency.^73,74^ Moreover, chemical interaction and charge-transfer kinetics of HEA systems are governed by the developed CE, which can be described by using a potential energy diagram (Fig. 4b).^65^ Interestingly, a transition metal plays a vital role in creating synergistic interaction, thereby improving the electrochemical performance. Therefore, the CE in HEAs demonstrates significant impact on the electrochemical processes, particularly when incorporating selective transition metals with different d-orbital electronic configurations.^71^ However, physical consequences of this outcome remain unclear and require further experimental and theoretical investigation of the electronic and structural features to design a potential electrode material.

Thermodynamic features such as mixing enthalpy, configurational entropy, phase diagram representation, and solid solution stability are crucial parameters to characterize high-entropy alloys (HEAs), providing insights into their stability, phase formation, and properties, facilitating tailored designs for specific applications. Similarly, understanding the kinetic properties of HEAs, encompassing phase transformations, diffusion, and microstructural evolution, is essential for optimizing processing conditions, predicting material behavior, and designing HEAs with the desired microstructures and properties. These combined thermodynamic and kinetic effects serve as a foundation for utilizing HEAs as industrial electrode materials, although additional factors must also be considered in their design.

## Rational design of HEAs with industrial application prospects

To rationally design an efficient electrocatalyst, comprehensive theoretical and experimental knowledge is required.^5,32,40^ In this respect, several research studies have attempted to investigate the effect of other parameters to define more reliable thermodynamic criteria for phase selection of multicomponent HEAs. Most of the studies are based on statistical analyses of a large database of synthesized HEAs with their reported phase or phases and have focused on factors distinguishing the conditions required for solid solution phase formation from that in intermetallic compounds. Therefore, several studies have attempted to understand the influence of other parameters on defining more reliable thermodynamic criteria for phase selection in HEAs rather than in intermetallic materials. These studies indicate the necessity for other effective deign parameters to achieve a stable single phase and efficient industrial electrode materials.

### Design parameters and principle

The selection of the appropriate elements in HEAs and their compositional involvement can help tune catalytic performance. In the case of fully stable solid solution HEAs, tuning certain parameters can influence the design of the HEA and its application in certain electrochemical processes.

#### Enthalpy and entropy of mixing (Δ*H*_mix_/Δ*S*_mix_)

**Fig. 5:**
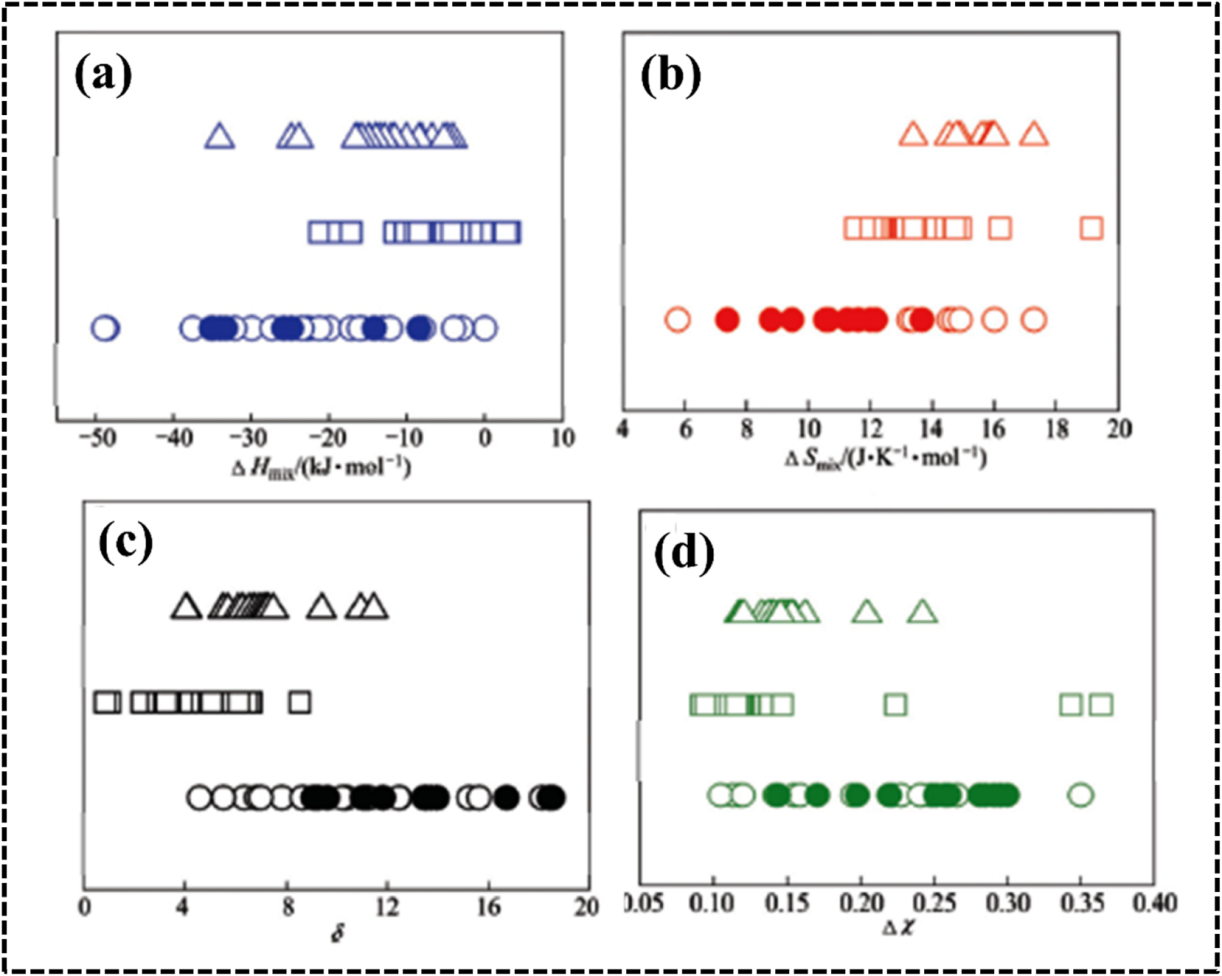
(a–d) Effect of Δ*H*_mix_, Δ*S*_mix_, *δ*, and Δ*χ* on phase stability in HEAs. The symbol ○ represents equiatomic amorphous phase forming alloys; ● represents non-equiatomic amorphous phase forming alloys; □ represents solid solution phases and △ represents intermetallic phases. Reproduced with permission^78^ Copyright 2011, Elsevier Ltd.

The Gibbs–Helmholtz equation of free energy defines the stability of a HEA based on experimental values at a constant temperature. The model proposed by Miedema can be used to determine the Δ*H*_mix_ of a multicomponent alloy system, as shown below:^75,76^4
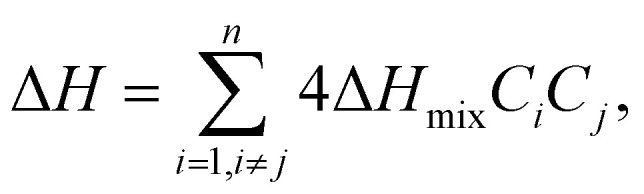
where *n* denotes the number of components, *C*_*i*_ and *C*_*j*_ are the concentrations of components *i* and *j*, respectively, and Δ*H* (*Ω*_*ij*_ = 4*H*_mix_), a regular solution interaction parameter, is the mixing enthalpy of the two components. Gibbs free energy describes the conditions under which a solid solution or amorphous phase is formed during HEA growth. Solid solutions are formed when the mixing enthalpy is highly or moderately positively loaded with negative Gibbs free energy values. Conversely, the amorphous phase forms when the mixing enthalpy is fully loaded with negative Gibbs free energy values (Fig. 5a and b).^78^ The specified range of Δ*H*_mix_ for solid solution phase formation indicates that as the absolute value of mixing enthalpy decreases, a regular solution approaches the ideal case.^77,78^ Therefore, the smaller the Δ*H*_mix_, the more favorable the formation of a single-phase solid solution. Conversely, as the binding energies of the constituents increase, Δ*H*_mix_ becomes negative, and as Δ*H*_mix_ becomes positive, the miscibility gap becomes smaller, indicating that the constituents in solution are randomly distributed.

#### Atomic size (*δ*)

**Fig. 6:**
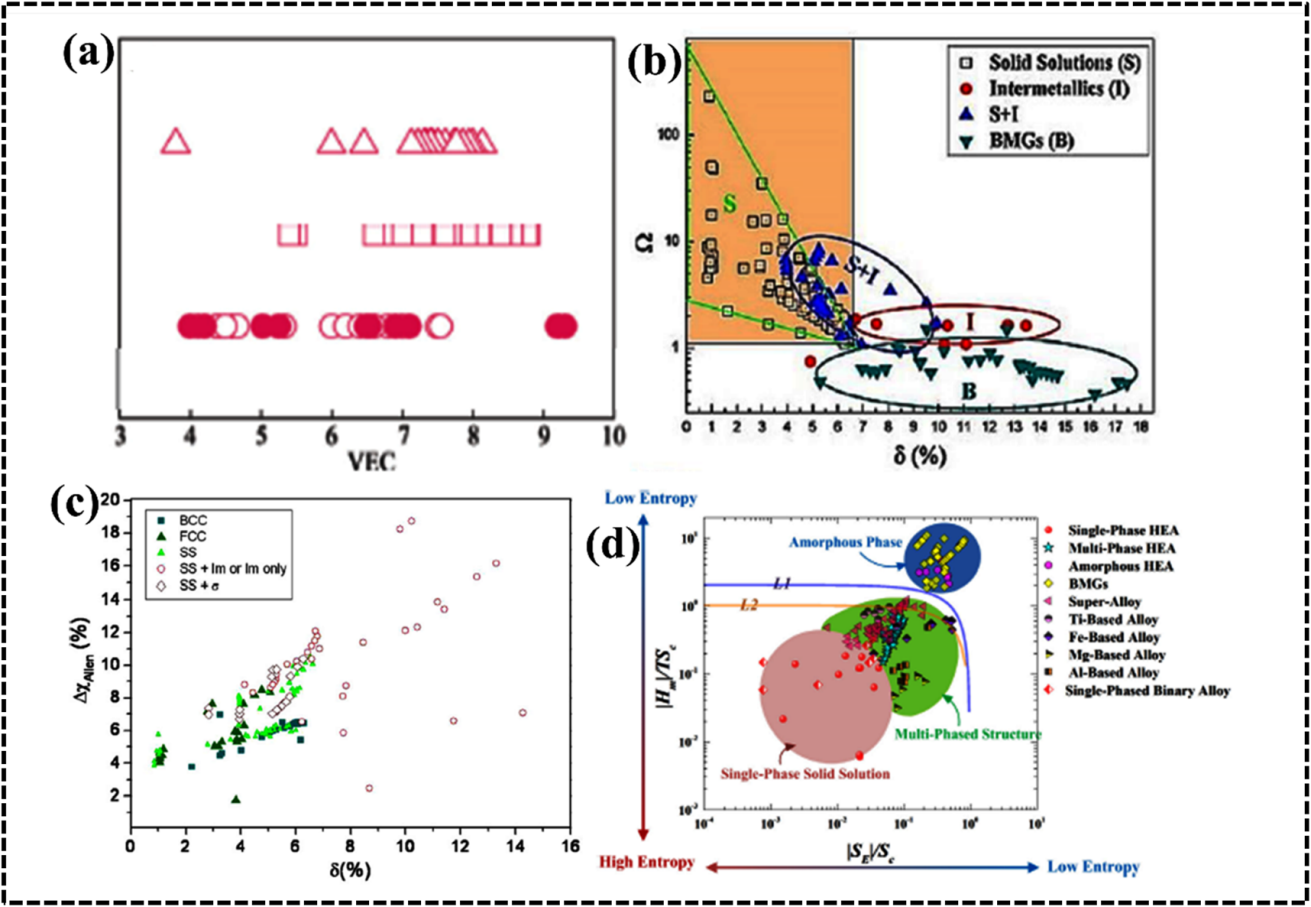
(a) Effect of valence electron concentration (VEC) on phase stability in HEAs. The symbol ○ represents equiatomic amorphous phase forming alloys; ● represents non-equiatomic amorphous phase forming alloys; □ represents solid solution phases and △ represents intermetallic phases. Reproduced with permission.^78^ Copyright 2011, Elsevier Ltd. (b) Relationship between parameters. Reproduced with permission.^88^ Copyright 2012, Elsevier Ltd. (c) Radius *versus* electronegativity mismatch for HEAs. Reproduced with permission.^82^ Copyright 2014, Elsevier Ltd. (d) Thermodynamic phase diagram of HEAs. Reproduced with permission.^89^ Copyright 2015, Elsevier Ltd.

The difference in atomic size between the constituent elements can influence the formation of single-phase solid solutions, affecting crystal structure homogeneity, which can have an impact on thermal and electrical conductivity. Elements with similar atomic sizes have higher probability of occupying neighboring lattice sites, whereas lattice mismatches occur when the atomic sizes of the elements vary significantly.^75–77^ Therefore, the atomic size difference can be used to explain the phase stability of multicomponent systems using the following equation:^75,78^5
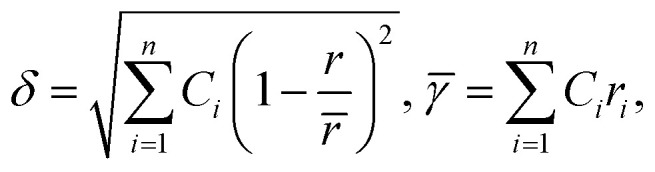
where *n* denotes the number of components, *C*_*i*_ and *r*_*i*_ denote the concentration and atomic radius of the *i*th component, respectively, and *r* is the average atomic size of the *n* components in the alloy. Guo *et al.* proposed that a lower atomic size difference results in the formation of a solid solution (*δ* ≤ 8.5), whereas an amorphous phase is formed at a comparatively higher atomic size difference (*δ* ≥ 9) (Fig. 5c).^78–80^ However, a few reports have predicted a solid solution phase with a *δ* value of 6.6, where the small *δ* value favors the formation of a disordered solid solution (DSS) (Fig. 6b).^80^ The choice of the elements plays a key role in designing an electrocatalyst with required crystal features.^46^ The catalytic performance can be tuned by using lattice structure modulation, type of crystal structure, and lattice distortion, resulting in an alteration of the phase structure, strain generation, and active crystal plane formation.

#### Electronegativity (*χ*)

Electronegativity of the constituent elements has an impact on the phase of the designed HEAs and their derivatives. The electronegativity difference between a mixture of elements is determined by using the following equation:^77–80^6
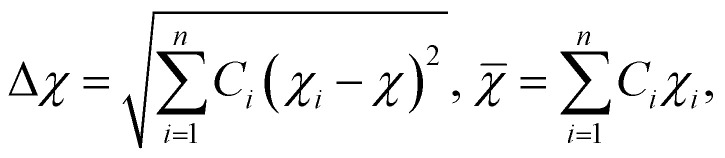
where *n* denotes the number of components, *C*_*i*_ and *χ*_*i*_ are the concentration and Pauling electronegativity of the *i*th component, respectively, and *χ* is the average electronegativity of the *n* components in the alloy. However, Allen proposed a new definition of electronegativity and the measured electronegativity difference (Δ*χ*) is represented as follows:7
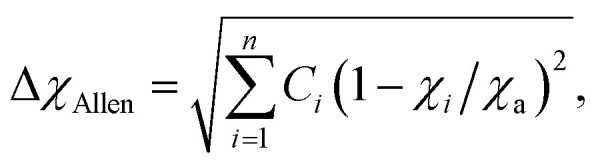
where *χ*_*i*_ is the electronegativity of the *i*th element and *χ*_a_ is the average electronegativity of the elements in the alloy.^81^ Interestingly, the radius *versus* electronegativity mismatch plot provides information about multicomponent systems. Thus, the difference in radius *versus* electronegativity suggests that when the radius mismatch is between 1% and 6% and Δ*χ*_Allen_ is between 3 and 6, no intermetallic compounds (including the *r* phase) will be formed and only solid solutions will be formed (Fig. 6c).^82^ Particularly in the region where both the electronegativity and the radius difference are high, the majority of the elements combine to form intermetallic compounds.^46,77^ These findings suggest that the radius and simple parameters such as electronegativity difference are highly indicative but not always decisive for the design of HEA-based electrode materials (Fig. 5d).^78^

#### Valence electron concentration

The total number of valence electrons is determined by using the valence electron concentration (VEC) of a multicomponent system and it is expressed as:8
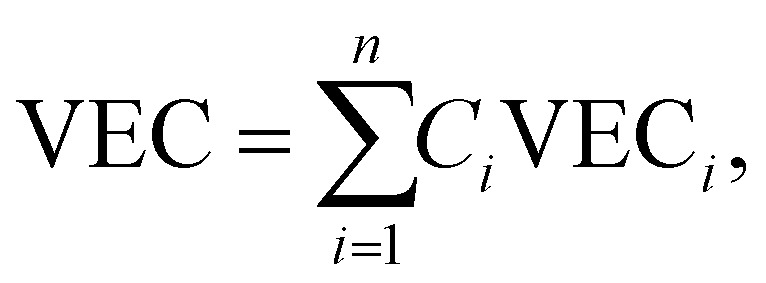
where *n* denotes the number of components and *C*_*i*_ and VEC_*i*_ are the concentration and VEC of the *i*th component, respectively. Unlike *χ* and the radius difference, the VEC parameter does not control the stability of phase formation. According to the Hume-Rothery rule, the VEC of an alloy affects the crystal structure of the solid solution phase in the presence and/or absence of atomic size effects (Fig. 6a).^78,83^ Systematic experimental and theoretical studies have shown that VEC ≥ 8 and VEC < 6.87 favor the formation of FCC and BCC lattice structures, respectively. The valence electron concentration (VEC) can provide valuable insights into tailoring the d-electron concentration of HEAs, which may affect their electrocatalytic activity.^84–86^ By varying the density of states (DOS) and local oxidation states within HEAs, VEC data can serve as a guide for predicting and designing electrode materials based on HEAs. However, it is important to recognize the limitations of VEC as a perfect predictive tool, as complex interactions and oxidation states of individual elements may deviate from their estimated values.^87^

#### Omega (*Ω*) parameter

The prediction and stability of the HEA solid solution phase is mathematically determined by using the *Ω* parameter. Mathematically, the *Ω* parameter is represented as the ratio of entropic contribution to Gibbs free energy at high temperature to enthalpy input, as shown below:^88^9
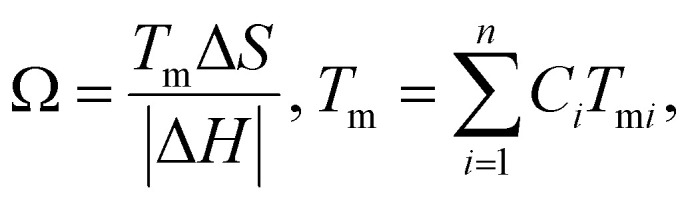
where *n* denotes the number of components, *C*_*i*_ and *T*_m*i*_ are the concentration and melting point of the *i*th component, respectively, and *T*_m_ is the weighted average melting point for a mixture of *n* components. The phase stability and formation possibility of an HEA can be determined from the dimensionless *Ω* parameter that measures the energy gain on mixing of the constituent elements. In this case, as *Ω* increases, the probability of single-phase random solid solution formation in the HEA increases, and *vice versa* (Fig. 6b).^76,88^

#### Excess entropy (SE)

The numerical value of HEAs can be maximized by maintaining an equal molar fraction of the elements. Therefore, configurational entropy of HEAs can be increased by increasing the total number of elements in an equiatomic composition. However, in a non-ideal or real solution, the real entropy is determined by using the Boltzmann equation and the total entropy of mixing (*S*_T_) can be represented by using *S*_T_ = *S*_C_ + *S*_E_, where *S*_E_ and *S*_c_ are the excess and actual configurational entropies, respectively. Notably, all types of entropies are functions of the concentration (*C*_*i*_), atomic radius (*r*_*i*_), and packing fraction (*ξ*) of the elements.^88^ Mathematical derivation of the above-mentioned equation provides an inequality condition with two different dimensionless thermodynamic parameters, which are represented as follows:^89^10
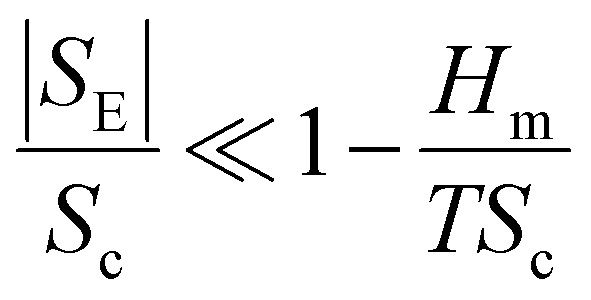


This inequality condition includes a criterion for single-phase solid solution formation. Higher excess entropy and mixed enthalpy are observed in multicomponent systems containing amorphous phases, and their formation is almost favorable for obtaining the optimum enthalpy (Fig. 6d).^77,89^ This result suggests that a lower ratio of these two terms favors the formation of single phase solid solutions where entropy is dominant, and a higher ratio favors the formation of multiphase solid solutions and intermetallic compounds.^76^ Higher excess entropy and mixed enthalpy were observed in the multicomponent HEA systems containing amorphous phases, and their formation favors a combination of enthalpy and entropy.

#### *Φ* parameter

To design HEAs with multiple phase structures according to catalyst requirements, a dimensionless parameter is defined based on excess and constitutive entropies, assuming *T* = *T*_m_ and max {*H*_*i*_} ∼ Δ*H*_mix_, and it is formulated as:11
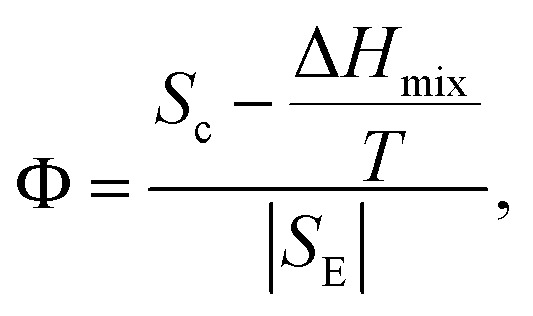


**Fig. 7:**
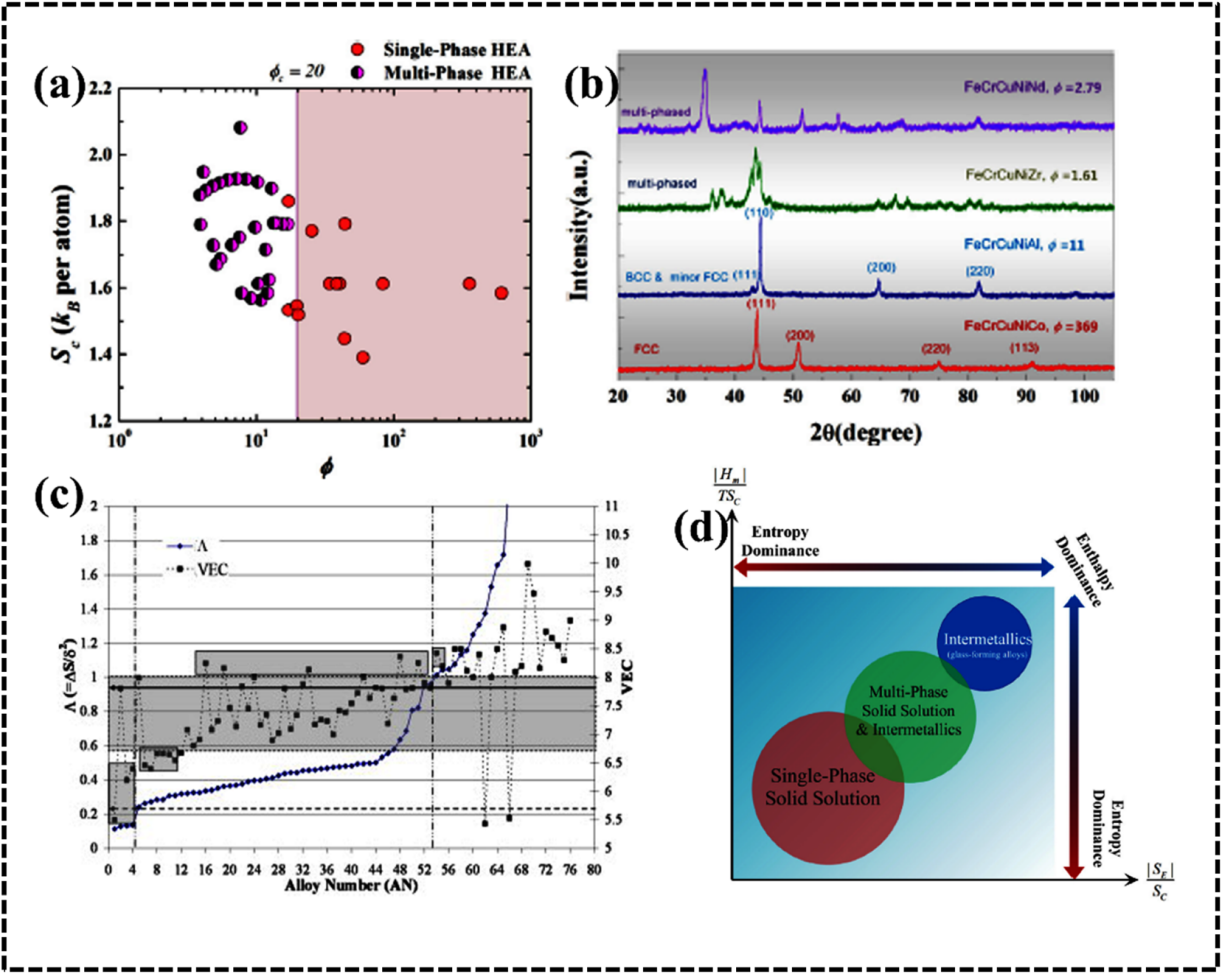
(a) Plot of Sc *versus ϕ* for a variety of HEAs with either single- or multiphase structures and (b) X-ray diffraction (XRD) pattern with the *ϕ* parameter. Reproduced with permission.^90^ Copyright 2015, Elsevier Ltd. (c) Plot of *Λ* (=Δ*S*_mix_/*δ*^2^) and Δ*H*_mix_ (secondary *y*-axis) with the alloy number. Reproduced with permission.^86^ Copyright 2014, Elsevier Ltd. (d) Illustrated generalized thermodynamic phase diagram for equilibrium phases. Reproduced with permission.^89^ Copyright 2015, Elsevier Ltd.

HEAs are grouped differently according to their corresponding *Φ* values. The different HEAs are separated by a critical value of *Φ*_c_ = 20. Clearly, HEAs exhibit a single-phase solid solution when *Φ* > *Φ*_c_ and a multiphase, even amorphous, structure when *Φ* < *Φ*_c_ (Fig. 7a).^90^ This behavior suggests that *Φ* is a promising descriptor for ranking HEAs based on their tendency to form single-phase solid solutions (Fig. 7b).^90^

#### Geometric parameter *Λ*

*Λ* is a geometric parameter that provides information about the configuration of the lattice and radius of atoms. This new parameter is defined as follows:^76,86^12
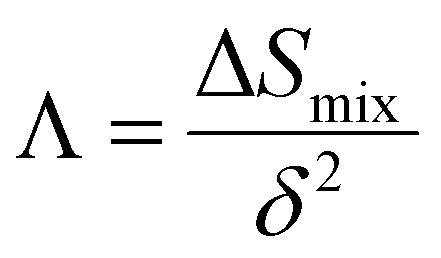


The formation of the DSS for HEAs can be understood by considering the *Λ* parameter and comparing it with the other available parameters. Interestingly, the possibility of DSS is favored by an increased Δ*S*_mix_ and decreased *δ* value; consequently, this geometric parameter is useful for identifying the condition of single- (*Λ* > 0.96), two- (0.24 < *Λ* < 0.96), and multiphase (*Λ* < 0.24) HEAs.^77,90^ A recent report suggested that the *δ* parameter can effectively predict the formation of different types of phases when compared to previously discussed parameters such as Δ*H*_mix_, VEC, *Φ*, *δ*, and Δ*χ* parameters (Fig. 7c).^86^

#### Effect of kinetic factors on phase formation

The process of phase formation in high-entropy alloys (HEAs) is mainly governed by thermodynamic parameters, and kinetic factors also play a crucial role, especially in the formation of intermetallic phases from an initial single solid solution phase.^91^ Thus, while thermodynamic equilibrium determines phase stability, kinetic processes such as cooling, solidification, annealing, or calcination also affect phase stability and formation, which can have a significant impact on the growth, stability, and product percentage of high-entropy alloys. Intermetallic phase formation was observed to decrease in CoCrFeNiTi_0.4_ HEAs with higher cooling rates and faster kinetics, leading to the formation of a solid-state kinetically stable single-phase product.^92^ The perturbation model proposed by Luan *et al.* demonstrates that, as the number of elements increases, the potential for the formation of intermetallic compounds increases, destabilizing single-phase solid solutions and promoting the formation of multiphase structures.^93^ This is because, while an increase in the number of elements provides an entropic advantage for single-phase solid solution formation, it also introduces an unfavorable contribution of enthalpy to the total Gibbs free energy associated with mixing. This highlights the crucial role of high temperature in the phase stability of HEAs. High temperature reduces the Gibbs free energy, increasing the stability of the single-phase solid solution (Fig. 7d). As a result, based on thermodynamic factors alone, most single-phase HEAs that are stable at high temperatures become unstable at room temperature, resulting in a heterogeneous structure.^77^ However, the dynamic effects of kinetic factors during synthesis, such as rapid or slow cooling of multi-component systems, can have a significant impact on the resulting phase formation. The parameters described are essential for the design and development of the required HEA-based electrocatalysts; Hume-Rothery proposed the following as factors determining the stability of the alloy phase:

(a) an atomic radius ratio of less than 15%;

(b) similar crystal structures of the constituent elements;

(c) comparable valency for complete dissolution;

(d) low electronegativity difference to avoid the formation of intermetallic compounds.

The formation of metal solid solutions is primarily governed by two crucial factors: radius mismatch and electronegativity alternation. These rules play a pivotal role in determining the characteristics of a HEA and its derivatives. However, when these factors alone are insufficient to explain HEA properties, other considerations come into play. These additional rules are consulted to fine-tune and optimize HEAs through adjustments in synthesis processes and precursor elements. The strategic design of HEAs for industrial electrode materials is paramount, necessitating meticulous experimental exploration and the judicious selection of elements. Hence, HEA design principles are centered on maximizing configurational entropy, introducing complex atomic arrangements, exploiting atomic size mismatch, and careful selection of the constituent elements to achieve the desired properties and ensure stability.

### Derivatives of HEAs

Research and application of HEA-based materials are on the rise, with a focus on enhancing catalytic performance in electrochemical processes by optimizing electronic environments and solid solution microstructures. HEA derivatives, including HECs, as well as composite or hybrid structures utilizing stable templates such as carbonaceous compounds, MOFs, MXenes, and 2D materials, are being actively explored. HECs encompass a wide range of materials, including high-entropy oxides (HEOs), nitrides (HENs), carbides (HECbs), borides (HEBs), hydrides, silicides (HESis), sulfides (HESs), fluorides (HEFs), phosphides (HEPs), phosphates (HEPO_4_s), oxynitrides (HEONs), carbonitrides (HECNs), and boro-carbonitrides (HEBCNs).^49^ Interestingly, unlike HEAs, all HEMs exhibit long-range structural order but remain compositionally disordered. Hence, the advent of HECs presents a plethora of opportunities for property tuning and overcoming material application bottlenecks. On one hand, in stark contrast to the straightforward FCC, BCC, and HCP structures of metals, ceramics boast a broader array of crystal structures, facilitating the design of HEC-based electrocatalysts tailored for commercial applications.^94–96^

#### High-entropy metal–organic frameworks

**Fig. 8:**
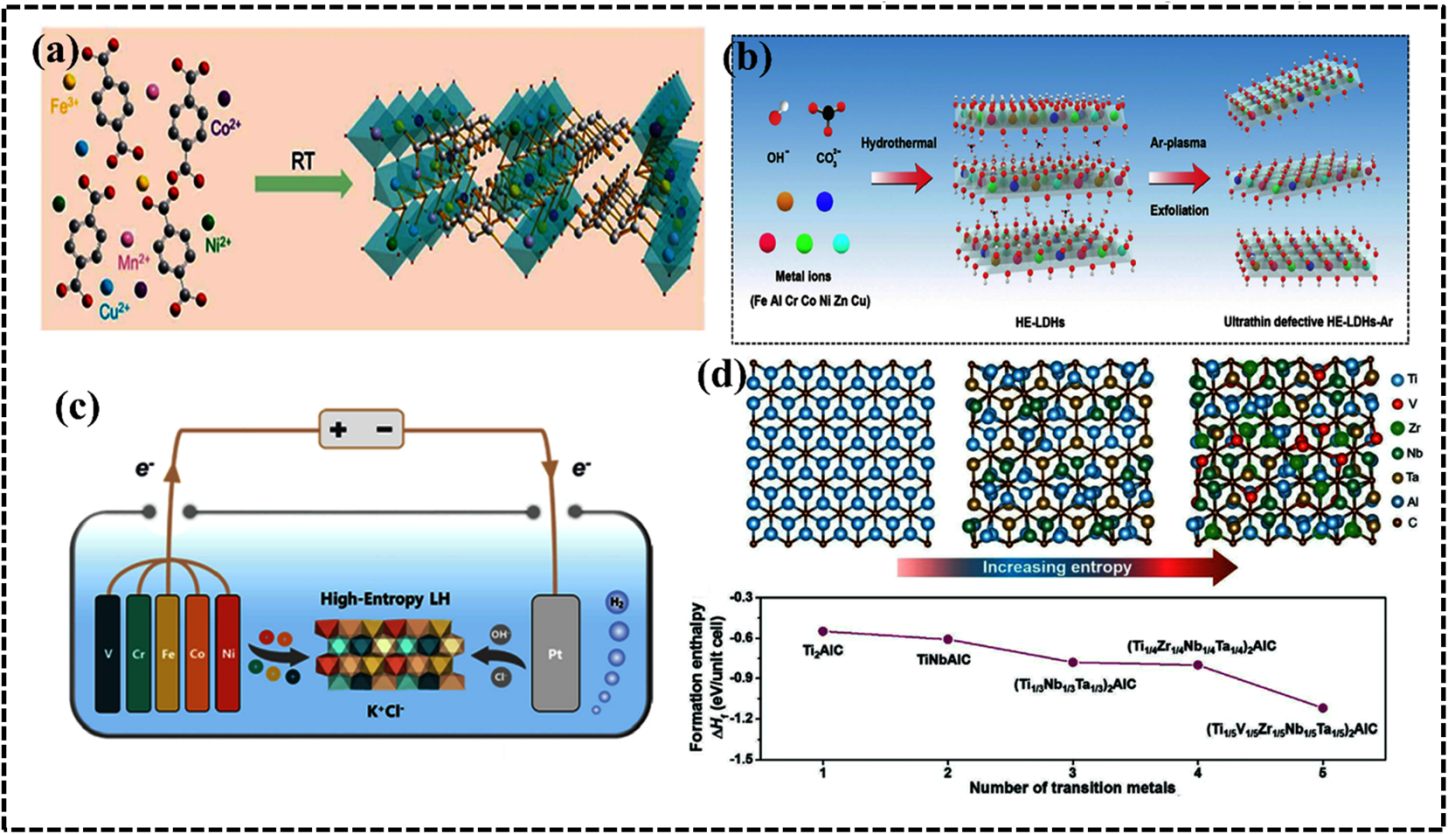
(a) Synthesis procedure for the preparation of HE-MOF-RT under ambient conditions. Reproduced with permission.^98^ Copyright 2019, Royal Society of Chemistry. (b) Synthesis and structural characterization of high-entropy layered double hydroxides. Reproduced with permission.^109^ Copyright 2021, Elsevier Ltd. (c) Schematic illustration of the electrolytic synthesis of HEAs. Reproduced with permission.^112^ Copyright 2022, Elsevier Ltd. (d) Diagram of lattice distortions and formation enthalpy calculated using density functional theory (DFT) for five MAX phases with Ti, V, Zr, Nb, and Ta species. Reproduced with permission.^116^ Copyright 2021, Wiley-VCH.

Compared to conventional MOFs, high-entropy MOFs (HE-MOFs) feature ions of varying sizes, potentially leading to lattice disorder and unique properties. A mechanochemical approach driven by entropy was employed to synthesize HE-MOF architecture, utilizing a zeolitic imidazolate framework (ZIF) and transition metals (Zn^2+^, Co^2+^, Cd^2+^, Ni^2+^, and Cu^2+^). The resulting HEA supported on a ZIF framework (HE-ZIF) demonstrated superior catalytic conversion of CO_2_ to carbonate compared to single-metal ZIF materials, likely due to the synergistic effect of five metal ions acting as Lewis acids during epoxide activation.^97^ A HE-MOF hybrid demonstrated that the solution phase method is more efficient that the solvothermal technique as it achieves improved mass transfer because of magnetic stirring and homogenous thermal distribution (Fig. 8a).^98^ The homogenous distribution of the metal over the MOF, creating a synergistic effect within the architecture helps to improve the catalytic performance.^98^ The improved catalytic efficiency of MOF-based nanoalloy assemblies (MOF-HEAs), such as (CoNi, FeCo, and CuCoNi), can be attributed to the porous carbon support and high specific surface area.^99–102^ Moreover, the HE-MOF template can be used to synthesize nanosized HEA/C by pyrolysis, which is considered as an important electrode material in harsh electrolyte media. Huang *et al.* used a one-step solvothermal method to first synthesize a HE-MOF nanorod precursor, which was then annealed to obtain MnFeCoNiCu/CHEA nanoparticles (NPs) with a particle size less than 5 nm.^103^ The pyrolysis temperature influences the homogenous distribution of the NPs, which indirectly affects the effective active site of the designed electrocatalyst. The MOF-HEA offers a versatile platform for design, achieved by modifying both the organic backbone and the choice of metals utilized.

#### High-entropy hydrotalcites

High-entropy hydrotalcites (HEHs) are compounds in which the interlayer anions are noncovalently bonded to the positively charged host layer.^104–109^ Ternary or quaternary layered double hydroxides (LDHs) were successfully synthesized by co-precipitation methods as heterogeneous catalysts and were termed high-entropy LDHs (HE-LDHs) or HEHs.^105^ HE-LDHs have demonstrated better catalytic efficiency than the low-entropy LDH owing to the decreased activation energy and developed lattice distortion phenomena in the presence of a multimetallic arrangement.^105,109–111^ The choice of metal ions and their sizes simultaneously affect the structure and electronic environment of the HEH system. In this context, Wang *et al.* synthesized defect-rich ultrathin nanosheet HE-LDH/HEH *via* argon plasma exfoliation (Fig. 8b).^111^ The radius of the metal-ion precursor minimizes lattice mismatch, leading to a solid solution phase that ultimately increases the constitutive entropy of HEHs. Polymetallic sites on the surface provide a nearly continuous distribution of adsorption energy, demonstrating significant potential for further tuning of the position of the volcano plot.^111^ As expected, the ultrathin defective HE-LDH enhanced the activity of the oxygen evolution reaction (OER) in alkaline media, showing lower overpotential and faster reaction rates than those of pure HE-LDH/HEH. A systematic analysis of the composition and structure of the prepared quinary layered (oxy)hydroxide (CoFeNiCrV-LH) nanosheets reveals that they have an optimized defect-rich low-crystalline structure.^112^ These optimized accessible active sites and the defect-rich low-crystalline structure were observed to originate from an entropy-driven lattice structure with high configuration entropy. The optimized accessible active sites effectively promoted charge transfer, mass transport, and the formation of reactive intermediates, and electrochemical data suggest improved performance of the OER catalyst (Fig. 8c). Importantly, the synthetic strategy and choice of metal ions must be carefully considered when designing promising electrode materials for specific electrochemical processes.

#### High-entropy MXenes

The high-entropy MXene (HE-MXene) is introduced from high-entropy MAX phases, also termed M_*n*+1_AX_*n*_ (*n* = 1, 2, 3), where M represents early transition-metal elements, A is an element that is primarily from groups 13–16, and X stands for C and/or N.^113–115^ The mono or bimetallic MAX phase is transformed into the multimetallic MAX phase as the potential precursor element of the HE-MXene. Theoretical investigation suggested that increasing the number of transition-metal components in the MAX phase significantly decreased enthalpy formation per unit cell (Fig. 8d).^116^ In fact, the design and synthesis of the high-entropy MAX phase precursor is a crucial step for successfully preparing thermodynamically and kinetically stable HE-MXenes. The size, electronic structure, and chemical environment of the metal ions collectively influence the overall electronic structure of the HE-MXene system. The concept of entropy stabilization offers a promising strategy to enhance the stability of 2D MXenes under harsh conditions. Notably, HE-MXenes exhibit pronounced lattice distortions, resulting in high mechanical strains within the atomic layer. These high mechanical strains can effectively promote nucleation and uniform growth in battery material designs. Du *et al.* demonstrated that the incorporation of size-compatible transition-metal elements (Ti, V, Zr, Nb, and Ta) can stabilize MXenes ((Ti_1/5_V_1/5_Zr_1/5_Nb_1/5_Ta_1/5_)_2_CT_*x*_) at the atomic level, completely exposing active sites for improved electrochemical energy conversion and storage performance.^117^ The dispersed transition metals significantly enhanced configurational entropy, lowering Gibbs free energy and forming a lattice-distorted single-phase HE-MXene system.^118^

#### High-entropy oxides

**Fig. 9:**
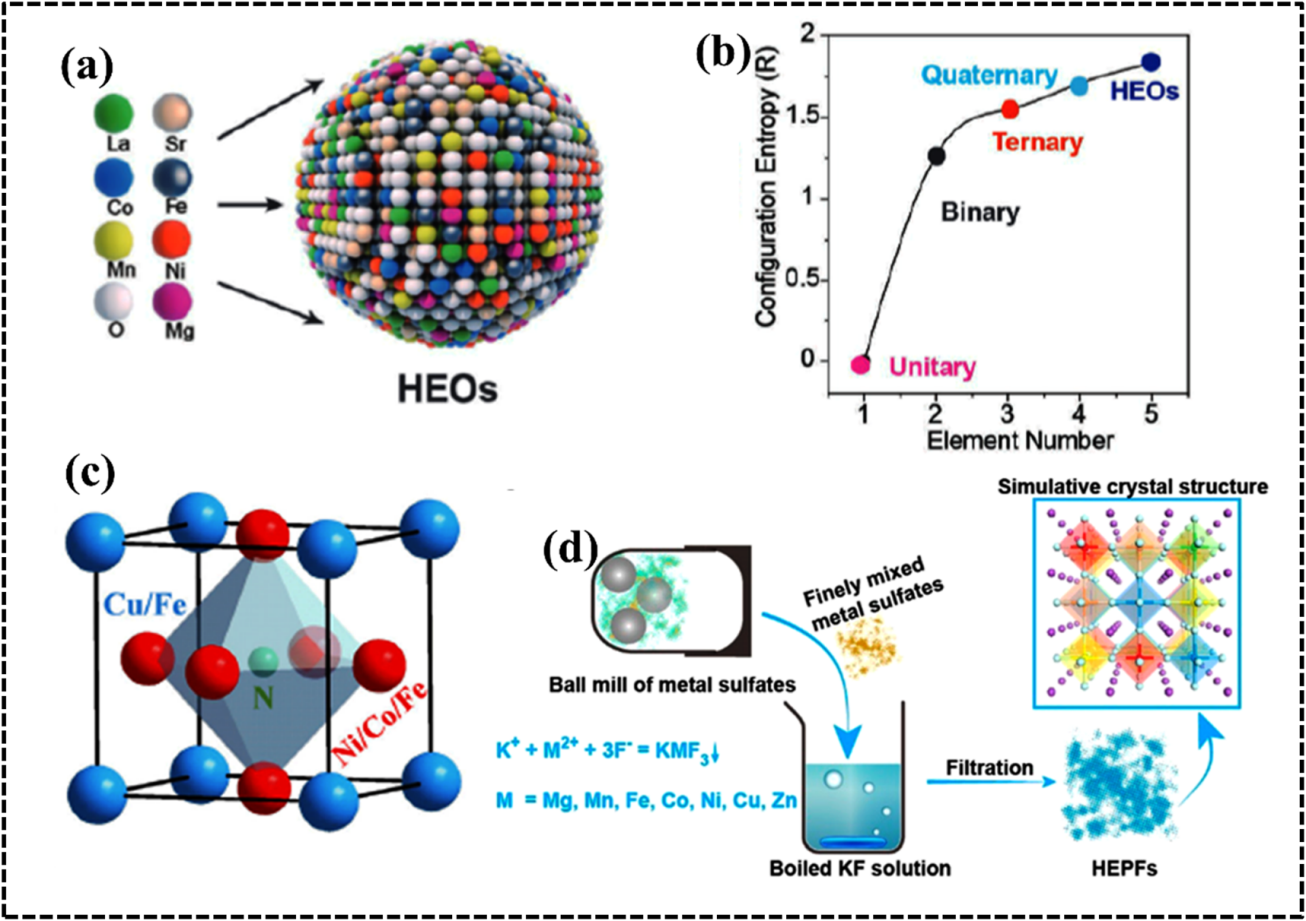
(a) Schematic demonstration of the structure of high-entropy perovskite oxide nanoparticles with uniformly dispersed elements. (b) Configurational entropy of perovskites. Reproduced with permission.^126^ Copyright 2022, Wiley-VCH. (c) Unit crystal structure of anti-perovskite nitride. Reproduced with permission.^144^ Copyright 2023, Elsevier Ltd. (d) Synthesis of high-entropy perovskite fluorides. Reproduced with permission.^145^ Copyright 2020, American Chemical Society.

The synthesis of single-phase HEOs is more challenging than that of HEAs because of the diversity of their crystal structures and local site symmetry. HEOs form stable crystal lattices owing to the arrangement of oxygen atoms; however, they have lower entropy than HEA systems because of the ordered arrangement of oxygen ions as confirmed by single-phase NiMgCuCOZnO with a crystalline rock-salt structure.^119^ Recalling the basic concept of thermodynamics, one would expect the entropy of the entire system to be maximized, since the cations of the metal oxide structure are expected to contribute equally to the lattice. The difference in cationic radii of the individual components should be small enough to form a single-phase HEO. Furthermore, at least one of the cationic crystal structures of the entire system should have inherent electronegativity. Furthermore, the solubility of the components is also considered a selection rule for producing effective HEOs. The geometric and electrical balance factors are important for determining the composition of HEO systems, whereas the coordination number (CN) value and electronegativity have limited impact on the stability and formation possibility of HEOs.^119–122^ Depending on the arrangements of the lattice points, HEOs can acquire various crystal structures such as rock salt, perovskite, spinel, fluorite, and pyrochlore structures. The structural variation, tailored properties, and entropic stabilization of HEOs have attracted significant attention as potential electrode materials in energy storage and conversion applications.^121–136^ Moreover, structural and electronic modification within a specific HEO system can be achieved by synthetic procedures that certainly promote electrochemical performance in different aspects.^125–135^ For instance, Tang *et al.* synthesized a series of high-entropy perovskite cobaltites, namely (La_0.6_Sr_0.4_Co_0.2_Fe_0.2_Mn_0.2_Ni_0.2_Mg_0.2_O_3_), wherein the introduction of structural entropy led to the creation of additional oxygen vacancies within the framework, consequently augmenting the OER performance (Fig. 9a and b).^126^ HEOs have been used in multiple electrochemical applications, such as supercapacitors,^130^ batteries,^128,129,131,136^ and water splitting.^126,132,133^ Recently, rare-earth materials have been used to synthesize HEOs.^134^

#### High-entropy nitrides

High-entropy metal nitrides with exceptional chemical stability in a harsh electrolyte medium and superior physical properties such as hardness, thermal stability, and electrical conductivity, have garnered significant industrial interest.^137,138^ In 2004, Chen *et al.* fabricated nitrides of Fe–Co–Ni–Cr–Cu–Al–Mn and Fe–Co–Ni–Cr–Cu–Al_0.5_ by reactive sputtering and studied their hardness for application as coating materials.^138^ The hardness of HENs can be monitored by using the high N content that directly influences the VEC.^139^ Owing to the two important abilities of corrosion and oxidation resistance, they are considered as potential electrode materials under harsh conditions. Both these properties are due to the presence of a stable oxide layer and creation of a nitrogen-rich layer during the electrochemical process.^140,141^ A mechanochemical-assisted soft urea strategy was successfully employed to synthesize HEN comprising Cr, Nb, Mo, and Zr with a crystalline structure exhibiting potential electrical double layer capacitance for supercapacitor applications.^142^ The superior corrosion and oxidation resistance properties of carbon-supported FeCoNiCuMnN HEAs were further enhanced by the stability gained through the formation of a unique integrated nanowire/nanosheet structure.^143^ Crystal structure modulation is an effective approach for improving the performance of HEN-based electrode materials for energy storage and conversion processes. For instance, Zhu *et al.* designed high-entropy anti-perovskite metal nitrides as highly efficient electrocatalysts for the OER (Fig. 9c).^144^ Notably, high-entropy anti-perovskite nitride materials, in which multiple elements occupy equivalent lattice sites, are considered to have the potential for effective applications in electrocatalysis.^144^

#### High-entropy fluorides

Due to the highly electronegative F atom and the formation of weak M−F bonds, AB_3_F-type perovskite fluorides exhibit high ionic character, making them potential electrode materials for energy conversion.^145^ HEF materials with perovskite structures exhibit enhanced conductivity, leading to improved catalytic performance due to the reduced charge-transfer resistance, minimized mass transfer limitations, and favorable electrode–electrolyte electron transfer processes. The formation of ABF_3_-type high-entropy perovskite fluorides is governed by the hexa-coordinated ionic radius of the B-site cations. To minimize lattice mismatch barriers, transition-metal ions occupying B sites should possess comparable radii. Wang *et al.* selected seven metal ions (Mg, Mn, Fe, Co, Ni, Cu, and Zn) to synthesize perovskite-type HEFs and achieved promising OER catalytic performance.^145^ However, the synthesis strategy has a pronounced influence on the formation of single-phase high configurational entropy of the HEF systems (Fig. 9d).^145^ The inherent structural defects and distorted lattices in HEFs are pivotal for enhancing OER activity. The intricate interactions among diverse elements within a single-phase solid solution contribute to the distinctive catalytic activity of HEFs. In comparison to single-metal catalysts, HEFs offer a synergistic platform featuring highly dispersed active sites, abundant fluorine sites, and efficient diffusion channels.^146^

#### High-entropy sulfides

**Fig. 10:**
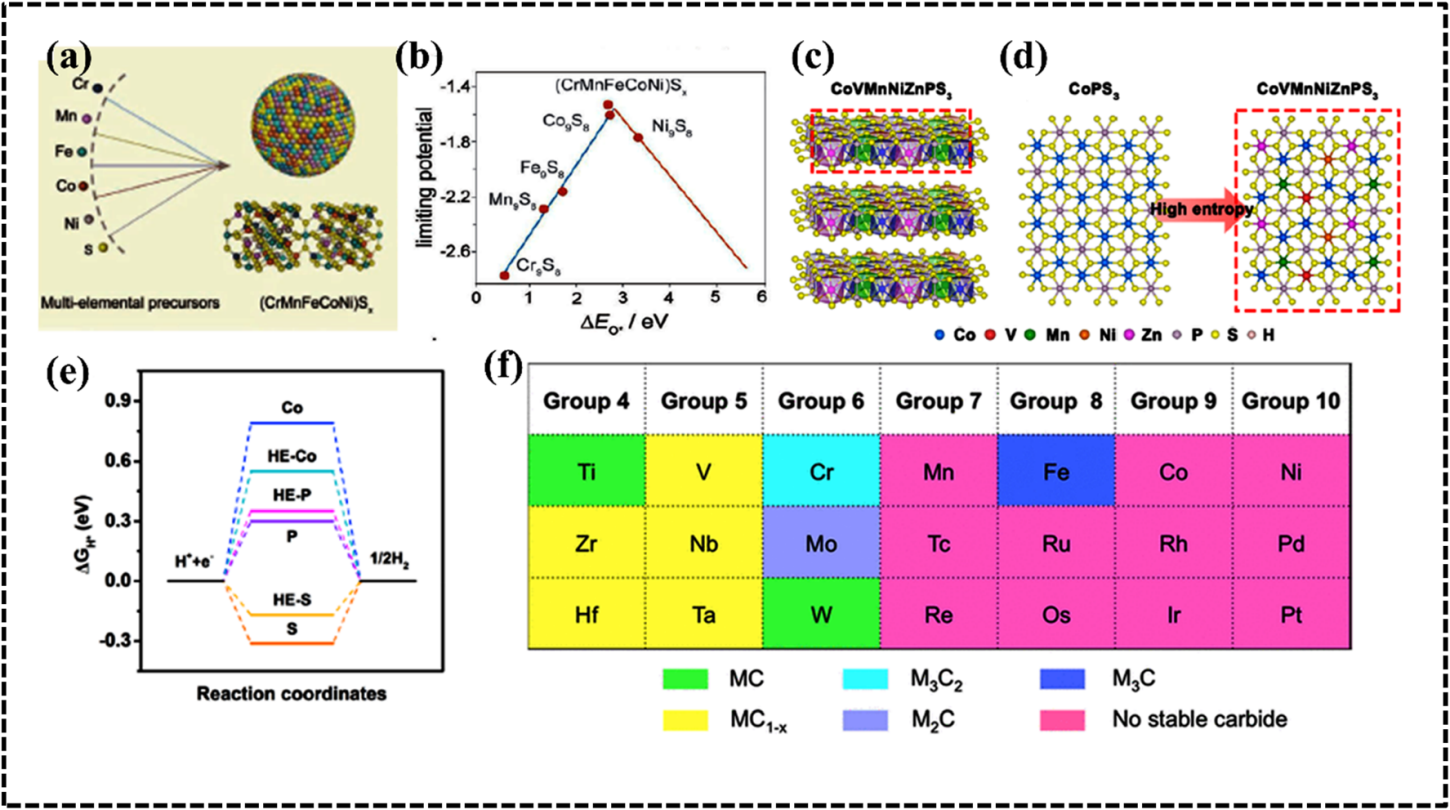
(a) Schematic demonstrating the structure and activity of high-entropy metal sulfide ((CrMnFeCoNi)S_*x*_) nanoparticles. (b) Calculated catalytic activity volcano plot. Reproduced with permission.^147^ Copyright 2021, Wiley-VCH. (c) DFT calculation in the edge mode; crystal structure of the monoclinic structure, (d) structural polymorphs of CoPS_3_ and CoVMnNiZnPS_3_ along the top views, and (e) hydrogen evolution reaction free-energy diagram of the corresponding edge sites. Reproduced with permission.^155^ Copyright 2022, American Chemical Society. (f) Elements in the synthesized high-entropy carbide (HEC) system. Reproduced with permission.^166^ Copyright 2013, American Chemical Society.

The stability and elemental tunability of single transition metal-based sulfide catalysts for the OER can vary significantly depending on the specific metal, synthesis method, and operating conditions. However, high entropy sulfides (HESs) can benefit from the stability of solid solutions with high mixing entropy.^147–151^ Cui *et al.* introduced a pulsed thermal decomposition method to synthesize high-entropy cubic (*Fm*3̄*m*) (CrMnFeCoNi)_9_S_8_ (Fig. 10a).This high-entropy sulfide exhibited promising OER activity and stability owing to the high configurational entropy resulting from the presence of multiple components (Fig. 10b).^147^ A wurtzite-type HES (Zn_0.25_Co_0.22_Cu_0.28_In_0.16_Ga_0.11_S) was developed by McCormick *et al. via* a simultaneous multi-cation exchange synthetic route.^150^ HESs with their large number of randomly distributed elements, possess unique catalytic, electrochemical, and mechanical properties. The choice of the metal significantly influences the electrocatalytic performance since they serve as active centers in the electrochemical process. The OER performance of quaternary HESs was improved by the selective choice of Mn and Al metals, which can be attributed to the strong interaction among the multiple metals and sulfur that regulates the electronic structure of the overall HES.^148^ The phase structure of the HES can be optimized by using the metal-to-sulfur ratio, and a decrease in the metal-to-sulfur ratio increases the possibility of single-phase HES formation, as confirmed by the mechanochemical synthesis of (FeMnNiCoCr)S_2_, (FeMnNiCoCr)S_2_, (FeMnNiTiCr)S_2_, and (FeMnNiCoCu)S_2_. Interestingly, all these materials are used as active anode electrodes for Li-ion batteries (LIBs) by following intercalation and deintercalation mechanisms.^149^ Typically, most of the synthesized HESs can be potential candidates for OER electrodes because of the favorable formation prospect of (oxy)hydroxide intermediates.^147–149,152^

#### High-entropy phosphides

The larger radius of the phosphorus atom in metal phosphates results in unique electronic states and surface structures as compared to those of common metal oxides. The uncoordinated surface atoms in high-entropy phases (HEPs) likely contribute to their enhanced catalytic activity through modification of their electronic structure. This hypothesis is supported by energy-dispersive X-ray spectrometry (EDX) analysis of single-phase Co, Cr, Fe, Mn, Ni-based HEP nanosheets, which revealed a uniform elemental distribution and contribute to a synergistic effect during water electrocatalysis.^153^ Wang *et al.* pioneered the preparation of high-entropy phosphate/carbon hybrid nanosheets using high-entropy MOF nanosheets as a precursor to synthesize (W_28_Ni_24_Co_24_Mo_17_Ru_7_)PO_*x*_/C with uniform distribution of the elements throughout the structure.^154^ To achieve optimal performance, high-temperature annealing and exfoliation were employed to synthesize Co_0.6_(VMnNiZn)_0.4_PS_3_ nanosheets with a monoclinic structure and high surface area (Fig. 10c and d). Experimental and theoretical studies suggested that the optimized S sites on the edge and P sites on the basal plane provide more active sites for hydrogen adsorption, whereas the introduced Mn sites enhance the hydrogen evolution reaction (HER) by augmenting the H adsorption Volmer step (Fig. 10e).^155^ Therefore, appropriate structural and electronic arrangement can successfully enhance the performance of an HEP-based electrode material.

#### High-entropy carbides

Transition-metal carbides have attracted attention as new active materials in various fields such as heterogeneous catalysis in energy conversion and electrode materials for energy storage processes owing to their superior electrical and thermal conductivity, earth-abundant characteristics, noble-metal-like surface reactivity, and good chemical stability under harsh operation conditions. High-entropy metal carbides (HECbs) with more than five metal ions exhibit higher negative free energy values in the electrochemical process. However, their preparation is difficult because multiple metal cations are not miscible in a single carbide solid solution. Braic *et al.* introduced HECbs in 2010 in the form of a thin (TiAlCrNbY)C film *via* the reactive magnetron sputtering method.^156^ Several methods are used for synthesizing HECbs, including ball milling, arc melting, spark plasma sintering (SPS), mechanical alloying, chemical vapor deposition (CVD), and hydrothermal synthesis.^157–165^ The choice of the synthesis method depends on the desired properties in the HECb and synthesis approach. However, the designing of high-entropy metal carbides is challenging because multiple metal cations with different ionic radii and coulombic forces are usually immiscible in a single solid solution. However, selectively designing transition-metal-based HECbs could be an effective strategy for energy storage and energy conversion processes (Fig. 10f).^166^ Despite the limited research on HECb-based electrode materials, their potential remains vast owing to several promising factors. The electronic interaction between carbon and the base metal alters the density of states (DOS) near the Fermi level, modifying the electronic properties of the HECb. Additionally, carbon introduction induces tensile strain, increasing the metal–metal distance and altering the d-band structure regulating the electrocatalytic performance. Moreover, surface carbon atoms may reduce available metal sites for adsorption and the reaction, potentially affecting electrode activity.

#### Other high-entropy anionic systems

HESis, HEPO_4_s, HEBs, HEONs, HECNs, and high-entropy boron carbonates have been extensively studied as HEA-based materials over the past decade. Several HEA-based derivatives and HECs exhibit superior thermal stability, oxidation resistance, electrical conductivity, and hardness, making them suitable for coating materials, circuit electrode film materials, and photocatalysts.^167–174^ Notably, the anionic arrays within high-entropy systems play a crucial role in electrochemical applications, whereas their counterparts in low-entropy, medium-entropy, binary, and even ternary alloys do not. This observation suggests that the anionic arrangement within the high-entropy structure significantly influences electrochemical properties. The derivative of a HEA refers to any alloy derived from the original HEA composition by adding, removing, or substituting elements. Successful modification of high-entropy alloys (HEAs) can lead to high-entropy materials (HEMs) with enhanced industrial performance as electrocatalysts. Therefore, the HEM concept is employed to define this unique class of material, and the precise contribution of anionic arrays controlling the electrochemical performance of HEMs remains an area of active research.

### Structural variation

**Fig. 11:**
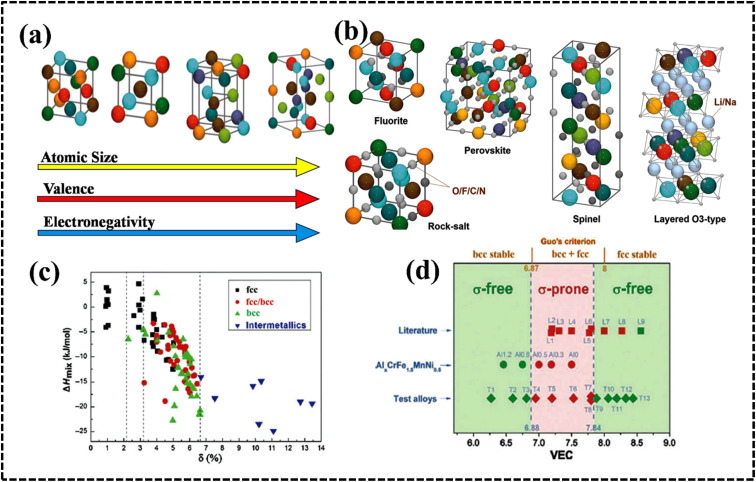
Identified crystal structures of (a) HEAs and (b) HECs used in energy-related fields. Reproduced with permission.^177^ Copyright 2021, Royal Society of Chemistry. (c) Crystal structures and their correlation with the enthalpy of mixing (Δ*H*_mix_) and atomic-size difference (*δ*) in various HEAs. Reproduced with permission.^80^ Copyright 2014, Spinger Nature. (d) VEC value with different phases of HEAs. Reproduced with permission.^179^ Copyright 2016, Elsevier Ltd.

The crystal structure of HEAs plays a crucial role in determining their electrocatalytic performance.^39,176^ This is because HEAs can exhibit diverse structures, ranging from single-phase solid solutions to amorphous phases (Fig. 11a and b).^177^ The phase structure of HEAs can be classified into three main categories based on the number of phases that constitute the overall microstructure: single-, mixed-, and amorphous-phase HEAs.^175–178^ Hume-Rothery rules suggest that *δ*, *Ω*, VEC, and electrons per atom (e/a) ratio play crucial roles in determining the type of phase, its stability, and physical properties.^39,175,178,179^ Analysis of phase formation using the parameters *Ω* and *δ* for various reported multicomponent alloys suggests a new criterion for solid solution phase formation in HEAs (Fig. 11c).^39,180^ The *δ versus* Δ*H*_mix_ plot suggests the possibility for the formation of FCC, BCC, or FCC + BCC structures by assuming solid solution conditions with an appropriate Δ*H*_mix_ value.^179^Fig. 11d shows the phase structure from the VEC values to understand:^39,180–182^

(a) FCC structure for VEC ≥ 8,

(b) BCC structure for VEC < 6.87, and

(c) mixed FCC + BCC structure for 6.87 ≤ VEC < 8.

The presence of Group 13 elements such as Al with larger atomic sizes than those of the principal elements generates less atomic packing and selective transformation of the FCC to the BCC structure. Above all, Al strongly reduces the local energy of the transition-metal atoms by p–d hybridization, which determines the overall structure of the HEA.^184,185^ Therefore, the addition of certain metals leads to a specific structure determined by the periodic placement of the metal, altering the original structure (BCC, FCC, and HCP).^183,184^ In HEAs such as Al_*x*_CrFeCoNi, the local energies suggest that Cr and Fe stabilize the BCC structure, whereas Co and Ni act as FCC stabilizers.

Interestingly, the structural features have no direct impact on the electrochemical performance of the HEAs as electrocatalysts or electrode materials. However, they exhibit an indirect influence on the strategic design of HEAs as they guide the selection of compatible metals from a structural perspective. Interestingly, BCC single-phase structures have attracted extensive attention in several fields, including hydrogen storage, owing to their unique structural characteristics because of their diverse composition, designable phase structure, and superior performance.^186–188^ The three primary crystal structures (BCC, FCC, and HCP) of HEAs can significantly impact their performance as electrode materials in electrochemical processes. BCC HEAs typically exhibit high strength and hardness but are also relatively brittle. They are more prone to forming passive films on their surfaces, enhancing their corrosion resistance. In addition, BCC HEAs are more susceptible to hydrogen embrittlement, compromising their toughness and ductility. FCC HEAs are generally more ductile and tough than BCC HEAs but less likely to form passive films.^184,187,188^ They are more resistant to hydrogen embrittlement and may exhibit favorable electrochemical performance in applications such as batteries, fuel cells, and electrochemical sensors. Consequently, FCC HEAs are preferred for energy storage applications owing to their high ductility and toughness. These are crucial parameters as batteries undergo significant volume changes during charging and discharging cycles. FCC HEAs have also demonstrated favorable Li-ion diffusion kinetics.^175–179^ HCP HEAs, which are less common than BCC and FCC HEAs, often exhibit properties that combine those of BCC and FCC HEAs. They can be strong and ductile while also resistant to hydrogen embrittlement (Table 2). However, HCP HEAs are susceptible to corrosion, which can be beneficial for electrochemical sensors. HCP HEAs are less likely to form passive films, a property that is advantageous when developing electrode materials for fuel cells. The good electrical conductivity and corrosion resistance properties of HCP- and FCC-based materials make them promising candidates for electrochemical sensors. Moreover, the large interstitial space in the BCC structure enhances ion diffusion performance, making it a potential electrode material for energy storage applications where charge is stored *via* ion diffusion.^39,179^

**Table 2:** Summary of the general electrochemical performance of body-centered cubic, face-centered cubic, and hexagonal closest packed high-entropy alloys (HEAs)

Crystal structure	Strength	Toughness	Ductility	Ion diffusion	Corrosion resistance
FCC	Medium	High	High	Medium	Medium
BCC	High	Low	Low	High	High
HCP	Medium	Medium	Medium	Low	Low

The presence of different crystal structures, such as FCC, BCC, and HCP, changes the electronic structure and creates distinct active sites, affecting the adsorption of reactant molecules and reaction kinetics. Therefore, while the electrochemical performance of a HEA is influenced by its crystal structure, the composition of the HEA, the specific environment, and the intended electrochemical application also play an important role in determining the behavior of each structure. Researchers often modify the composition and crystal structure of HEAs to take advantage of the unique properties of each crystal structure to optimize the electrochemical performance for a particular application. Certain crystals can provide exposed active sites of catalytically active metals or active crystal planes in certain electrochemical processes, and thus crystal structures can enhance the performance of HEA systems. For example, Cu atoms are stabilized by other metals in the FCC-facet crystalline structure of the AuAgPtPdCu HEA, which has been used as a potential electrode material for the CO_2_ reduction reaction (CO_2_RR).^189^ Another report suggested that a HEA with a Ni_20_Fe_20_Mo_10_Co_35_Cr_15_ FCC crystal structure has a greater number of coordination sites than that of the dual-phase structure (FCC+μ) and exhibited better HER catalytic performance in both acidic and alkaline media.^190–193^ Single-phase HEAs are more catalytically active than mixed or multiphase materials owing to their more exposed active sites. Therefore, the crystalline nature of HEAs has an influence on the electrochemical activity; consequently, strategic designing of HEAs is achievable by understanding their applicability and possibilities (Table 3).^186–200^ HECs can exhibit a variety of crystal structures, depending on the composition of the material and the processing conditions (Table 4).^201–213^ These varied crystal structures depend on metal ions and the counter anion 3d spatial arrangement of HECs (Fig. 11b).^177^ The crystal structure of HECs can significantly impact their electrochemical behavior and applications.

**Table 3:** Phase structure and electrochemical performance

HEA	Phase	Synthesis method	Electrochemical process	Reference
PtFeCoNiCuAg	FCC	Sputter	MOR	186
IrOsReRhRu	HCP	Pyrolysis	MOR	187
AlCoCrFeNi	FCC + HCP	Dealloying	Corrosion resistance	188
AuAgPtPdCu	FCC	Mechanical milling	CO_2_RR	189
NiFeMoCoCr	FCC/FCC+μ	Arc melting	HER	190
FeCoPtPdIr	FCC	Moving bed pyrolysis	HER	191
IrPdPtRhRu	FCC	Polyol method	HER	192
MnFeCoNiCu	FCC	Solvothermal pyrolysis	OER	103
AlNiCoFeX (X = Mo, Nb, Cr)	FCC	Top-down synthesis	OER	194
CoFeLaNiPt	Amorphous	Electrosynthesis	WS	195
PtAuPdRhRu	FCC	Wet chemistry	WS	196
AlNiCoIrMo	FCC	Dealloying	WS	197
AlCuNiPtMn	FCC	Dealloying	ORR	198
PtPdFeCoNi	FCC	Carbothermal shock	ORR	199
Hollow RuIrFeCoNi	FCC	Droplet-to particle	Li–O_2_ battery cathode electrode	200

**Table 4:** Crystal structure and high-entropy ceramics

HECs	Structure	Crystallography	Reference
High-entropy oxide (HEO)	Rock salt	Cubic (*Fm*3̄*m*)	201
	Fluorite	A_2_B_2_O_7_ cubic (*Fm*3̄*m*)	202
	Perovskite	ABO_3_ orthorhombic (*Pbnm*)	203
	Perovskite	ABO_3_ cubic (*Pm*3*m*)	204
	Perovskite	ABO_3_ hexagonal (*P*6_3_/*mmc*)	205
	Spinel	AB_2_O_4_ cubic (*Fd*3̄*m*)	132 and 206
	Pyrochlore	A_2_B_2_O_7_ cubic (*Fd*3̄*m*)	207
High-entropy nitrides (HENs)	NaCl-type	Cubic (*Fd*3̄*m*)	208
High-entropy carbides (HECbs)	Rock-salt (NaCl-type)	Cubic (*Fd*3̄*m*)	209
High-entropy borides (HEBs)	AlB_2_	Hexagonal (*P*6/*mmm*)	169
High-entropy silicides (HESis)	CrSi_2_ prototype	Hexagonal (*P*6_2_22)	211
High-entropy fluorides (HEFs)	Perovskite	ABF_3_ cubic (*Pm*3*m*)	145 and 212
High-entropy phosphates (HEPO_4_)	Monazite-type	Monoclinic (*P*21/*n*)	213

The properties of HECs, such as surface area, electronic structure, and reactivity, greatly affect their electrocatalytic performance.^194–197^ Furthermore, HECs with narrow band gaps tend to have higher electrocatalytic activity than those with wide band gaps. This is attributed to the easier electron transfer from HECs to reactants, which promotes electrochemical reactions. Furthermore, the reactivity of HECs plays an important role in their electrocatalytic performance (Table 5). It is important to note that factors such as composition, particle size, and microstructure also influence the electrocatalytic activity of HECs.^197–200^ Although research on HEC electrocatalysts is still in its early stages, current findings show great promise. HECs have a wide variety of potential applications, including energy production, fuel cells, and water electrolysis.

**Table 5:** Crystal structure and properties related to the electrochemical activity

Crystal structure	Electronic structure	Surface area	Reactivity	Electrocatalytic performance
Rock-salt	Narrow	High	High	High
Perovskite	Wide	Low	Medium	Medium
Fluorite	Wide	High	Low	Low
Spinel	Medium	Medium	Medium	Medium

### Computational and machine learning approaches

**Fig. 12:**
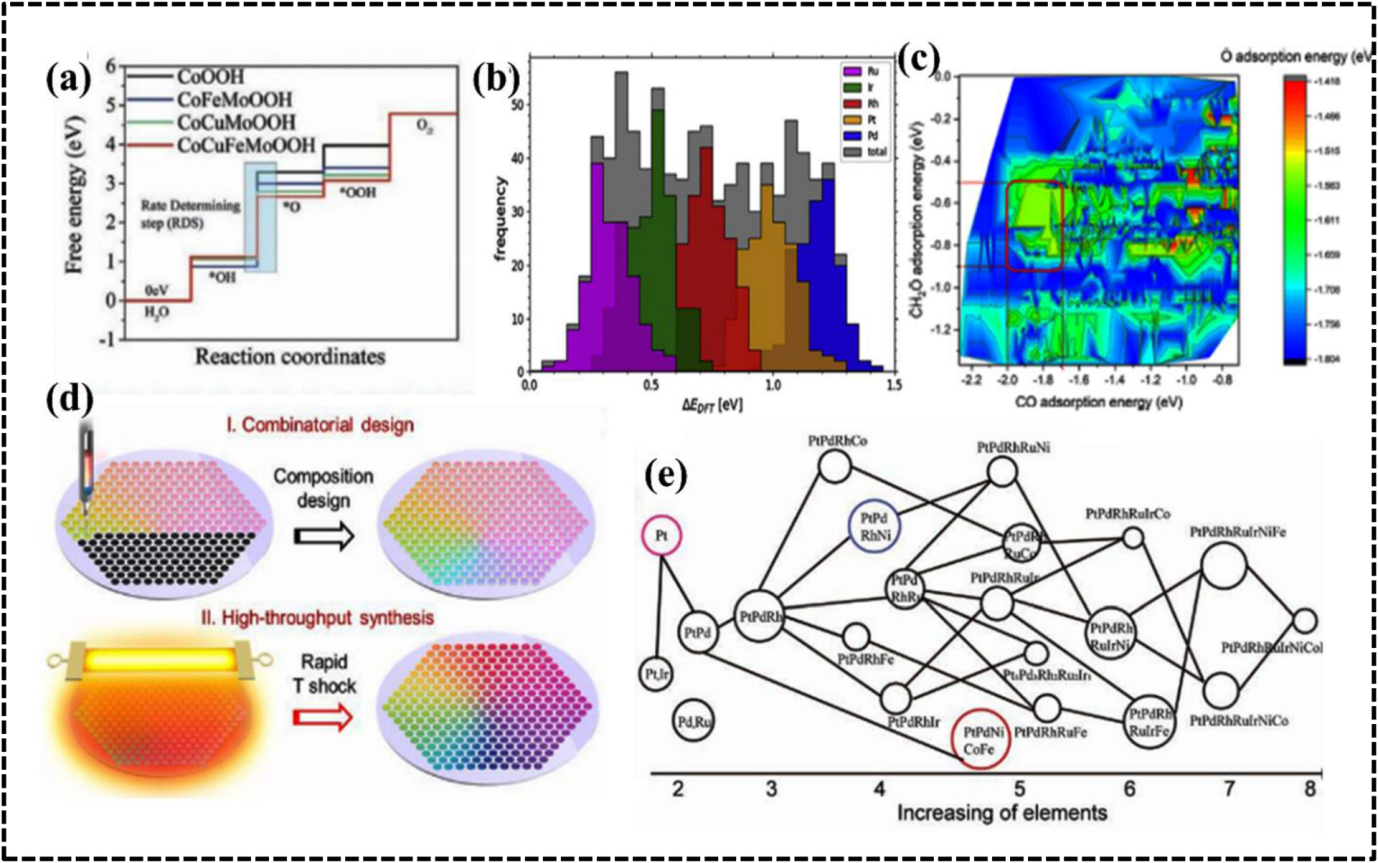
(a) Free energy diagram. Reproduced with permission.^219^ Copyright 2021, Wiley-VCH. (b) OH* adsorption energies. Reproduced with permission.^220^ Copyright 2019, Elsevier Ltd. (c) Contour plot for adsorption energy. Reproduced with permission.^226^ Copyright 2022, American Chemical Society. (d) Schematic illustration of the combinatorial and high-throughput synthesis of HEAs and (e) compositional designs and their corresponding oxygen reduction reaction (ORR) performances presented in a neural network diagram. Reproduced with permission.^199^ Copyright 2020, PNAS.

The compositional diversity in HEAs and high-entropy compounds has provided considerable opportunities to design potential electrocatalysts for electrochemical processes. However, designing the optimal composition for an electrode material through trial-and-error experimental approaches is a challenging task.^214^ HEA catalysts contain complex active sites because of their large compositional space and diverse atomic arrangement, necessitating a precise understanding of the active sites and optimal composition results. Owing to the vast number of compositional possibilities and combinations in HEAs, obtaining an optimal HEA system as an electrode material is challenging. With the rapid development of various experimental findings, methods to simultaneously obtain theoretical understanding through computational studies have emerged as benchmarking techniques to avoid trial-and-error approaches and achieve optimal HEAs with significant accuracy within a limited time frame. Moreover, considerable room for improvement exists in the structure–property–activity–performance relationship in HEA-based materials, which can be aided by understanding the reaction mechanisms and kinetics in terms of synthesis and activity. Material simulation has emerged as an intriguing method for the quantitative and qualitative evaluation of material properties at various scales, including specific insights into electrochemical application parameters.^215^ Several computational methods are available, including DFT, molecular dynamics (MD), discrete dislocation dynamics, the phase field method, the finite element method, and the thermodynamic method.^45,216,217^ HEA phase stability and phase diagrams can be obtained using DFT or MD, or they can be obtained from the Gibbs free energy calculations based on the first-principles calculations using phase diagram calculation methods. Interestingly, the calculation of phase diagrams (CALPHAD) method is an alternative technique for predicting equilibrium phases and a direct strategy for designing HEAs.^217,218^ However, the applicability of the CALPHAD method is limited by the accuracy of the database, and given the chemical complexity of HEMs, the DFT-based methods are inherently better suited for energy calculations. DFT analysis proves valuable in the design of quaternary high-entropy OOH catalysts, revealing those with the lowest adsorption free energy for the oxygen evolution reaction (OER) process (Fig. 12a).^219^ Computational studies have probed into free energy calculations of the reaction and provided an optimized structural overview of the steps that determine the reaction rate. In another study, the surface adsorption energies of Ir_0.102_Pd_0.320_Pt_0.093_Rh_0.196_Ru_0.289_ on oxygen reduction reaction (ORR) intermediates were evaluated by DFT calculations to optimize the adsorption and desorption capacities for OH* and O* intermediates.^220^ Therefore, optimizing the composition of HEA catalysts can be an efficient means of achieving optimal adsorption energies close to the peak of the Sabatier volcano curve for HER, OER, and ORR processes.^221^ This approach is also applicable for designing HEA systems for various electrochemical processes, including the CO_2_RR, the ammonia oxidation reaction (AOR), the nitrogen reduction reaction (NRR), and energy storage.^214–216,222^

In contrast, MD simulations are better suited for studying materials with multiple elements because they can handle larger systems with shorter computation times. However, a major limitation of MD simulations is that they disregard interatomic potentials, making it challenging to elucidate the complex chemistry of multicomponent ceramics. These methods have inherent limitations, which become even more complex when considering HEAs and their electrochemical performance. Challenges include high operational costs, inadequate experimental conditions, and hypothetical structures that lack thermodynamic stability. In recent years, the combination of big data and artificial intelligence (AI) has advanced owing to the progress of material genomics projects and rapid development of AI technologies.^214–216^ ML is a crucial subfield of AI that utilizes various algorithms to build models, uncover potential relationships from historical data, and design new HEA-based materials for specific applications, such as electrocatalysts.^223^ The ML approach involves rapid screening of a vast number of target property combinations required for catalytic applications. This can be accomplished through high-throughput (HT) experiments that combine computational and experimental methodologies.

The use of combinatorial synthesis is anticipated to expedite the discovery of new catalysts, enabling screening of a vast array of compositions in samples with tailored compositional gradients for HEAs, which are subsequently screened by machine learning to fabricate HEA-based electrodes. The optimization of existing catalysts and discovery of new catalysts are facilitated by valuable insights gained from the synthesis of new catalysts and theoretical calculations related to HEA-based electrocatalysts. An effective descriptor can expedite the development of ML models and elucidate the fundamental physical principles underlying the catalytic process. The ML approach can be employed to design HEA-based electrode materials for a wide range of industrial applications, including batteries, fuel cells, and sensors. This can contribute to the accelerated development of new and improved electrode materials with tailored properties.

A ML approach to develop HEA-based industrial electrode materials can be divided into the following steps: (a) data collection and preparation, (b) feature engineering, (c) model selection and training, and (d) model validation and deployment.^215^ ML is a powerful tool for accelerating catalyst discovery as it can be used to construct highly accurate models, predict the catalytic performance of uncharacterized catalysts, and elucidate structure–property–performance relationships, particularly for HEA catalysts with vast compositional spaces. The key to successful ML models lies in the utilization of suitable general descriptors that can accurately and comprehensively represent the structural information of the catalysts. An effective descriptor can expedite the development of ML models and uncover the fundamental physical nature of the catalytic process. HT techniques are essential for scientists to efficiently generate large databases and subsequently extract valuable information.

For HEAs with vast compositional spaces, HT techniques can be effectively employed to discover and develop HEAs. HT techniques can achieve more automated, parallel, and efficient HEA research.^224^ Singh *et al.* employed ML and DFT to predict the adsorption free energy of key reaction intermediates on HEAs, thereby quantitatively unifying the ligand (element identity) and coordination (surface structures) effects for HEA catalysts. A neural network (NN) model was utilized to evaluate the OH* adsorption energy over the IrPdPtRhRu HEA catalyst.^225^ These results suggest that electrochemical ability and structure–activity relationships of HEAs can be evaluated using ML *via* NN models. With the understanding of catalytically active sites, stability and activity are two crucial parameters for HEA-based electrocatalysts. ML tools have successfully identified the most promising composition with the highest catalytic stability and activity among Cu, Co, Ni, Zn, and Sn-based HEA alloys. The designed electrode materials have exhibited promising activity, selectivity, and stability toward CO_2_ hydrogenation to methanol. The DFT-calculated adsorption energies of the Cu (111) surface displayed good agreement with the ML-based results. Using screening criteria and considering the pure Cu (111) surface as a reference catalyst, contour plots were used to identify 35 active and selective catalysts for methanol formation, among which 34 were HEA catalysts and one was a medium-entropy alloy (Fig. 12c).^226^ Pedersen *et al.* proposed a method for discovering selective and active catalysts for the reduction of CO_2_ and CO on HEAs.^227^ The CO and H adsorption energies of all sites on the Cu (111) surfaces of disordered CoCuGaNiZn and AgAuCuPdPt HEAs were predicted by combining DFT with Gaussian process regression. This enabled the optimization of the HEA composition, increasing the likelihood that weak hydrogen-adsorbing sites can suppress the formation of molecular hydrogen.

The combination of a ML approach and experimental testing of PtFeCu NPs for the ORR was demonstrated by Chun *et al.*^228^ In the optimization of the PtFeCu alloy ratio, low PtFe and high Cu amounts exhibited the best catalytic performance among the ternary samples. Thus, *ab initio* computations with an ML approach can provide a solution for the design of nanocatalysts, thereby bridging the gap between the experiment and simulation. A HT combinatorial synthesis method yielded polymetallic HEAs with tailor-made surface chemistry that demonstrated significant potential for catalytic applications such as PtPdFeCoNi and PtPdRhNi HEAs as HER electrode materials (Fig. 12d and e).^119,199,228^

**Fig. 13:**
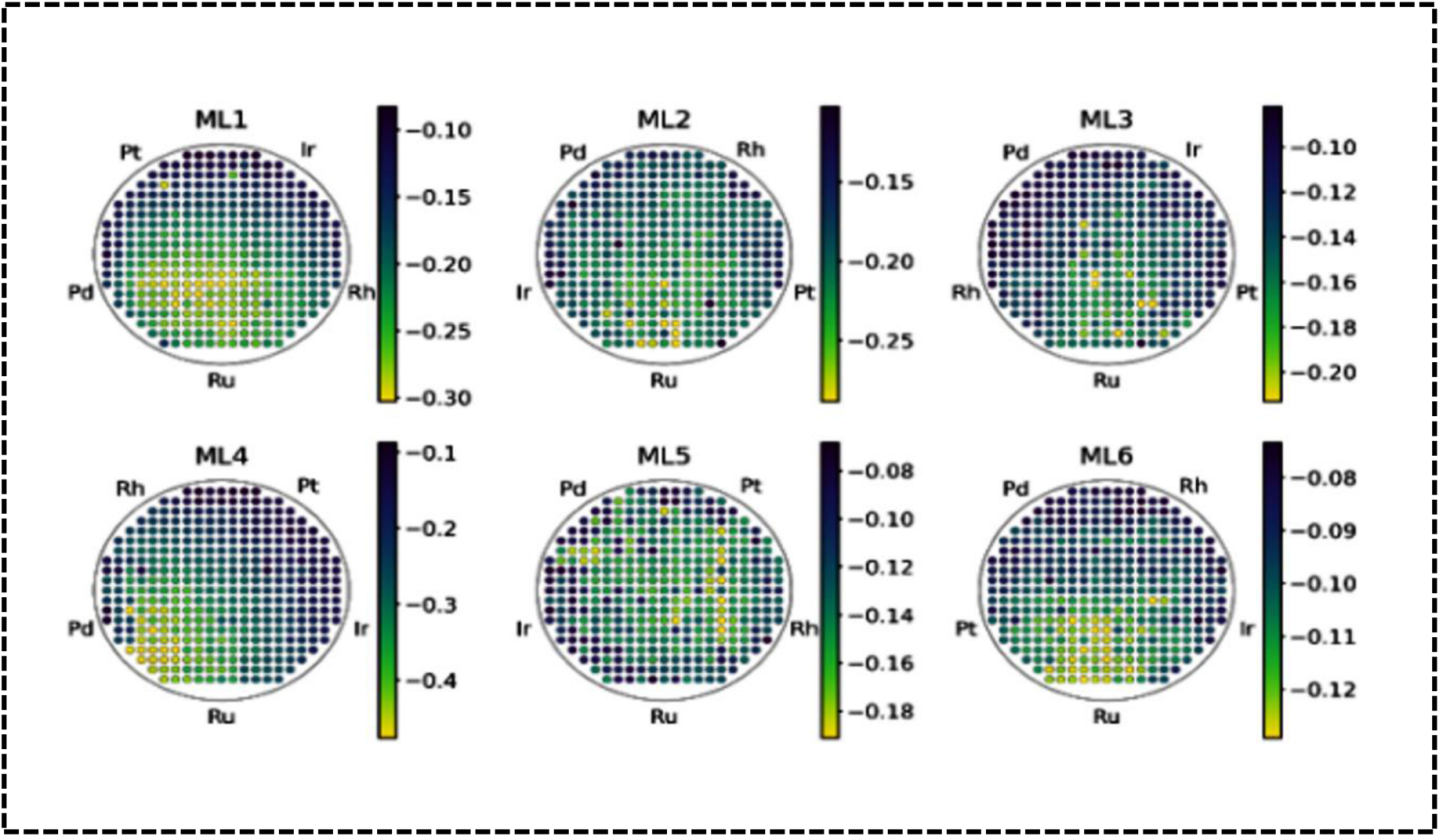
Activity map and high-throughput screening. Reproduced with permission.^230^ Copyright 2022, Wiley-VCH.

Recently, HT characterization datasets were used as an input for the refinement of the model. The refined model could correctly predict activity maxima of the Ag–Ir–Pd–Pt–Ru model system.^229^ The method has exhibited unprecedented power for the identification of optimal complex solid solution materials for electrocatalytic reactions. To identify complex solid solution compositions with high electrocatalytic ORR activity, they combined simulation, ML, data-driven combinatorial synthesis, and HT characterization. Banko *et al.* applied a combinatorial strategy to acquire large experimental data sets of 5D compositional spaces and the RuRhPdIrPt HEA system.^230^ Both advanced simulations (ML) and extensive experimental data analysis are used to estimate the electrocatalytic OER activity and solid solution stability trends in the 5D compositional space of the HEA system (Fig. 13a).^230^ Therefore, HT and ML models are two complementary approaches for designing the most promising HEA system for electrochemical studies. Before designing an industrial electrode material, it is essential to utilize theoretical and computational tools. These tools not only minimize time and chemical wastage but also aid in selecting the optimal route and composition for achieving the highest catalytic efficiency in HEA alloy-based electrode design.^218^

## Industrial application and synthesis strategies of HEAs

Industrial electrode materials are utilized across a broad spectrum of applications, including electrochemical cells, batteries, supercapacitors, fuel cells, and corrosion protection. Notably, the specific requirements for electrode materials can vary depending on the application and the desired performance characteristics of the electrochemical device. Therefore, material selection often involves a meticulous balance of these criteria to meet the particular requirements of each application.

### Selection, key properties, and designing strategy of HEAs as industrial electrode materials

The suitability of HEAs as industrial electrode materials relies on the specific application and desired performance criteria. Factors such as electrical conductivity, thermal stability, substrate adsorption capability, tuned active sites, anticorrosion performance, efficient mass transport ability, high surface area, structural complexity, and catalytic stability are considered determining factors for an ideal industrial electrode material. The ability to adjust the composition and carefully select the constituent elements of HEAs shows substantial promise in the development of potential electrocatalysts for industrial use. A rational design strategy is essential to improve catalytic performances that can only be achieved by appropriate selection and compositional integrity of the constituent elements. Before designing HEA-based electrode materials for industrial applications, several factors must be considered as follows: (a) the designed HEAs should favor a solid solution phase and avoid amorphous and or multiple phases, (b) the required physical and chemical properties that should be maintained after processing, (c) proper compositional integrity and stability should be achieved, and (d) homogenous features should be preserved throughout the material.^40^ Furthermore, the choice of elements is an important factor in the design of HEAs and its specific application. Periodic properties such as atomic radius, melting temperature, electronegativity, enthalpy of mixing, and electron energy level have influence on the formation possibility and chemical and physical properties of HEAs.

Table 6 shows the effect of specific periodic elements on the microstructure and properties.

**Table 6:** Influence of elemental properties on HEAs and their derivatives

Element	Physical property	Chemical property	Other
Fe	—	Change in magnetism	No effect on the solid solution phase or microstructure
Al	Increases porosity	—	Favors the FCC or BCC phase structure
Ti	Increases hardness and strength	Increases lattice distortion	Favors the BBC lattice structure
Zr	Reduces strength and plasticity	—	Intermetallic compound
V	Increases hardness and strength	Reduces oxidation resistance	Favors nanoparticle formation
Au	—	—	Cr, Mo, Fe, Ni, and Cu
Cu	—	—	Favors FCC solid solution
Ni	—	Paramagnetism	Favors FCC solid solution
Co	Improves wear resistance	Ferromagnetism	Favors FCC solid solution
Cr	Reduces strength and hardness		Favors BBC solid solution
Mo	Increases strength, hardness, and plasticity	Greater lattice distortion	Favors BBC solid solution
Mn	—	—	Reduces oxidation possibility

The utilization of HEAs is increasing with the strategic modification of the structure, electronic environment, and compositional integrity. Several designing strategies have been proposed to achieve potential activity, and they can be successfully exploited in industrial processes. The well-explored techniques are presented subsequently.

#### Size and morphology

The electrocatalytic performance of a catalyst heavily relies on its structure, morphology, surface area, and active sites. In this regard, different crystal structures (FCC, BCC, or HCP) can alter the electronic structure and active surface area, and distinct active sites affect the adsorption and reaction kinetics of the electrode materials. The electrochemical corrosion resistance ability of the AlCoCrFeNi HEA primarily depends on its crystal structure, with the FCC structure demonstrating improved corrosion resistance compared to the BCC structure.^231^ Stabilization of a specific crystal plane of a single element by other metals on the Cu (111) surface over the Au–Ag–Pt–Pd–Cu HEA was shown to significantly enhance both stability and activity, suggesting a potential stabilization and active center for the CO_2_RR process.^189^ However, morphology regulation is also an innovative approach to expose the active sites and increase the surface area of HEA-based electrode materials.

**Fig. 14:**
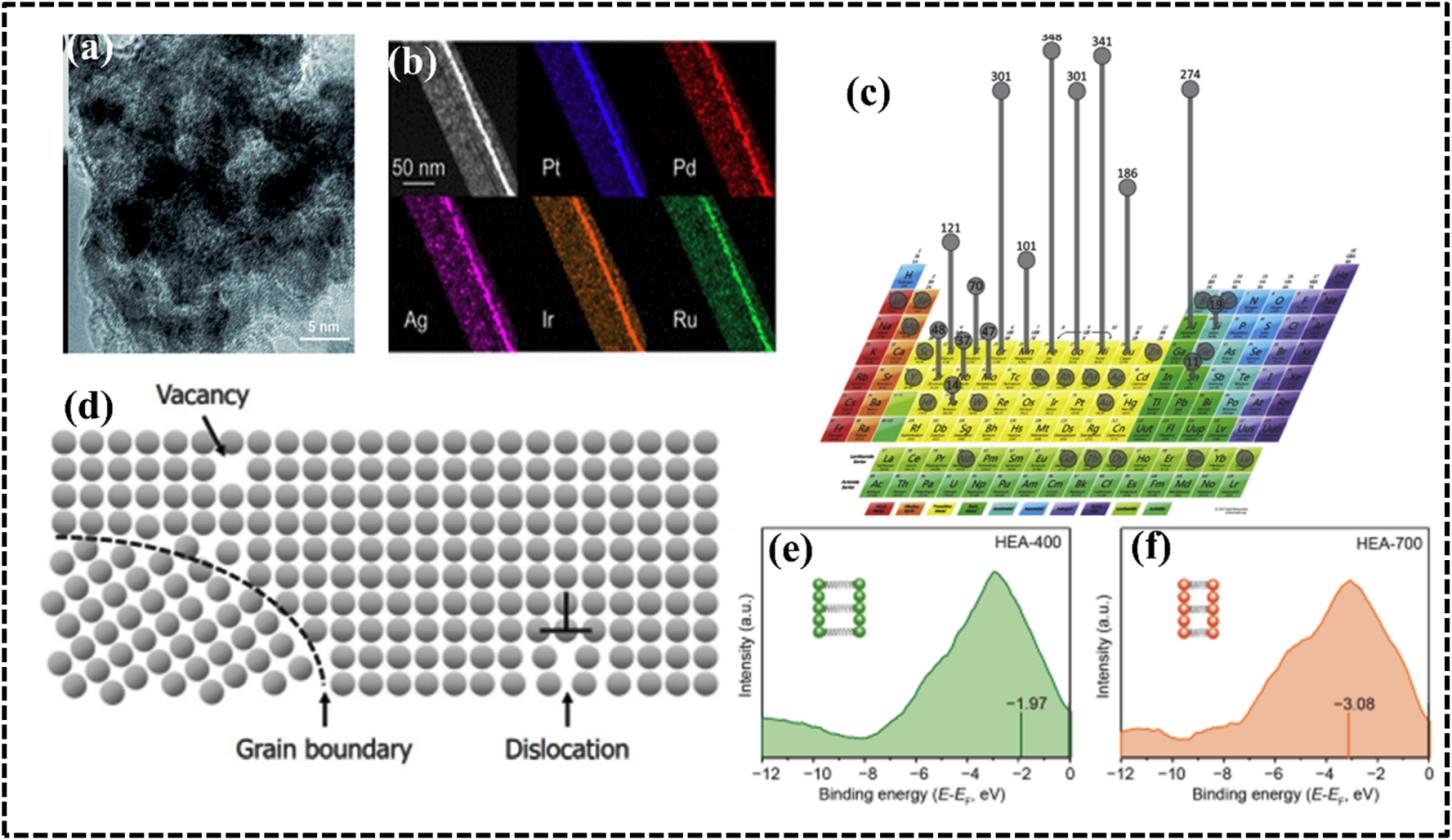
(a) Transmission electron microscopy (TEM) image of a HEA, Reproduced with permission.^234^ Copyright 2021, Royal Chemical Society. (b) High-angle annular dark-field scanning TEM (STEM) image and energy-dispersive X-ray spectroscopy (EDS) element mapping images of one segment of an HEA. Reproduced with permission.^235^ Copyright 2022, American Chemical Society. (c) Screening to design HEAs using frequency. Reproduced with permission.^236^ Copyright 2017, Elsevier Ltd. (d) Crystalline arrangements and consequences. Reproduced with permission.^241^ Copyright 2019, Elsevier Ltd. (e and f) Comparison of surface valence band photoemission spectra of HEAs. Reproduced with permission.^244^ Copyright 2021, Springer Nature.

The tri-functional electrocatalytic performance of convex-cube-shaped Pt_34_Fe_5_Ni_20_Cu_31_Mo_9_Ru nanocrystals is attributed to the formation of specific surface facets, strain, and coordination environments over the surface atoms of HEA catalysts.^232^ Usually, high-index facets are more active and exhibit higher catalytic activity than low-index facets owing to the cleavage and formation of chemical bonds. Along with morphology, particle size of the HEA has played an important role in adsorption and desorption processes at the electrode–electrolyte interface. Homogenous distribution and tuned particle size of the HEA can improve the electrode performance by improving the catalytic selectivity by altering the reactant or intermediate adsorption configuration. The orbital hybridization and overall charge transfer between metals and reactants exhibit a pronounced effect on the electronic structure of HEA NPs, which enhanced the electrocatalytic performance of PtRuNiCoFeMo HEA nanowires, resulting in high mass and specific activities for alkaline hydrogen oxidation reaction (HOR).^233^ Huang *et al.* synthesized defect-rich HEA nanowires that can achieve the electron transfer effect within the lattice structure.^233^ A 14-element nanoporous HEA (comprising Al, Ag, Au, Co, Cu, Fe, Ir, Mo, Ni, Pd, Pt, Rh, Ru, and Ti) was synthesized using the dealloying method, which was used as it can accommodate a far greater number of elements, resulting in a stable electrode material for water electrolysis.^234^ Transmission electron microscopy (TEM) analysis of the alloys showed a hierarchical, nanoporous, and nanocrystalline FCC structure with uniformly distributed elements; besides, Al leaching increased the number of accessible active sites on the surface structure (Fig. 14a).^234^ Recently, Guo *et al.* proposed a new technique to improve faradaic efficiency by forming ultrathin noble-metal-based PtPdIrRuAg HEA sub-nanometer ribbons *via* galvanic exchange reactions between different metal precursors and Ag nanowire templates (Fig. 14b).^235^ The 2D structure of the PtPdIrRuAg HEA provides large specific surface area, abundant active sites, and high density of unsaturated atoms, making it a potential candidate for the ORR.

#### Compositional regulation

Compositional integrity and the formation of CCSSs are primary requirements for attaining better electrocatalytic performance of HEAs. The electrocatalytic performance of HEA systems can be augmented by regulating the composition of the constituent elements. Despite the empirical nature of most HEA research, a rational approach for selecting specific metals is crucial for further advancement in this field. A comprehensive understanding of the interplay between HEA composition and catalytic activity is essential to address this limitation. This knowledge will enable informed decisions regarding elemental composition and facilitate rational component design of HEAs, ultimately leading to optimized adsorption energies of key intermediates on the alloy surface and enhanced catalyst activity, selectivity, and stability.

Miracle *et al.* summarized the frequency of use of each element in an HEA and confirmed that transition metals are the most frequently used and provide superior electrocatalytic performance (Fig. 14c).^236^ In particular, energy conversion processes require bifunctional catalysts and a HEA is undoubtedly a good choice. The systematic removal of each element from a Cr–Mn–Fe–Co–Ni quinary alloy system was shown to result in a significant lowering of ORR activity, thus indicating the importance of the synergistic combination of all five elements; this occurred probably because of the formation of a single solid solution phase with altered properties, which aids in overcoming the limitations that occur when using only single elements. The formation of single-solution and homogeneous distributions promotes the development of more active sites over the surface of HEAs, concurrently enhancing their catalytic activity. However, careful selection and compositional optimization are necessary to improve single-element lamination by increasing the number of active sites. The variation of Mn content in the Cr–Mn–Fe–Co–N alloy was experimentally confirmed to lead to superior intrinsic catalytic activity for the ORR.^237^ These findings underscore the significance of synergistic interactions among the five or more components in the HEA system and the resulting alterations in their electronic properties, overcoming the limitations of individual components.

Thus, multicomponent design strategies offer boundless possibilities for developing advanced catalysts. The synergistic interaction within the HEA system can be enhanced by augmenting the elemental diversity through compositional adjustments in the designed HEA system. An improved AOR performance was observed in senary RuRhPdOsIrPt alloy NPs when compared to ternary PdPtRh, quaternary IrPdPtRh, and quinary IrPdPtRhRu systems, suggesting that catalytic activity increases gradually with the number of components, thereby highlighting the importance of synergistically incorporating multiple metal elements.^238^ The catalytic performance can be varied by selecting appropriate design methodologies and optimizing the number of elements of the HEA system. Fujita *et al.* observed that HER and OER performances varied with the optimized compositional, structural, and electronic characteristics. Specifically, their findings suggest that a 12-element HEA is more superior to 14-element HEA systems in terms of the HER rather than OER processes, which contradicts the other findings related to the significant improvement of HEA systems with the increase in the number of elements.^234^ In an optimized Co_0.25_Mo_0.45_Fe_0.1_Ni_0.1_Cu_0.1_ HEA nanostructure, the systematic regulation of the Co/Mo ratio could modulate the chemical and physical properties of the HEA, resulting in an improved catalytic performance for the NH_3_ decomposition process.^64^ A rational configuration of the HEA electrocatalyst can adjust the adsorption energy of intermediate species to enhance the reaction rate.^239^ Low-electronegativity Mn and high-electronegativity Cu were combined with base metals (Fe, Co, and Ni) to form FeCoNiCuMn HEA NPs, which resulted in strong local electron interactions owing to the difference in electronegativity values. The theoretical interpretation is that the inactive Cu moves to the electron-rich active sites, resulting in lower adsorption energies of reactants, intermediates, and products, which increase the HER and OER activities of the HEA.^240^ Therefore, by combining experimental and theoretical understanding with specific compositional outputs, optimized, active, stable, and efficient HEAs can be obtained.

#### Defect engineering

Defect engineering is a well-established approach to improve the catalytic performance by exposing more active sites and creation of unsaturated coordination sites. Perfect lattice positioning of elements during the growth of the HEA is impossible; therefore, defects are formed inside the lattice structure. The periodic positions of the different constituent elements result in variable chemical and physical properties that can concurrently create defects inside the solid solution system. This can occur in almost all HEA-based systems and cannot be neglected. However, the choice of elements and synthesis strategies may control the percentage of defects inside the HEA. However, studies are already proving that defect engineering can alter the electronic and chemical environments of the HEA, thereby significantly enhancing the electrochemical performance. Moreover, stoichiometric variations within the same HEA can result in defects in the CCSS; this may result in varied electronic structures and adsorption energies.

HEAs typically exhibit numerous surface defects, owing to the complexity of their structures as they are composed of five or more elements in equal or nearly equal concentrations. This high elemental diversity can lead to a variety of structural imperfections, such as vacancies, dislocations, and grain boundaries (Fig. 14d).^241^ Surface defects can be used to improve the catalytic activity of HEAs for the HER. For instance, Kruzic *et al.* developed a new high-entropy metallic glass *via* dealloying to create a nanoporous structure,^242^ as a result of the large specific surface area with defects. The theoretical studies confirm that surface defects play a key role in improving catalytic activity and act as nucleation sites for the formation of hydrogen bubbles, which is a crucial step and helps in reducing the energy barrier for the HER. Surface defect modification in HEAs is still at an early stage, but it has the potential to pave the way for the development of novel and enhanced HER catalysts based on HEAs. HEAs are susceptible to forming defects, including vacancies, dislocations, and stacking faults, which can enhance electrocatalytic activity by creating more exposed sites. With a deeper understanding of defect engineering, more efficient HEAs can be developed, showcasing enhanced properties and innovative functionalities.

#### Strain engineering

Strain engineering serves as a promising approach to enhance the electrocatalytic performance of HEAs. Strain, which is primarily caused by alterations in bond lengths, can modify the CNs and electron densities of surface atoms, thereby influencing surface energy and promoting the specific adsorption of target molecules. The introduction of lattice strain into HEAs can be achieved through various methods, including epitaxial growth, lattice mismatch, and mechanical deformation.

The occurrence of strain in HEAs has significant effects on their electrocatalytic performance. First, strain can alter the electronic structure of HEAs, making them more active for specific electrocatalytic reactions. For instance, strain can increase the DOS near the Fermi level, enhancing the activity of HEAs in reactions involving electron transfer. Second, strain can improve the stability of HEAs, making them more durable in electrocatalytic applications in harsh electrochemical processes in industry. The improved stability originates from the strain-induced alteration of atomic arrangements and the stabilization of active sites on the surface and subsurface. Third, strain can increase the number of active sites on the surface of HEAs, further improving their electrocatalytic performance. However, strain can induce the formation of dislocations and other defects on the HEA surface, which can serve as additional active sites for electrocatalytic reactions. The d-band center is significant for HEA electrocatalysis as it determines the strength of the interaction between the multimetallic catalyst surface and the reactant molecules. A stronger interaction between the HEA-based electrode surface and electrolyte leads to a lower energy barrier for the charge-transfer process, resulting in improved electrode activity. Thus, strain engineering is a promising strategy for improving the electrocatalytic performance of HEAs. By carefully controlling the strain in HEAs, new and improved electrocatalysts can be developed for a variety of applications. The work of Rossmeisl *et al.* provides a new understanding of how lattice distortion affects the adsorption energies of OH* and O* on IrPdPtRhRu and AgAuCuPdPt HEAs and is important for understanding the effects of distortion and its consequences.^243^

Lattice distortion effectively alleviates the impact of local strain on adsorption energies due to the relaxed atomic environment surrounding the bonded atoms. This suggests that the effect of local strain on HEA activities is minimal, and the broadening of the adsorption energy distribution primarily occurs because neighboring atoms disturb the electronic environment of the binding sites. This finding holds significant implications for the design of HEAs for catalytic applications. TEM and scanning TEM (STEM) analyses implied that strain-induced PtFeCoNiCu HEAs obtained *via* heat treatment at 700 °C (HEA-700) exhibited 0.94% more compressive strain than the HEA obtained at 400 °C (HEA-400).^244^ The enhanced methanol oxidation reaction (MOR) activity can be attributed to the shorter Pt–Pt bond distances resulting from the compressive strain of HEA-700. Furthermore, a comparison of the d-band centers of the HEAs in the surface valence band emission spectra demonstrated that the development of compressive strain lowered the d-band center, leading to improved catalytic performance at higher temperatures (Fig. 14e).^244^

Strain in HEAs can also affect surface morphology, forming strain-induced defects such as dislocations, vacancies, and grain boundaries. These defects may act as active sites for catalytic reactions, providing new reaction pathways or altering the adsorption behavior of reactants. In addition, strain-induced defects can also alter mass transport properties and charge-transfer kinetics, affecting the overall electrocatalytic activity as defects provide a heterogeneous surface for catalysis. For example, Huang *et al.* fabricated single-phase FCC MnFeCoNiCu HEA NPs of less than 5 nm.^233^ These NPs exhibited a highly deformed lattice, resulting in various defects (*e.g.*, twins, dislocations, and stacking faults) and act as active sites for the ORR.^245^ Strain engineering is a promising strategy for improving the electrocatalytic performance of HEAs. Although there are still some gaps in our understanding of the optimal methods for introducing and controlling strain in high-entropy alloys (HEAs), this approach has great potential for advancing the development of novel and improved HEA electrocatalysts.

#### Regulating the electronic structure

The electronic structure of a catalyst plays a crucial role in determining catalytic performance, particularly through factors such as d-band centers, modification of the bandgap, and enhancement of charge transfer at the electrode–electrolyte interface. Predictions by Norskov *et al.* suggested the involvement of d-centers in multi-metal catalysts based on transition metals, where parameters like the d-band center and adsorption energy of intermediates/reactants influence the activation energy of catalytic reactions.^246^ The HEA does not deviate from this rule and the position of the d-band center in the alloy can be easily adjusted by changing the compositional elements and corresponding concentration.

**Fig. 15:**
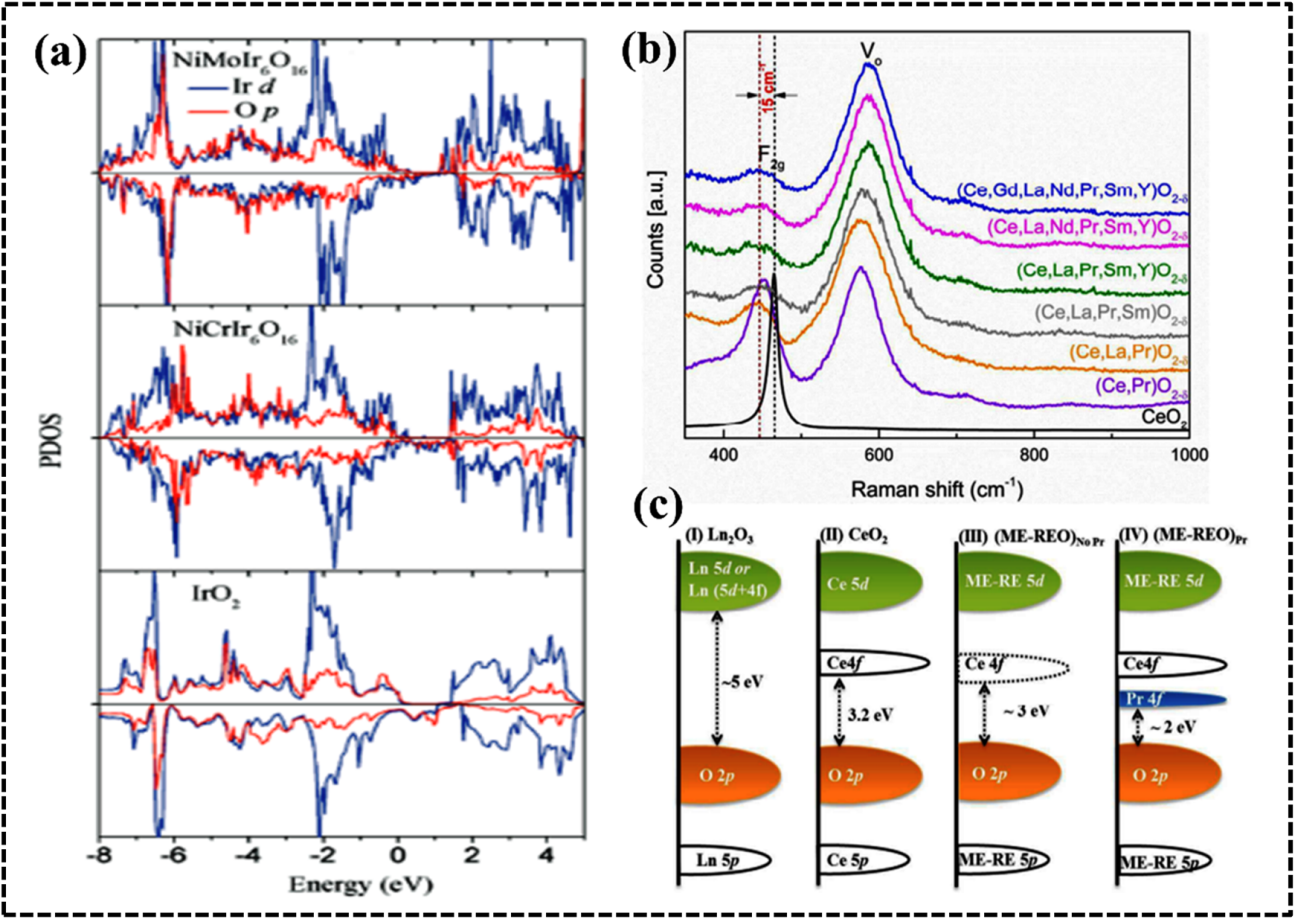
(a) Projected partial density of states of HEA d-states. Reproduced with permission.^197^ Copyright 2019, Wiley VHC. (b) Raman spectra. (c) Schematic of the band structure. Reproduced with permission.^254^ Copyright 2017, Royal Chemical Society.

An upward shift of the d-band center leads to stronger interactions within metals, while a downward shift represents the opposite. Additionally, the electronic structure of HEAs is influenced by the so-called ligand effect where the interactions between the d-bands of the constituent metals can alter the d-band centers of the active components.^246–248^ This modulation affects the binding energies of the key intermediates, ultimately improving the activity and selectivity.^71,247–249^ For instance, the projected DOS (PDOS) of nanoporous AlNiCoIrMo HEAs reveals a shift in the d-band center of Ir following alloy formation, leading to stronger Ir–O covalent bonds which further contribute to the enhanced OER activity. By strategically designing and optimizing the composition of HEA components, the adsorption energy of the d-band center can be continuously tuned, enabling the modulation of key intermediates and ultimately achieving optimal catalytic performance (Fig. 15a).^197^

The shift in the d-band center is a consequence of strain formation and simultaneously affects the charge-transfer ability and bandgap alteration within the compositionally complex HEAs.^250–253^ A lowering of the bandgap was observed by Sarkar *et al.* during the synthesis of fluorite-type rare-earth HEO. The presence of Pr with oxidation states other than 4+ results in the formation of oxygen vacancies in multicomponent equiatomic rare-earth oxides, which was confirmed by Raman spectroscopy and a reduction in bandgap due to electronic state involvement (Fig. 15b and c).^254^ The charge redistribution on the surface of HEAs is an inimitable and important property that can be used to enhance their catalytic activity.^255^ The alteration in local charge density generates active sites for the adsorption and activation of reactant molecules.^256–259^ Moreover, it influences the d-band centers of the alloy, thereby modifying the adsorption modes of reactants and further impacting catalytic activity and selectivity. HEAs, with their diverse compositional metals, possess the potential to exhibit pronounced surface charge redistribution, inducing an alternating pattern of electron accumulation and depletion across the entire HEA surface.^254–259^ This implies that the charge density of each surface metallic atom differs from that of its neighboring atoms in HEAs, creating a greater number of active centers for chemical transformations.^260^ Hence, careful selection of components with differing work functions for HEA synthesis allows for control over the surface charge density of HEAs. This contributes to the adsorption and activation of reactants, further enhancing the catalytic performance of HEA-based catalysts for industrial applications.

### Synthesis strategies and characterization of HEAs

Developing industrially viable high-entropy alloys (HEAs) and their derivatives poses a significant challenge due to their potential for electrocatalytic applications. These alloys offer tunable chemical and physical properties, including crystal structure, atomic size, valence electron configuration, and consequently, enthalpy and entropy of formation. This tunability makes them highly attractive for designing electrocatalysts with tailored properties for specific reactions. Controlling the size, composition, shape, morphology, and phase structure of HEA is a complex task owing to the inevitable migration and aggregation phenomena that occur under synthetic conditions. Therefore, a thorough understanding of synthesis techniques is crucial for designing and developing potential electrode materials. In general, the available and reported synthesis methods are broadly categorized as conventional, wet-chemical, and nonequilibrium synthesis methods (Table 7), which are discussed in the subsequent sections.^261–294^

**Table 7:** Overview of HEA synthesis methods

Synthesis	Equipment	Condition	Synthesis time	Size	Element distribution	Product percentage
Mechanical alloying	Planetary ball mill	RT, air	>1 day	Micrometer range	Poor	≥10 g
Spark discharge	Spark discharge	High voltage	Fraction of a second	Nano- to micrometer	Good	Above 80
UAWC	Ultrasonicator	Below 100 °C		Nanometer	Good	Above 70
Dealloying	Smelting furnace/Spinning machine	≤500–700 °C and 1 atm, both inert and air atmospheres	≥2 h and ≤5 h	Pore size within 10 nm	Good	≥10 g
One-pot synthesis	Hydrothermal reactor and furnace	High temperature, inert or air atmosphere	Few minutes to a day depending on the process	Variable	Good	≥10 g
Calcination-assisted method	Furnace	High temperature, above 500 °C	Few hours	Porous structure	Good	50–90
Electrodeposition method	Electrochemical set up	Room temperature, air	Few minutes to several hours (≤24 h)	Nanometre range	Good	≤10 mg
Sputtering deposition and electrospinning method	UHV deposition system	RT/Ultrahigh vacuum	>25 h	≤1–10 nm	Good	<200 mg
Carbothermal shock method	Current pulse device	1 atm and > 1000 K and an inert atmosphere	Millisecond	Nanometre range	Good	<200 mg
Fast moving bed pyrolysis	Tube furnace	≤1000 K and an inert atmosphere	Few seconds	5–50 nm	Good	∼100 mg
Laser scanning ablation technology	Pulsed laser	RT, air	Millisecond		Good	
Microwave heating	Microwave reactor	≤2000 K	Few seconds	≤20 nm	Good	≤10 mg

#### Mechanical alloying

**Fig. 16:**
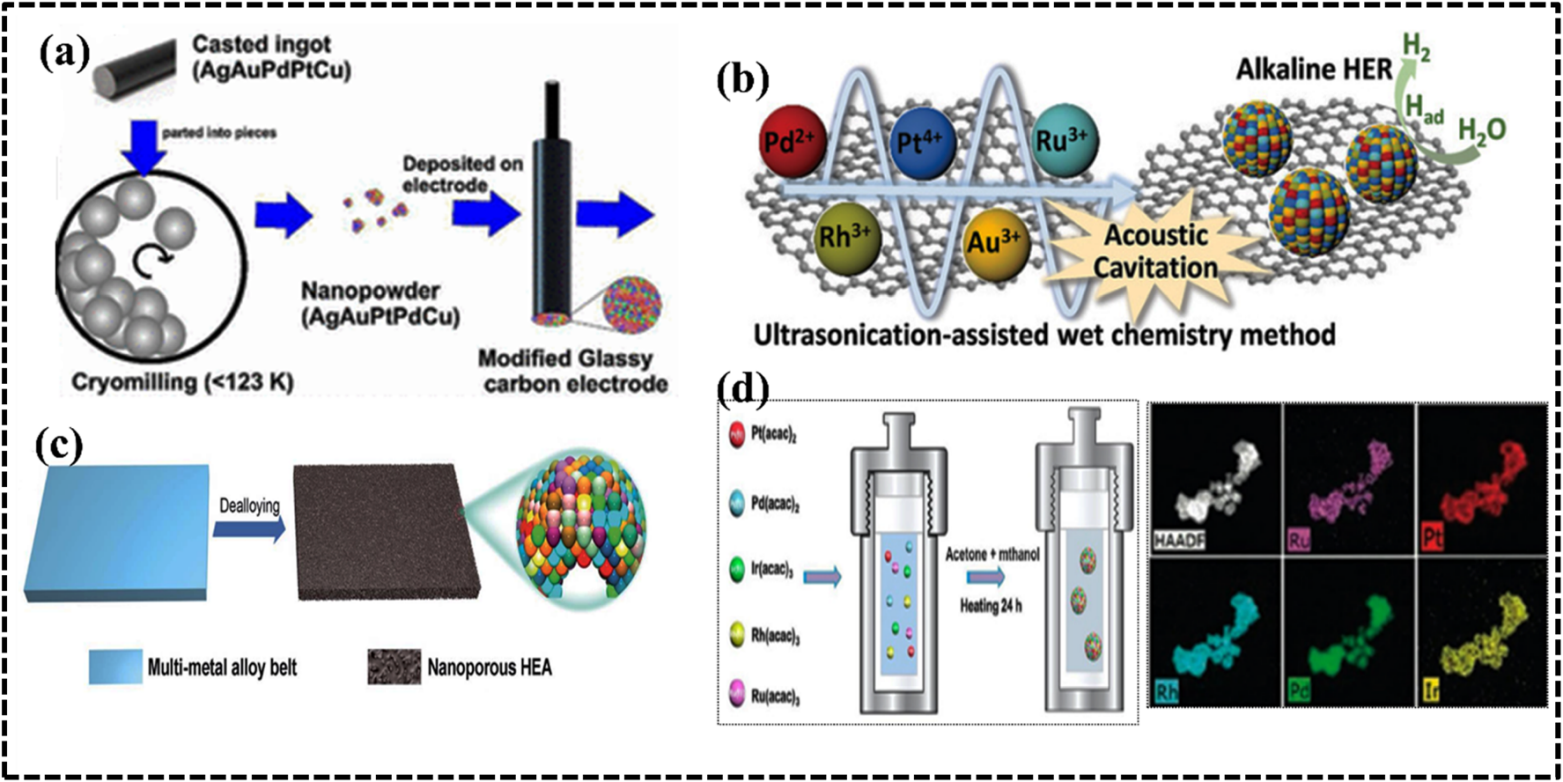
(a) Synthesis procedure of HEAs by cryomilling. Reproduced with permission.^266^ Copyright 2021, American Chemical Society. (b) Ultrasonic-assisted wet chemistry method for HEA design. Reproduced with permission.^268^ Copyright 2019, Wiley VCH. (c) Dealloying method for HEA design. Reproduced with permission.^234^ Copyright 2021, Royal Chemical Society. (d) One-pot synthesis procedure and STEM-EDS of the HEA system. Reproduced with permission.^275^ Copyright 2019, Wiley VCH.

Mechanical alloying (MA) employs high-energy forces in a closed container with a hard medium, pulverizing pure metal powders and inducing their solid-state transformation into homogenous HEAs.^261,262^ MA is a particularly attractive method as it can be used to mix elements with widely different chemical and physical properties that provide a wide range of compositions and microstructures.^262^ By adjusting the milling conditions, such as the milling speed, milling time, and ball-to-powder ratio, the grain size, phase composition, and other properties of the alloy can be controlled.^263,264^ Biswas *et al.* developed a cost-effective, low-temperature MA (cryomilling) technique for the preparation of HEA NPs (Cu_0.2_Ag_0.2_Au_0.2_Pt_0.2_Pd_0.2_).^265,266^ A similar synthesis approach was used to synthesize Ag, Au, Pt, Pd, and Cu-based HEA NPs with different compositions and compositional variations. Interestingly, the designed NPs showed intense activity against hydrazine electrolytic oxidation (Fig. 16a).^266^ Dai *et al.* successfully formed a HE-ZIF, confirming the superiority of the ball milling method over the thermal method when forming HEAs.^262^ Furthermore, the MA process is time and energy intensive, and several parameters must be optimized to obtain the desired properties, such as the milling time, milling speed, and ball-to-powder ratio. Therefore, further research is necessary to optimize the MA process and enhance the reproducibility of the resulting HEA powder, encompassing a wide range of compositions and microstructures, with the potential for largescale HEA production.

#### Spark discharge

Spark discharge (SD) is a plasma-based process that involves generating a high-voltage spark between two electrodes to generate high-temperature and high-pressure plasma. This plasma can be used to melt and vaporize elemental powders, which are then rapidly cooled to form HEA NPs. SD has several advantages compared to other HEA synthesis methods, including high purity, controllable microstructure, and high scalability. Wu *et al.* used the SD method to prepare CoCrFeNiPt and CoFeNiCr_0.5_Pd_0.8_ HEA NPs with uniform elemental distribution.^267^ First, HEA blocks were prepared *via* vacuum arc melting using metal elements as raw materials in a high-purity argon atmosphere; then, NPs were prepared using this HEA ingot as an electrode. SD is a promising technique for synthesizing HEAs with several desirable properties and is expected to play a promising role in the commercial production of HEAs.

#### Ultrasonic-assisted wet chemistry

Ultrasonic-assisted wet chemistry (UAWC) involves the use of ultrasound waves to generate cavitation bubbles in a solution containing metal precursors. UAWC exhibits several advantages in the synthesis of HEAs, including scalability, compositional and structural versatility, and simplicity.^196^ Parameters such as the power and frequency of the ultrasound waves are tuned to control the size and morphology of HEA NPs.^268–270^ Liu *et al.* utilized the acoustic cavitation phenomenon to synthesize PtAuPdRhRu HEA NPs in an ultrasonic irradiation process that operated at a significantly high temperature and pressure (5000 °C and 2000 atm) to accelerate the simultaneous reduction of metallic ions and form an entropy-maximized state (Fig. 16b).^268^ In comparison with other wet-chemical synthesis strategies, the UAWC method is advantageous when designing NPs.^269^ Hence, UAWC is a promising approach for the synthesis of HEAs with a variety of desirable properties and is expected to play an important role in commercial production of HEAs with potential electrode performance.

#### Dealloying strategy

The dealloying method has been widely utilized to fabricate a nanoporous HEA structure, providing an effective way to increase the specific surface area.^270^ Dealloying involves the selective removal of one or more elements from a solid solution alloy, leaving behind a porous structure. The precursor alloy is then dealloyed in a solution that selectively dissolves one or more of the elements; then, the remaining elements self-assemble into a porous HEA structure. Liu *et al.* employed a facile one-step electrochemistry dealloying process to etch the residual Mn to fabricate nanoporous NiCoFeMoMn.^271^ It is well documented that Mn can easily form solid solutions with other transition metals, although the standard redox potential of Mn is comparatively lower (Mn^2+^/Mn) than that of the other transition metals, resulting in easy corrosion in an acidic medium. An analogous synthesis route was employed to synthesize a free-standing nanoporous HEA containing 12 or 16 uniformly distributed metal elements (Mn, Ni, Cu, Co, V, Fe, Mo, Cr, Pd, Pt, Au, Ru, Ir, Ag, Rh, and Os) *via* one-step etching of Mn.^272^ In general alkaline solution was used to employ the dealloying strategy to design porous HEA solid solutions although the standard redox potential of Mn is comparatively lower (Mn^2+^/Mn) than that of the other transition metals, resulting in easy corrosion in an acidic medium.^272,273^ The design of a homogenous nanoporous HEA containing up to 14 elements (Al, Ag, Au, Co, Cu, Fe, Ir, Mo, Ni, Pd, Pt, Rh, Ru and Ti) was performed by Fujita *et al.* using the alkaline solution based dealloying method and it demonstrated good water electrolysis efficiency (Fig. 16c).^234^ An analogous synthesis route was employed to synthesize a free-standing nanoporous HEA containing 12 or 16 uniformly distributed metal elements (Mn, Ni, Cu, Co, V, Fe, Mo, Cr, Pd, Pt, Au, Ru, Ir, Ag, Rh, and Os) *via* one-step etching of Mn.^272^ The universality of this alloying/dealloying strategy should be further investigated to include other metallic elements and various combinations.

#### One-pot synthesis

The one-pot synthesis method is well known for its easy and cost-effective approach, which includes the regulation of the growth process by adjusting various synthesis parameters such as temperature, pressure, atmosphere, solvent, reducing agent, coordinating agent, and reaction time. Niu *et al.* synthesized nanosized CoCrCuNiAl HEAs using the sol–gel auto combustion method, resulting in a uniform distribution of the elements.^274^ This uniform distribution led to enhanced thermal, mechanical, and electrochemical properties achieved from compositional integrity and the synergistic effect of the HEA. In another report, Bondesgaard *et al.* used a low-temperature solvothermal method to synthesize PtPdIrRhRu HEA NPs (Fig. 16d).^275^ The interaction between metal and acetylacetonate controlled the precipitation rate, thereby promoting the co-precipitation phenomenon for the synthesis of single-phase NPs. Furthermore, controlling the crystal structure of HEA NPs also provides new avenues for promoting catalytic performance. The cost-effective co-precipitation technique is an easy synthesis approach to fabricate nanosized HEAs with uniform distribution. For instance, Zou *et al.* synthesized defect-rich 2D HEHs (FeCoNiZnAl, FeCoNiZnAlCuCr, and FeCoNiZnAlCuCrMgIn) using the co-precipitation method.^276^ Huang *et al.* established a solvothermal approach to prepare HEAs on carbon cloths that exhibited numerous partial dislocations, stacking faults, and atomic displacements, indicating a highly distorted atomic lattice within the NPs. These defects in the HEAs contributed to surface tension, which consequently impacted the electrocatalytic performance (Fig. 13h).^103^

#### Calcination-assisted method

HEA systems can be designed by using a calcination method in combination with other methods such as co-precipitation and hydro/solvothermal, and other wet-chemical methods. Owing to annealing, migration of the atoms and reconstruction of the nanostructure occur, leading to a more ideal microstructure. The annealing atmosphere, temperature, and time exhibit a significant influence on the structure of the HEA system.^277,278^ Chen *et al.* controlled the crystal structure of single-phase HEA NPs by altering the annealing temperature and inducing strain inside the lattice.^60^ However, a strain within the HEA is developed that significantly improves catalytic performance.^244^ Additionally, the enhanced compressive strain reduces the M–M bond, thereby shifting the d-band centers of the active metal downwards and enhancing its electrocatalytic activity.^279^

#### Electrodeposition method

**Fig. 17:**
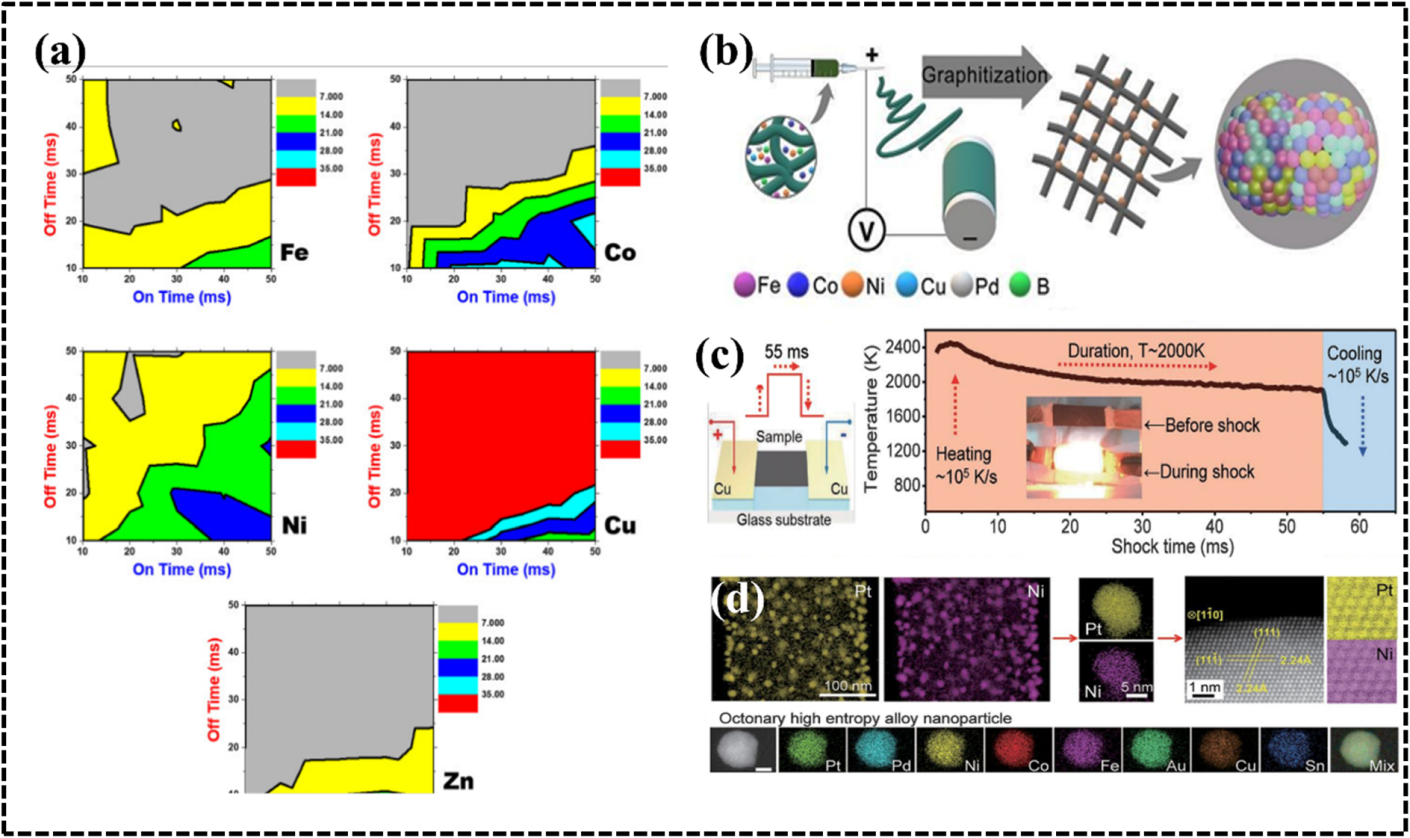
(a) Contour maps of the composition of different elements deposited at various *T*_on_ and *T*_off_ values. Reproduced with permission.^281^ Copyright 2023, Elsevier Ltd. (b) Electrochemical approach to synthesize HEAs. Reproduced with permission.^287^ Copyright 2023, The Royal Society of Chemistry. (c and d) Carbothermal shock synthesis of HEA nanoparticles. Reproduced with permission.^288^ Copyright 2018, AAAS.

Electrochemical deposition (ECD) offers several advantages such as low-temperature processing, short synthesis duration, simplified equipment design, and a high potential for technological scalability in the design of HEAs. In principle, ECD reduces metal ions from an electrolyte solution onto a substrate by applying an electric current and a HEA is grown over the current collector. The electrolyte composition and deposition parameters, such as current density and temperature, are carefully controlled to produce HEAs with the desired properties. A key challenge in the ECD of HEAs is to achieve co-deposition of all the constituent elements in the desired proportions. This is difficult because of the different electrochemical reduction potentials of the involved metals. However, a variety of strategies have been developed to overcome this challenge, such as the use of complexing agents, pulse electrodeposition, and alloying with a third element. Sure *et al.* synthesized nanosized equiatomic single-phase FCC CoCrFeNi HEA powder by performing electro-deoxidation in molten CaCl_2_–NaCl at 923 K.^280^ Their study demonstrated that the molten-salt-based electro-deoxidation process enables the synthesis of the CoCrFeNi HEA in the form of a nanocrystalline powder. Pulsed electrodeposition was proposed for better current distribution and mass transfer, resulting in a more suitable microstructure and chemical composition as compared to those of direct electrodeposition. The ECD technique was employed by Dey *et al.* to synthesize a Zn-based HEA (FeCoNiCuZn) in an aqueous medium utilizing sulfate salt where the duty cycle (*T*_on_ and *T*_off_) and pH play a crucial role in controlling the microstructure and composition of thin films.^281^ However, owing to the application of pulses during electrodeposition, the surface roughness of the substrate was observed to have negligible effects on the microstructure and composition (Fig. 17a).^281^ Moreover, during the electrodeposition of multiple elements, the *T*_on_ time must be higher than *T*_off_ for the simultaneous deposition of multiple elements as an alloy. Li *et al.* demonstrated an electrochemical approach to construct various nanoscale HEAs by rapidly changing the thermodynamic conditions and manipulating growth kinetics. The study found that a self-supporting dendritic multilevel Fe_0.22_Co_0.18_Ni_0.18_Cr_0.14_Cu_0.28_ HEA electrode composed of a single FCC phase exhibits superior electrocatalytic performance, which is attributed to the synergistic effect of multiple active sites and the unique foam-like hierarchical structure.^282^ Although this electrochemical deposition method has the advantages of low cost, easy availability of raw materials, and minimal equipment requirements, it may face challenges related to unstable voltages and currents, which can cause problems in nanomaterial preparation.

#### Sputtering deposition and electrospinning methods

Sputter deposition is a physical vapor deposition technique in which high-energy plasma is irradiated onto a target material that contains all the alloying elements. Notably, sputter deposition is a relatively time-consuming process and achieving a uniform composition of the deposited film is not easy, especially for HEAs containing several alloying elements. Wang *et al.* developed amorphous or nanocrystalline solid solution CrNbTiMoZr HEA films *via* DC magnetron sputtering.^283^ More recently, Alam *et al.* introduced radio-frequency magnetron sputtering to synthesize the TiMoVWCr HEA. The proposed system is fast and reproducible, and provides a high degree of freedom to synthesize a wide range of HEAs.^284^ Furthermore, the technique can be used to design thin-film HEAs and control the size and composition of the designed HEA system.

Electrospinning is an advanced, inexpensive, and extended technique of sputtering deposition and can be used to produce nanofibers from a variety of materials, including polymers, metals, and ceramics. To synthesize HEAs using electrospinning, a metal precursor solution is typically used that contains all the desired alloying elements in the anticipated proportions. The precursor solution is then electrospun to produce HEA nanofibers. However, note that the electrospinning process is sensitive to multiple factors, such as the type of solvent, precursor solution concentration, and applied voltage. Consequently, producing HEA nanofibers with a consistent composition and microstructure is difficult. By combining the electrospinning technique and graphitization process, Hao *et al.* synthesized FeCoNiMnRu HEA NPs with potential water electrolysis efficiency due to the modified electronic structure.^285^ The difference in electronegativity values between the mixed elements in the HEA induces significant charge redistribution, creating highly active Co and Ru sites with optimized energy barriers for simultaneous stabilization of OH* and H* intermediates, thereby significantly improving water dissociation efficiency under alkaline conditions.^285^ A similar approach was involved in the synthesis of FeCoNiMoRu HEA NPs supported on carbon nanofibers (CNFs). The *in situ* electrochemical Raman results demonstrated that the Fe, Co, Ni, and Ru metal sites are involved in synergistically catalyzing urea electro-oxidation.^286^ Dual-phase B-doped FeCoNiCuPd HEA (DP-B-HEA) NPs with superior NRR ability were *in situ* grown on electrospun CNFs *via* the thermodynamically driven solid-phase diffusion approach by combining the electrospinning technology with the high-temperature carbonization approach (Fig. 17b).^287^*In situ* characterization confirmed that CNFs were used as hosts to control the phase stability of DP-B-HEA NPs *via* the unique dual phase and electronic structure modification of B atoms. Electrospinning, a versatile technique for fabricating nanomaterials with tunable morphologies and properties, exhibits significant potential for the largescale production of HEAs. While HEA production by electrospinning is still in its early stages of development, it offers several advantages compared to conventional synthesis methods.

#### Carbothermal shock method

The carbothermal shock (CTS) method involves the reduction of metal oxides with carbon in a confined space at high temperature for the formation of HEAs. Yao *et al.* first proposed a simple two-step CTS method, in which the metal precursor was flash heated and cooled (temperature: ∼2000 K, shock time: ∼55 ms, ramp rate: 105 Ks^−1^) on an oxygenated carbon support to produce PtPdRhRuCe NPs that can catalyze ammonia oxidation (Fig. 17c).^288^ The precise control of impact parameters such as temperature, duration, and heating rate can effectively modulate the particle size, dispersion, and final HEA structure. At high temperatures, the catalytic activity of liquid metals drives rapid particle fission and fusion events, leading to homogeneous mixtures of multiple elements, facilitating kinetic control of thermodynamic mixing states and enabling the formation of crystalline solid-nanogranular solutions (Fig. 17d). Despite variable reduction potentials, multiple crystal structures, different atomic radii (1.24 to 1.44 Å), and altered melting temperatures (500 to 2000 K), Yao *et al.* successfully fabricated HEA NPs with eight dissimilar elements (Pt, Pd, Ni, Co, Fe, Au, Cu, and Sn) using the CTS method. Therefore, the CTS method provides an avenue for synthesizing a library of multicomponent HEA NPs with uniform elemental dispersion.

#### Fast moving bed pyrolysis

**Fig. 18:**
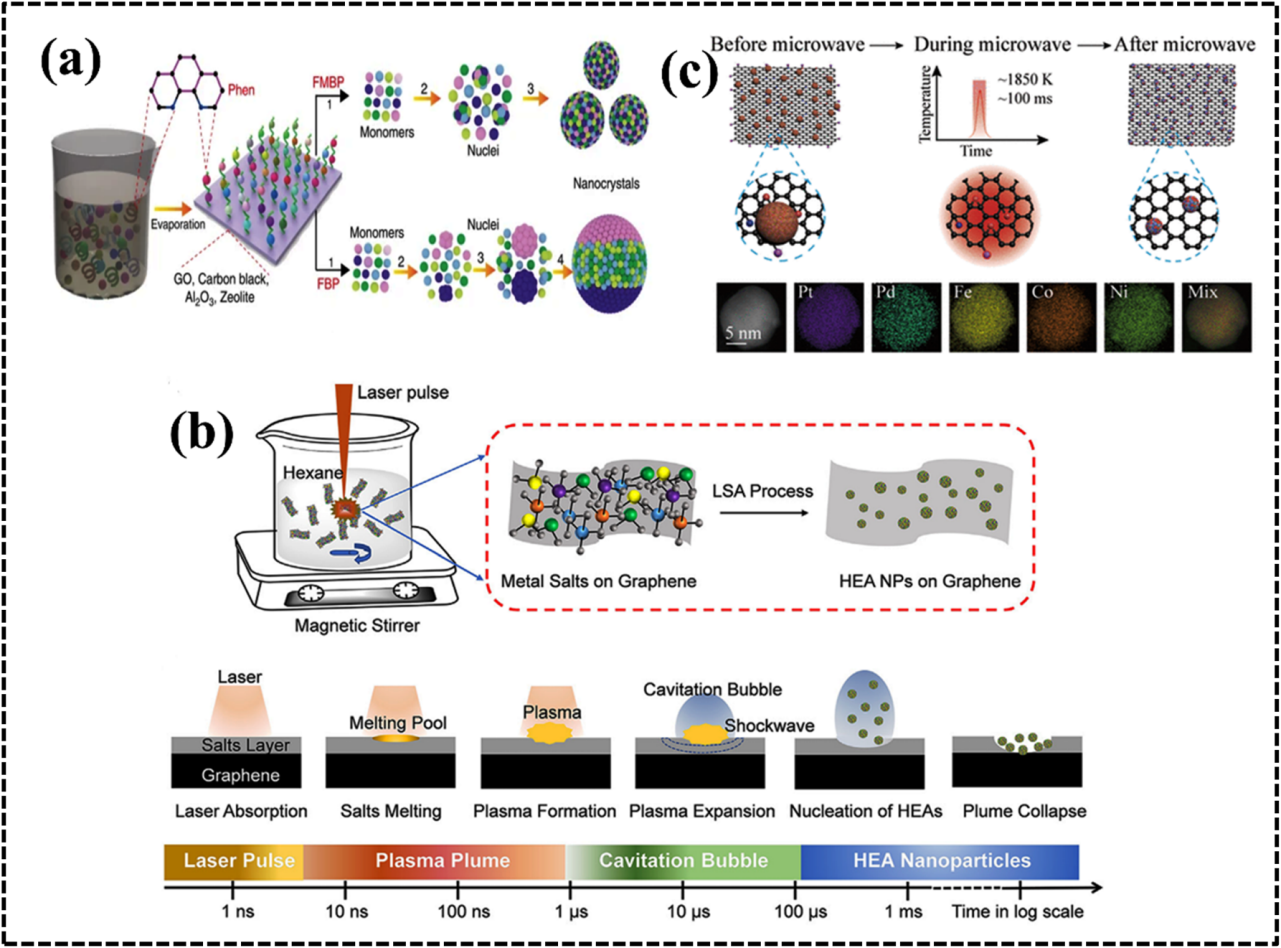
(a) Schematic diagram of the fast-moving bed pyrolysis experimental setup for synthesis of HEA nanoparticles and compression using fixed bed pyrolysis. Reproduced with permission.^191^ Copyright 2020, Spinger Nature. (b) Synthesis of HEA nanoparticles using the laser scanning ablation method. Reproduced with permission.^291^ Copyright 2023, Cell Press. (c) Schematic illustration of the formation of HEA nanoparticles on reduced graphene oxide *via* microwave heating. Reproduced with permission.^293^ Copyright 2021, American Chemical Society.

Fast moving bed pyrolysis (FMBP) involves the rapid heating of metal precursors to produce NPs, which are then annealed to form a nanostructured HEA. The FMBP process is similar to the CTS method, and the formation mechanism of a HEA is determined by the low free energy of the nucleation process at high reaction temperatures and rapid heating/cooling rates. The FMBP process is performed in three stages, which are the conversion of precursors to monomers, conversion of monomers to cores, and conversion of cores to nanocrystals. The reaction temperature and nucleation rate change the nuclear radius and free energy during the nucleation process and control the size and composition of HEA NPs. Lu *et al.* demonstrated an FMBP strategy to immobilize HEA NPs on granular supports with a narrow size distribution of 2 nm up to denary (MnCoNiCuRhPdSnIrPtAu) HEA NPs at 923 K (Fig. 18a).^191^ Interestingly, the FMBP method creates small and highly crystalline multicomponent HEAs with uniform distribution on different supports.^289^ However, note that the FMBP method requires specialized equipment to operate under high temperature and rapid heating conditions.

#### Laser scanning ablation technology

Laser scanning ablation (LSA) uses a focused laser to ablate target materials and form HEA NPs. By kinetically controlled laser synthesis, Wang *et al.* synthesized colloidal HEA NPs to achieve the target equimolar stoichiometry for CoCrFeMnNi HEA NPs from the liquid phase without using stabilizing ligands.^290^ Zou *et al.* synthesized the PtIrCuNiCr HEA using the LSA strategy with potential OER activity (Fig. 18b).^291^ This laser-based synthesis method is highly adaptable and can rapidly produce HEAs with different alloy compositions, target materials, and immobilized substrates, making it an excellent candidate for nanomaterial screening. Recently, Zou *et al.* developed a new type of LSA method using pulsed nanosecond lasers to obtain a series of HEA NPs at ambient temperature and pressure.^292^ This ultrafast process ensures that even thermodynamically incompatible metallic elements are fused together, overcoming the limitations imposed by conventional synthesis methods. A series of HEA NPs were prepared and supported on various substrates, including carbon substrates, non-carbon substrates such as Cu foam, and in certain cases HEA NPs even without a substrate, resulting in colloidal HEA NPs. This strategy was extended to the synthesis of several derivatives of HEAs such as amorphous HEO, HES, HEB, HEP, and HEN, confirming their versatility.

#### Microwave heating

Microwave heating has recently garnered attention as a potential alternative to traditional smelting methods for synthesizing HEAs. Hu *et al.* employed the microwave heating technique to synthesize PtPdFeCoNi HEA NPs over a carbon base with uniform particle size and homogeneous elemental distribution.^293^ In principle, the reduced graphene oxide nanosheets can sufficiently balance the functional group defects to efficiently absorb microwave radiation and simultaneously retain the superior thermal conductivity to rapidly transfer the induced localized heat. Moreover, a roll-to-roll method along with microwave heating provides a huge space to design various carbon-based materials as a background template. Moreover, the particle size is varied by varying the template (Fig. 18c).^293^ Nair *et al.* discovered that the microstructure of Al_*x*_CoCrFeNi HEA coatings transforms from FCC into BCC as a direct consequence of the Al content.^294^ This transformation is attributed to the influence of Al on the lattice constant and stacking fault energy of the alloy. Microwave-assisted techniques offer several advantages compared to traditional melting and solid-state synthesis methods, making them a promising strategy for developing HEA coatings with tailored microstructures and properties for various applications in materials science and engineering.

### Comparison between synthesis methods

The catalytic activity of the designed electrocatalyst is governed by the surface state, active sites, and electronic structure. By appropriately adjusting these parameters, possible electrode materials can be designed. The choice of elemental composition also plays an essential role in the performance, as active centers are present in the electrode material. Therefore, the choice of synthesis method should be based on the parameters that are required during HEA formation. With an appropriate synthesis route, stable and highly selective electrocatalysts can be produced. Above all, cost and productivity are relevant in the practical application of the synthesis route. Low cost and high productivity contribute to the mass synthesis of HEAs and meet the requirements for industrial applications. Table 7 lists a broad overview of the synthesis equipment, synthesis conditions, particle size, elemental distribution, synthesis time, and percentage of products to determine the most efficient electrode material for a particular electrochemical process. This review identifies electrocatalysts as electrode materials and describes the catalytic parameters determined from the basic parameters to be effective as industrial electrode materials The limitations of the various techniques are presented subsequently. The CTS synthesis requires a carbon support and the production volume is significantly low (milligram scale) in terms of industrial applications. If the productivity of HEAs using CTS technology can be significantly improved, then this synthesis method can be used for industry purposes. Although MA is a simple and cost-effective method, it cannot control the morphology and size of the HEA. Therefore, variations in composition cannot be optimized in this approach, which restricts the potential use of this method. The electrochemical approach requires improved product proportions, morphology, and compositional variations to be considered as an effective synthesis technique. The SD method generates high-temperature, high-pressure plasma that can control the microstructure of HEAs with satisfactory scalability and purity. Microwave heating techniques can control HEA size with short reaction times; however, the morphology of the HEA system is not controllable. Despite this, microwave heating approaches can achieve various compositional possibilities with the aid of harsh reaction conditions. However, controlling this technique is challenging; consequently, a uniform composition and microstructure is not easily achieved. LSA is a method that produces highly replicable and productive HEA NPs; however, its commercial applicability is limited by its unique ablation target and elemental immiscibility, along with issues such as process complexity, cost, and waste generation. The one-pot method using commercial metal precursors is an economical and easy synthesis procedure for HEAs. It allows different substrates to be applied by adding the corresponding precursor. However, the productivity of this method can be low and the size uniformity of HEAs must be improved. By adjusting the synthesis conditions, efficient HEA-based electrode materials with desired catalytic behavior can be fabricated. FMBP is a method for supporting HEA NPs on various substrates at different temperatures. It produces homogeneously dispersed HEAs with an active catalyst of tunable size, but requires high-temperature equipment and rapid heating rates, potentially limiting commercial applications. UAWC methods can be used to obtain HEAs that are supported on various substrates, depending on the electrochemical process requirements and system properties of the designed HEA. Dealloying methods are promising for commercial HEA production; however, they exhibit limitations in terms of scalability, precious metal content, and process control, hindering the fabrication of uniform compositions and microstructures. Therefore, the industrial application of HEA-based electrode materials currently requires some improvements in terms of elemental selection, compositional variety, electronic structure, and operational stability. In particular, as HEA-based electrode materials represent a burgeoning field that is evolving over time, it is imperative to address existing limitations in order to achieve desirable HEA-based electrode materials suitable for industrial applications.

### HEA characterization techniques

Unlocking the full potential of HEAs as electrode materials requires meticulous control of their elemental composition, extending beyond elemental selection to consider the type, distribution, and concentration of each constituent. Precise tailoring of these factors shapes the HEA's electronic and crystal structures, impacting its electrochemical performance. For example, varying element types introduces functionalities, while adjusting their distribution and concentration influences conductivity, stability, and active site availability. Understanding these relationships enables researchers to design HEA-based electrodes optimized for specific electrochemical applications.

Inductively coupled plasma mass spectrometry (ICP-MS), inductively coupled plasma absorption emission spectrometry (ICP-AES), and inductively coupled plasma optical emission spectrometry (ICP-OES) techniques provide information on the elemental composition of HEA-based electrode materials.^234,256^ Energy-dispersive X-ray analyses provide information on the homogenous distribution of the HEA. Electron microscopy techniques such as scanning electron microscopy, TEM, and high-angle annular dark-field STEM (HAADF-STEM) are commonly used to reveal the surface morphology, particle size, distribution, and exposed surface area of HEA materials.^234,239^ Atomic-scale HAADF-STEM and corresponding fast Fourier transform analysis can also help in identifying lattice planes and further refine the crystal structure of HEAs. X-ray photoelectron spectroscopy is another tool that can be used to estimate the chemical composition of HEAs.^40,239^ Brunauer–Emmett–Teller and Barrett–Joyner–Halenda methods were used to determine the specific surface area, pore volume, and pore size distribution of porous HEAs. XRD analysis determines the phase composition of HEAs from the peak position and intensity. Currently, the HEAs used as catalysts are primarily single-phase HEAs, while only a few two-phase HEAs have been proposed. XRD patterns of single-phase HEAs usually show only a series of diffraction patterns, such as FCC, BCC, and HCP, whereas HEAs with two or more phases show additional diffraction peaks in the XRD patterns.^39,257^ The oxidation state and local coordination environment of HEA-based electrode materials exhibit a significant influence on their catalytic activity. In particular, oxidation of surface elements and oxide film formation on the HEA surface have been reported to enhance catalytic activity and stability. The chemical state of HEAs can be further confirmed *via* X-ray absorption spectroscopy, X-ray absorption near edge structure (XANES), and extended X-ray absorption fine structure (EXAFS). In general, the XANES distribution indicates if the constituent elements of the HEA are in the metallic state and have chemical bonding properties. In the case of multi-element HEAs, more types of M–M bonding can be inferred, and the local coordination environment of HEAs is more complex than that of binary alloys. Several methods have been used to characterize HEAs, and the results obtained must be analyzed comprehensively to determine the HEA properties. Characterization methods are essential for ensuring the quality and suitability of HEAs for industrial applications. These methods provide important insights into the structure, properties, and performance of HEAs and enable researchers and engineers to tailor these materials to specific applications.

### Electrode fabrication and practical assessment of HEAs from an industrial perspective

The diversity of HEAs and their derivatives demonstrates numerous possibilities for designing electrocatalysts, ensuring that the different requirements of electrode materials are met. These materials are enhanced by their catalytic stability, efficiency, and selectivity. Their high entropy and sluggish diffusion effects enhance their long-term stability, making them suitable for practical applications. This section discusses potential applications of HEAs in electrocatalytic reactions such as the HER, HOR, ORR, OER, CO_2_RR, NRR, and AOR, focusing on their design principles and recent research progress.

#### Water splitting

Water electrolysis is a promising method to alleviate energy scarcity by generating green hydrogen from water (Table 8).^12,24,143^ The use of HEAs as cathode and anode electrode materials in commercial water electrolyzers can be an alternative approach to current materials. The electrocatalytic behavior of these materials varies in terms of electronic structure, substrate adsorption capacity, phase structure, and chemical behavior. The hydrolysis energy barrier can be reduced to optimize the reaction, and enhancing the H interactions can improve reaction kinetics.^16–20^ This process is multifaceted and involves multiple intermediates. To achieve high OER activity, multifunctional active sites can interact with different intermediates. Therefore, adjusting the adsorption positions of HO* and HOO* can improve the OER performance of the HEA system.^63^ The synergistic effects of alloys can be used to modify the local coordination environment and electronic structure, resulting in optimal intermediates with modified binding energies and improved OER activity. Thus, HEAs can be a suitable alternative for the current state-of-the-art electrocatalysts because of their atomic radii and lower formation heat, which can promote the successful formation of compositionally complex and stable HEAs, influencing their catalytic activity. The synergistic effect of Pt_18_Ni_26_Fe_15_Co_14_Cu_27_ HEA NPs includes multiple H-binding active sites, which increase the HER catalytic performance (11 mV overpotential at 10 mA cm^−2^ current density).^295^ The Co site exhibited the reduction of the e_g_–t_2g_ splitting effect and Ni with different valence states displayed a stable d-band center, thereby enhancing the electrocatalytic performance through superior electron transfer efficiency and low charge transfer resistance. Hence, the lattice distortion and structural relaxation of HEAs result in the catalytic stability of HEAs. The substitution of noble Pt metal with Pd is also an innovative approach for synthesizing HEA-based HER electrode materials.^296,297^ Based on theoretical calculations, the optimization of electronic structures based on the synergistic effect of all metals can be attributed to the presence of dominant electroactive sites.^298^ Dai *et al.* synthesized a series of noble-metal-based HEA NPs (PtAuPdRhRu, PtAuPdRh, and PtAuPd) using a simple UAWC method to achieve alkaline HER.^196^ The highest catalytic performance was observed for the highest compositional variety (PtAuPdRhRu) at a higher synthesis temperature. The superior electrocatalytic performance of HEA NPs was attributed to the strong synergistic effect within the metal atoms and high entropy of mixing at that composition.^299^ Therefore, tuning of the hydrogen binding energy (HBE) is an effective way to optimize the electrode material for the HER, particularly for applications in an acidic environment. For instance, Pt-free, HBE-optimized PdMoGaInNi nanosheets exhibited high HER activity with a low overpotential of 13 mV at 10 mA cm^−2^, outperforming commercial Pd/C and Pt/C catalysts.^298^ Lattice distortion and the sluggish diffusion effect of HEAs were observed to impact durability in a proton exchange membrane water electrolyzer system. Theoretical studies by Zhu *et al.* showed that the Ru site exhibited low Gibbs free energy of adsorbed atomic hydrogen in FeCoNiMnRu HEA NPs, which aided in constructing controllable multifunctional active sites on the surface of the HEA.^286^ The electron transition from low electronegative TM to noble metals (Pt, Ir, Pd, *etc*) modifies the electronic structure and active surface sites, and thereby the H adsorption phenomenon on the HEA improved. For example, the introduction of the transition metal in FCC type FeCoNiCuIr changed the electronic structure of Ir and optimized the HBE.^299^ Mao *et al.* observed abundant lattice distortions and defects in ultrathin HEA-PdPtRhIrCu metallene.^300^ DFT calculations could conclude that the optimized Pt electronic structure in the HEA promotes HER performance through the developed synergistic effect. In particular, the strong coupling effect and strong bonding arising from the interaction between the multi-metal components can facilitate the electron transfer over the surface.^300^

**Table 8:** HEA based electrode materials for the hydrogen evolution reaction

Electrode	Synthesis	Electrolyte	Performance *η* @ 10 mA cm^−2^, *b* = Tafel slope	Key points	Reference
FeCoNiCuMnN/CC-400	Hydrothermal	Alkaline	*η* = 184 mV, *b* = 113 mV dec^−1^	Superior corrosion and oxidation resistance	143
Co_0.6_(VMnNiZn)_0.4_PS_3_	Solid-state reaction	1 M KOH	*η* = 65.9 mV, *b* = 65.5 mV dec^−1^	Exposed S and Mn edge sites and basal P sites	155
Ni_20_Fe_20_Mo_10_Co_35_Cr_15_	Arc-melting	0.5 M H_2_SO_4_	*η* = 107 mV, *b* = 41 mV dec^−1^; *η* = 172 mV, *b* = 66 mV dec^−1^	TM active sites provide corrosion resistance	190
IrPdPtRhRu NPs	Polyol process	0.5 M H_2_SO_4_	*η* = 33 mV, *η* = 22 mV	HEA NPs contain different atomic arrangements with unique local density of states	192
AlNiCoIrMo	Dealloying	0.5 M H_2_SO_4_	*η* = 18.5 mV, *b* = 33 mV dec^−1^	Synergistic effect	197
Al_87_Ag_1_Au_1_Co_1_Cu_1_Fe_1_Ir_1_Mo_1_Ni_1_Pd_1_Pt_1_Rh_1_Ru_1_Ti_1_	Dealloying	0.5 M H_2_SO_4_	*η* = 32 mV, *b* = 30.1 mV dec^−1^	Synergistic effect and nanoporous structure	234
Nanoporous Ni_14_Co_14_Fe_14_Mo_6_Mn_52_	One-step dealloying	Alkaline	*η* = 150 mV @ 1000 mA cm^−2^	Synergetic effect of optimized hydrogen adsorption in the segregation area	271
Mn_70_Ni_7.5_Cu_7.5_Co_4.2_V_4.2_Fe_2_Mo_2_Pd_0.5_Pt_0.5_Au_0.5_Ru_0.5_Ir_0.5_	One-step dealloying	Alkaline	*η* = 21 mV, *b* = 21.5 mV dec^−1^	Nano-porous, distribution elements, and strong interaction of mixed-metal elements	272
NiCoFePtRh NPs	Co-reduction method	0.5 M H_2_SO_4_	*η* = 27 mV, *b* = 30.1 mV dec^−1^	Active sites, tunable electronic structures, and synergistic effect improve performance	278
FeCoNiMnRu/CNFs	Electrospinning	1.0 M KOH	*η* = 71 mV @ 100 mA cm^−2^, *b* = 67.4 mV dec^−1^	Co site facilitates H_2_O dissociation and Ru sites accelerate combination of H* to H_2_	285
PtIrCuNiCr HEA NPs	Laser scanning ablation	1 M KOH	*η* = 200 mV @ 100 mA cm^−2^	Strain enhances electrode activity	291
Pt_18_Ni_26_Fe_15_Co_14_Cu_27_/C	One-pot oil-phase synthesis	1 M KOH	*η* = 11 mV, *b* = 30 mV dec^−1^	Multiple active sites are effective for intermediate adsorption	295
PdMoGaInNi	Wet chemical method	0.5 M H_2_SO_4_	*η* = 13 mV, *b* = 108.9 mV dec^−1^	Pd–Mo–Ga promising HER catalyst	298
FeCoNiCuIr	Hydrothermal	0.1 M HClO_4_	*η* = 71 mV, *b* = 41.7 mV dec^−1^	Incorporation of TM modifies the electronic structure	299
PdPtRhIrCu	Hydrothermal	1 M KOH	*η* = 15 mV, *b* = 37 mV dec^−1^	Coupling and bonding interactions facilitate electron transfer	300
NiFeCoCuTi	Arc melting	1 M KOH	*η* = 209 mV @ 2 A cm^−2^, *b* = 43 mV dec^−1^	Ni skeleton enhances electron/mass transfer	301
WMoVNbCeB	Thermal reduction method	1 M KOH	*η* = 117 mV @ 50 mA cm^−2^, *b* = 111 mV dec^−1^	Combination of electron orbit of the outer layer of the element promotes the HER	303
ZnNiCoIrMn	Sol–gel method	0.1 M HlO_4_	*η* = 50 mV, *b* = 30.6 mV dec^−1^	Mn incorporation tailors electrode performance	309

**Fig. 19:**
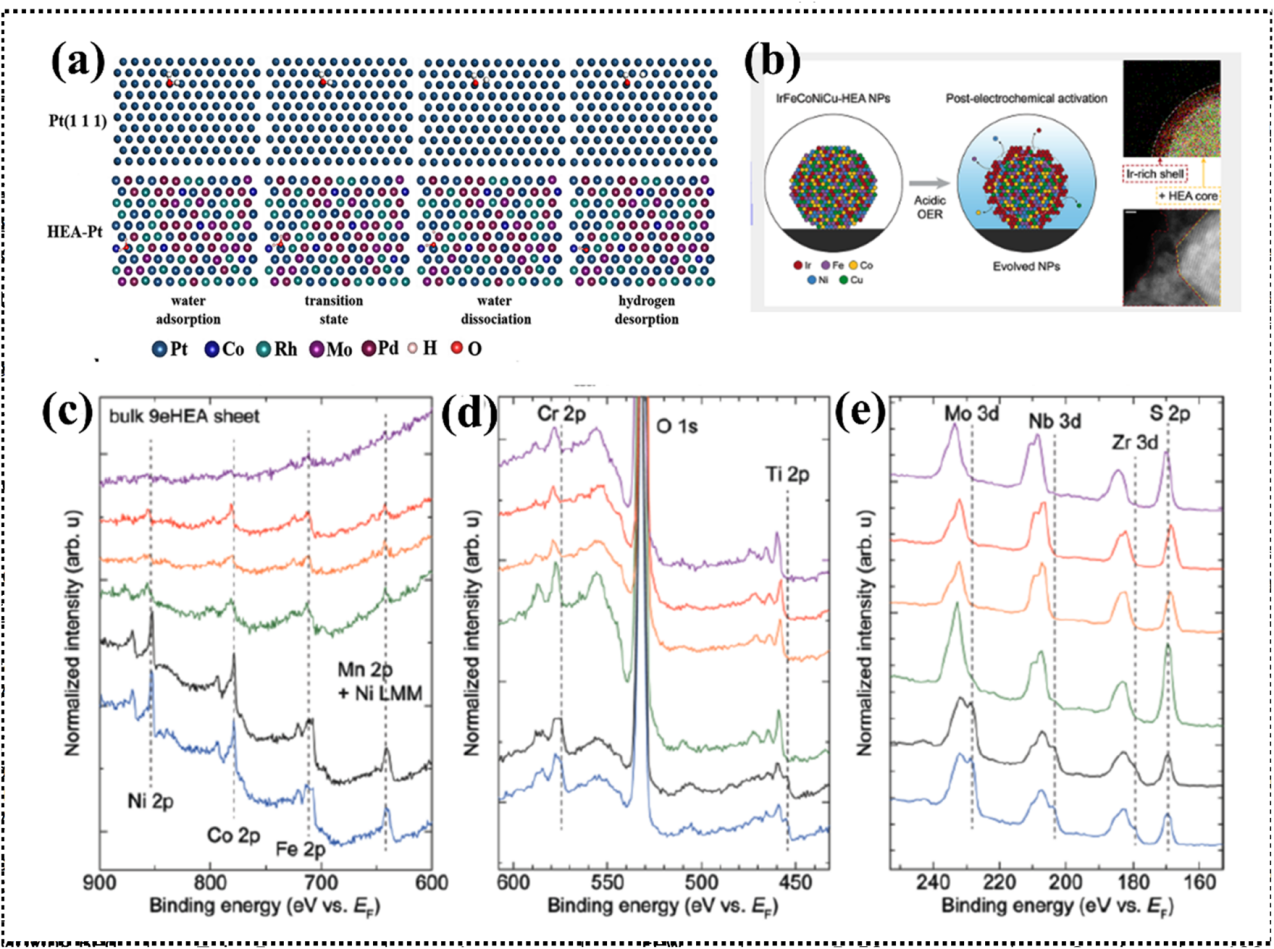
(a) H adsorption over the Pt (111) plane. Reproduced with permission.^302^ Copyright 2023, Elsevier Ltd. (b) Structural evolution of HEA nanoparticles in terms of the oxygen evolution reaction. Reproduced with permission.^304^ Copyright 2023, American Chemical Society. (c–e) Electrochemical X-ray photoelectron spectroscopy (XPS) data in the survey mode for HEAs. Reproduced with permission.^306^ Copyright 2023, Wiley VCH.

The electroactive sites in a self-supported hierarchical nanoporous high-entropy NiFeCoCuTi alloy facilitate electron transfer and mass transportation processes during an alkaline HER process. The multicomponent NiFeCoCuTi alloy serves as a multisite electroactive center that accelerates water dissociation and mediates the combination of H* into H_2_.^301^ The enhanced HER activity of PtCoMoPdRh/PtNiMoPdRh HEA nanoflowers is attributed to the multiple active sites in HEAs and the strain effect induced by a unique structure that reduces the water dissociation energy barriers (Fig. 19a).^302^ The water dissociation activation energy of Pt_16_ in the Pt_35_Co_5_Mo_10_Pd_25_Rh_25_ (111) plane decreases from 0.57 to 0.16 eV as compared to that in the pure Pt (111) plane, indicating a chemical environment that alters the electronic structure and accelerates the Volmer step. The Pt (111) plane is considered to be the main active site for H adsorption, whereas Rh, Mo, Pd, and Co regulate the electronic and chemical environments, which benefits the HER process. Recently, Dong *et al.* developed three different rare-earth transition-metal HEB structures (WMoVNbCeB (HEB-Ce), WMoVNbSmB, and WMoVNbLaB) and determined that the temperature is a function of the d-band structure, thereby achieving a better catalytic performance at a higher synthesis temperature.^303^ In HEB-Ce, the d-band center of V, Nb, and Ce moved deep down the conduction band and deviated from the Fermi level, which strengthened the antibonding impact and promoted H_2_O decomposition. The choice of metal and HBE for the developed electrode materials can judiciously replace commercially used HER electrode materials in PEMWE, AEM, AEMWE, and SOWE systems. In general, HEAs are more efficient and stable than HEC electrodes as HER electrodes, possibly due to the potential mechanistic pathway of the HER process. Therefore, HER-active transition and noble metals can be used to design pristine HEAs for cathode materials for water electrolyzers. Moreover, the choice of electrode also varies depending on the design of the electrolyzer, which should be considered before designing the electrode.

OER electrocatalysts play a crucial role in efficient water electrolyzers. Effective OER electrode materials require multifunctional active sites that interact with intermediates such as HO* and HOO* to facilitate the four-electron-transfer water oxidation reaction.^112,144,193,206^ HEAs exhibit significantly modified local coordination environments and electronic structures owing to the synergistic effects of compositionally complex multimetallic moieties, making them promising OER candidates. Noble metals such as Ir and Ru and their oxides, as well as transition-metal-based metal oxides/hydroxides, demonstrate remarkable OER activity.^206,212^ Designing active electrode materials for the OER involves incorporating active materials that can effectively regulate the electronic environment of HEA-based materials to achieve enhanced electrochemical performance (Table 9). The structural reconstruction and evolution of active intermediates in IrFeCoNiCu HEA NPs on a carbon-paper substrate led to better OER performance when compared to that of the mono-metallic Ir counterpart owing to the presence of the synergistic effect and modified d-band structure. After electrochemical activation, delamination of the encapsulated carbon layers and the dissolution of the constituent 3d metal atoms (Fe, Co, Ni, and Cu) from the surface of the NP occurred, leaving an Ir-rich shell layer, while the core maintained the homogeneous single-phase HEA structure (Fig. 19b).^304^ This exposed Ir-rich surface provides a high number of OER active sites and concurrently improves the electrode performance in the water oxidation reaction.

**Table 9:** HEA-based electrode materials for the oxygen evolution reaction

Electrode	Synthesis	Electrolyte	Performance *η* @ 10 mA cm^−2^, *b* = Tafel slope	Key points	Reference
FeCoNiCuCrNps	Mechanical ball milling	1 M NaOH	*η* = 330 mV, *b* = 80 mV dec^−1^	OER activity optimized by tuning compositions	63
CoFeNiCrV-LH	Electrochemical method	1 M KOH	*η* = 232 mV, *b* = 38.6 mV dec^−1^	Defect-rich, low-crystalline active sites originating from entropy-driven lattice distortions and oxygen vacancies	112
La(CrMnFeCo_2_Ni)O_3_	Mechanochemical method	1 M KOH	*η* = 325 mV, *b* = 51.2 mV dec^−1^	Unique structure forms defects and tunes the structure	121
(Co, Cu, Fe, Mn, Ni)_3_O_4_/MWCNT	Solvothermal synthesis	1 M KOH	*η* = 350 mV, *b* = 59.5 mV dec^−1^	—	132
Cu_0.5_Fe_0.5_NNi_2_Co_0.5_Fe_0.5_	Chemical solution deposition (CSD)	1 M KOH	*η* = 370, *b* = 55 mV dec^−1^	Anti-perovskite metal nitrides exclusively for the OER	144
K_0.8_Na_0.2_(MgMnFeCoNi)F_3_	Hydrothermal method	Alkaline	*η* = 314, *b* = 55 mV dec^−1^	High-entropy perovskite fluorides effective for the OER	145
(CoFeNiMnCu)S_2_ NPs	Solvothermal method	1 M KOH	*η* = 284 mV, *b* = 57 mV dec^−1^	Pyrite structure contains exposed active sites	152
MnFeCoNiCu HEAN MOFs	Solvothermal method	1 M KOH	*η* = 263 mV, *b* = 43 mV dec^−1^	Single-phase FCC HEA nanoparticles with a size of sub 5 nm	103
AlNiCoIrMo	Dealloying	0.5 M H_2_SO_4_	*η* = 233 mV, *b* = 55.2 mV dec^−1^	Mo enhances OER performance	197
CoCuFeMoOOH	Solvothermal method and electrochemical reconstruction	1 M KOH	*η* = 119 mV, *b* = 48.8 mV dec^−1^	Co-based (oxy)hydroxide HEA with M−O covalency enhances the OER	219
Al_89_Ag_1_Au_1_Co_1_Cu_1_Fe_1_Ir_1_Ni_1_Pd_1_Pt_1_Rh_1_Ru_1_	Dealloying	0.5 M H_2_SO_4_	*η* = 258 mV, *b* = 84.2 mV dec^−1^	Synergistic effect and nanoporous structure	234
Nanoporous Ni_14_Co_14_Fe_14_Mo_6_Mn_52_	One-step dealloying	1 M KOH	*η* = 350 mV, *b* = 43 mV dec^−1^	Porous structure increases electrochemically active area and exposes electrochemically active sites	271
Mn_70_Ni_7.5_Cu_7.5_Co_4.2_V_4.2_Fe_2_Mo_2_Pd_0.5_Pt_0.5_Au_0.5_Ru_0.5_Ir_0.5_	One-step dealloying	Alkaline	*η* = 205 mV, *b* = 74.2 mV dec^−1^	Nanoporous structure, distribution of elements, and strong interaction of mixed-metal elements	272
LiMoFeCoNi HEH	Coprecipitation method	1 M KOH	*η* = 187 mV, *b* = 82 mV dec^−1^	Lattice distortions in HEHs lead to superior performance to that in LEHs	276
FeCoNiMnRu/CNFs	Electrospinning	1.0 M KOH	*η* = 241 mV	Multiple metals serving as active centers	285
PtIrCuNiCr HEA NPs	Laser scanning ablation	1 M KOH	*η* = 176 mV	Strain enhances electrode activity	291
CoNiFeMnCr	—	0.5 M H_2_SO_4_	*η* = 528 mV, *b* = 150 mV dec^−1^	Self-selection and reconstruction improve activity and stability	306
Ni/CoNiFeMoCr	Polymer/metal precursor spray synthesis	1 M KOH	*η* = 350 mV @ 50 mA cm^−2^	Multiple active sites	307
NiMnFeCrCu	Mechanical milling	1 M KOH	*η* = 310 mV, *b* = 83 mV dec^−1^	Contains FCC phases and a BCC phase	308
ZnNiCoIrMn	Sol–gel method	0.1 M HlO_4_	*η* = 237 mV, *b* = 46 mV dec^−1^	Mn incorporation tailors electrode performance	309

Ru-based ultrasmall FeCoNiIrRu HEA NPs on electrospun CNFs were synthesized, and experimental analyses demonstrated that metal combinations and crystallization temperatures could tune the OER activity. An *in situ* characterization approach showed that phase transition in the HEA enhances OER efficiency, and thereby achieving a certain specific phase can reduce the required potential for the OER. Theoretical calculations demonstrated that electron density transport occurs from a transition-metal-based low-electronegative element (Fe, Co, and Ni) to a high-electronegative noble metal (Ir and Ru) on FeCoNiIrRu HEA NPs, which promotes the OOH* to O_2_ transformation.^305^ The combination of passivation elements and catalytically active elements has played a significant role in designing corrosion resistance and catalytically active electrode materials. Considering this, Ito *et. al.* systematically designed a nine-element HEA by combining the corrosion-resistant Ti, Zr, Nb, and Mo elements with catalytically active Cr, Co, Ni, Mn, and Fe, which exhibited optimal corrosion resistance and catalytic abilities in terms of the OER.^306^ The self-selection and self-construction processes control the OER catalytic activity and stability of the HEA system (Fig. 19c–e). The corrosion-resistant multi-element alloys with high catalytic activity are crucial for replacing noble-metal catalysts not only in proton exchange membrane-type water electrolyzers but also in other types of electrolyzers, electrolytic systems, and battery systems. A straight forward and scalable polymer/metal precursor spraying synthesis technique was developed to produce different multi-metal catalyst powders for the OER. Operando Raman spectroscopy results indicated that the formation of multiple M−O active sites on CoNiFeMoCr improved the OER performance.^307^

In this regard, HEAs containing refractory elements can be an alternative choice for use under harsh electrolyte conditions in water electrolyzers. Specifically, they can be utilized in SOWE systems that operate at high operating temperatures. An improved electrocatalytic property could be attributed to the formation of electrochemically active orthorhombic species with active sites on the NiMnFeCrCu HEA.^308^ Schuhmann *et al.* investigated the optimized overall water splitting performance of the CoNiFeMoCr/Ni_f_ HEA as an anode and cathode electrode in a three-electrode configuration flow-through cell. They determined that catalyst loading influenced the electrode performance. The designed HEA electrode materials were successfully applied as cathode and anode materials in a membrane electrode assembly comprising 1 M KOH, demonstrating their potential for industrial use.^307^ In general, HECs are more effective for the oxygen evolution reaction (OER) compared to HEAs due to the possibility of easy formation of the pre-catalyst from the HEC during the OER. Moreover, the choice of metals and anions, and the morphology of the electrode material have a pronounced effect on the electrode performance in the respective electrolyzers.

A proton exchange membrane electrolysis is considered as the most advanced water electrolyzer for hydrogen economy. In this regard, Song *et al.* synthesized Ir-based electrocatalysts using a ZnNiCoIrX HEA platform containing two elements (X: Fe and Mn) that implied emerging faradaic efficiency.^309^ Interestingly, the incorporation of Mn in a HEA adjusts the electronic structure of the Ir sites, moving the d-band center away from the Fermi level and weakening the adsorption energy. Compositional engineering adjusts the electronic structure of the active Ir sites, thereby altering the aggregation and adsorption energies and inhibiting elemental dissolution. Choi *et al.* developed a photothermal method that satisfies the atmospheric pressure, large-area, remote-process, and material selection requirements. They designed and tested polymeric HEA NPs (PtIrFeNiCoCe) with high activity and stability in water splitting over 5000 cycles. This approach is efficient in mass and time productivity and compatible with the atmosphere, achieving the homogeneous synthesis of multi-metal catalysts. These advantages suggest the industrial applicability of HEA-based electrode materials for water electrolysis in different electrolyzer systems (Table 10).^310^ Moreover, before designing the cathode and anode electrodes, the operating conditions, electrolytic medium, mechanism, and setup should be considered.

**Table 10:** HEA-based electrode materials for water splitting

Electrode	Synthesis	Electrolyte	Performance @ 10 mA cm^−2^	Key points	Reference
(CoCrFeMnNi)P	Calcination	1 M KOH	1.78 V @ 100 mA cm^−2^	Synergistic effect improves electrode activity	153
Al_89_Ag_1_Au_1_Co_1_Cu_1_Fe_1_Ir_1_Ni_1_Pd_1_Pt_1_Rh‖Al_87_Ag_1_Au_1_Co_1_Cu_1_Fe_1_Ir_1_Mo_1_Ni_1_Pd_1_Pt_1_Rh_1_Ru_1_Ti_1_	Dealloying	0.5 M H_2_SO_4_	1.53 V	Synergistic effect	234
AlNiCoIrMo	Dealloying	0.5 M H_2_SO_4_	1.4 V	—	197
Nanoporous Ni_14_Co_14_Fe_14_Mo_6_Mn_52_	One-step dealloying	1 M KOH	1.48 V	Porous structure increases electrochemically active area and exposes electrochemically active sites	271
PtIrCuNiCr HEA NPs	Laser scanning ablation	1 M KOH	1.42 V	Strain enhances electrode activity	291

#### Oxygen reduction reaction

The ORR is a crucial electrochemical reaction in fuel cells and metal–air batteries. The four-electron reduction of oxygen to water can be achieved through direct or indirect mechanisms proceeding *via* multiple intermediates (O*, OH*, HOO*, and O_2_^2−^). The ORR is a very noteworthy reaction in fuel cell design. One major challenge is to be able to prepare highly selective catalysts leading only to the four-electron reduction of O_2_ to water and thus minimizing the two-electron reduction to hydrogen peroxide. Pt-based materials are the best catalysts for this pathway; however, they are expensive and rare. The indirect four-electron pathway involves the reduction of oxygen to hydrogen peroxide in a two-electron step; however, it is less efficient because of the production of a corrosive byproduct. The indirect two-electron pathway has the least kinetic difficulty but the lowest efficiency. The choice of ORR pathway depends on the specific application, with the direct four-electron pathway preferred in fuel cells (H_2_/O_2_ or H_2_/Air) and considerations such as cost and durability being crucial in metal–air batteries. Pure Pt-based catalysts may have strong interactions with oxygen-containing materials, limiting the number of available O_2_ adsorption sites and catalytic activity. The addition of nonprecious metals to Pt alloys can promote O_2_ adsorption and dissociation, leading to O–O bond cleavage. High-energy anode catalysts are rich in surface sites with atomic arrangements, allowing a nearly continuous distribution of adsorption energy, leading to superior catalytic performance in the ORR.

**Fig. 20:**
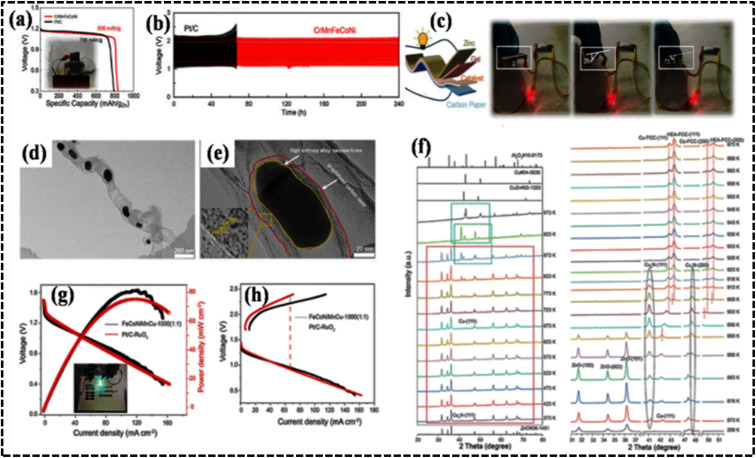
(a) Specific capacity comparisons between a CrMnFeCoNi- and Pt/C-based Zn–air battery (ZAB). Photograph of a red LED screen powered by a CrMnFeCoNi-based ZAB is also shown. (b) Galvanostatic discharge–charge curves. (c) Solid and flexible ZAB schematic diagram. Reproduced with permission.^311^ Copyright 2023, Elsevier Ltd. (d and e) TEM image of a HEA, (f) *in situ* Raman spectra of a HEA system, and (g and h) ORR performance of a HEA. Reproduced with permission.^315^ Copyright 2023, The Royal Chemical Society.

The Sabatier principle is a useful guideline for designing ORR catalysts with optimal intermediate adsorption (Table 11). Along with the OER performance, the CrMnFeCoNi HEA demonstrated promising ORR activity with a half-cell potential of 0.78 V and an onset potential of 0.88 V, which are comparable to those of a commercial Pt/C catalyst (Fig. 20a).^311^ The potential gap (*E*_gap_) between the OER overpotential and ORR half-cell potential of CrMnFeCoNi is merely 0.734 V. Considering the ORR efficiency, Zn–air batteries (ZABs) were assembled using the CrMnFeCoNi HEA as the air cathode and zinc foil as the anode. These assembled cells exhibited an open-circuit voltage of 1.489 V, which was 90% of its theoretical limit (1.66 V), a peak power density of 116.5 mW cm^−2^, and a specific capacity of 836 mA h g^−1^ and remained stable for over 10 days of continuous cycling, corresponding to 720 cycles at 8 mA cm^−2^, and 16.6 days of continuous cycling, corresponding to 1200 cycles at 5 mA cm^−2^ (Fig. 20a and b). A flexible ZAB was assembled using a polished Zn plate (anode) and hydrophilic carbon paper coated with the CrMnFeCoNi HEA as the cathode, with polyvinyl alcohol-KOH-H_2_O gel as the electrolyte. CrMnFeCoNi-based ZABs could power a red LED while bent at various angles, demonstrating their flexibility during operation (Fig. 20c).

**Table 11:** HEA-based electrode materials for the oxygen reduction reaction

Electrode	Synthesis	Electrolyte	Performance	Key points	Reference
np-PtRuCuOsIr	Chemical dealloying	0.1 M HClO_4_	*E*_1/2_ = 0.9 V, Δ*E* = 36 mV	1.8- and 3.8-times mass and specific activity than those of Pt/C	319
Mn_70_Ni_7.5_Cu_7.5_Co_4.2_V_4.2_Fe_2_Mo_2_Pd_0.5_Pt_0.5_Au_0.5_Ru_0.5_Ir_0.5_	One-step dealloying	Alkaline	*E*_1/2_ = 0.875 V, Δ*E* = 15 mV	Nanoporous structure, distribution of elements, and strong interaction of mixed-metal elements	272
AlNiCoRuMo	Dealloying	1 M KOH	*E*_1/2_ = 0.9 V, Δ*E* = 50 mV, electron transfer = 3.9	Ru and Mo enhance activity	273
PdCuPtNiCo HEAs/C	Seed-mediated co-reduction, heating, and quenching	0.1 M KOH	*E*_onset_ = 1.06 V, *E*_1/2_ = 0.83 V	Atomic-level mixing creates high interfacial strain	279
PtFeCoNiCu	Reduction method	0.1 M HClO_4_	*E*_1/2_ = 0.88 V, Δ*E* = 50 mV, *b* = 68 mV dec^−1^, electron transfer = 4	d-band center of Pt monitors the ORR	312
rGO@OHEA-mNC nanosheets	Wet chemical	1 M KOH	*E*_1/2_ = 0.9 V	HEA phase increases mass transfer and electron conductivity	313
PtPdFeCoNi	Wet chemical	0.1 M HClO_4_	*E*_1/2_ = 0.920 V, Δ*E* = 50 mV	Core effect improves the ORR	314
FeCoNiMnCu-1000 (1 : 1)	Solid-state thermal reaction method	0.1 M KOH	*E*_onset_ = 0.92 V, *E*_1/2_ = 0.78 V	Nanoparticles increase activity	315

In another report PtFeCoNiCu HEA NPs exhibited ORR catalytic mass activity that was 15.8 times higher than that of a Pt/C catalyst.^312^ DFT results confirmed that multi-element alloying lowered the activation barriers for O–O bond cleavage and changed the binding energy of OOH* intermediates to improve the reaction rate by combining metals with different oxygen affinities. This approach may be promising in the development of ORR catalysts for industrial application. A structurally ordered HEA (OHEA) NP was synthesized on a novel 2D nitrogen-rich mesoporous carbon sandwich framework (OHEA-mNC) *via* a combination of ligand-assisted interfacial assembly and NH_3_ annealing.^313^ XRD spectral analysis and atomic-resolution chemical mapping *via* TEM confirmed that the HEA was formed in a chemically ordered L1_2_ phase, exhibiting superior activity with a large half-wave potential (0.90 eV). The Pd and Cu atoms promote electron transfer on the HEA surfaces, while Co sites alleviate the e_g_–t_2g_ splitting effect, enhancing the electron transfer process. Both experimental and theoretical findings indicate that the superior performance stems from the well-designed spatial structure and presence of the stable chemically ordered HEA phase, facilitating fast mass transfer and modulating active sites for adsorbing reaction species and electron transfer during the ORR process. The electrochemical kinetics and stability of Pt-based catalysts are still far from meeting the requirements in practical applications, although progress has been made in this respect. HEA NPs of PtPdFeCoNi were fabricated using a high-temperature injection method by Yu *et al.*^314^ which exhibited superior catalytic activity and stability for the ORR owing to the high-entropy, lattice distortion, and slow-diffusion effects of the HEA. Therefore, it can be argued that increasing the minimum concentration of the noble metal enhances the mass transport process, thereby resulting in an enhancement of peak power density.

However, only transition metal-based HEA FeCoNiMnCu NPs encapsulated in N-doped graphitized hollow carbon tubes potentially prevent alloy particle aggregation during the ORR (Fig. 20d and e).^315^ The *in situ* XRD technique provided insights into the evolution of HEAs, identifying their formation point at approximately 908 K during the carbonization process (Fig. 20f). The catalyst exhibited superior catalytic activity, demonstrating ideal ORR performance in an alkaline solution. This high performance can be attributed to the synergistic effect of HEAs, high specific surface area, and graphite-encapsulated hollow carbon layer structure. The optimized catalyst demonstrated significant stability due to the carbonaceous scaffolds, outperforming commercial 20 wt% Pt/C–RuO_2_ in an assembled ZAB (Fig. 20f and h). A peak power density of 81 mW cm^−2^ and lower voltage gap between charge and discharge diagrams suggests the commercial applicability of the HEA in the ORR. The most critical challenge with non-noble metal-based electrode materials often lies in their lower mass transport efficiency and durability at the cathode in commercial fuel cells. Furthermore, while the highest activities based on rotating disk electrode (RDE) evaluations have been observed with shape-controlled Pt alloys and core–shell structures, whether these highly active ORR electrocatalysts can demonstrate the same enhancement factor in real fuel cells remains an open question. Significant efforts are needed to demonstrate their feasibility in fuel cell applications, ensuring desired performance and durability. Previous discussions have suggested that HEAs, comprising transition metals or a combination of transition metals and noble metals, with promising morphological and electronic properties, have the potential to replace Pt dominance in fuel cells commercially.

#### CO_2_ reduction reaction

The conversion of CO_2_ into value-added products (carbon monoxide (−0.52 V), formic acid (−0.43 V), methanol (−0.39 V), ethane (−0.34 V), ethanol (−0.33 V), propanol (−0.32 V), and methane (−0.25 V); all potentials are in the SHE scale at pH 7) is an emerging topic because of its potential to meet renewable energy needs and alleviate environmental problems caused by CO_2_ emissions. Among the various reduced products, the conversion to C2 and C3 products is complex because it involves multiple proton and electron transfer processes.^4,16,18^ Particularly, as the HER and CO_2_RR are competitive reactions, the activity, selectivity and stability of the designed catalyst must be improved to obtain the desired CO_2_ reduction products. Therefore, CO_2_ electroreduction can also be regarded as a convenient way to store the above renewable energies in chemical forms.

**Fig. 21:**
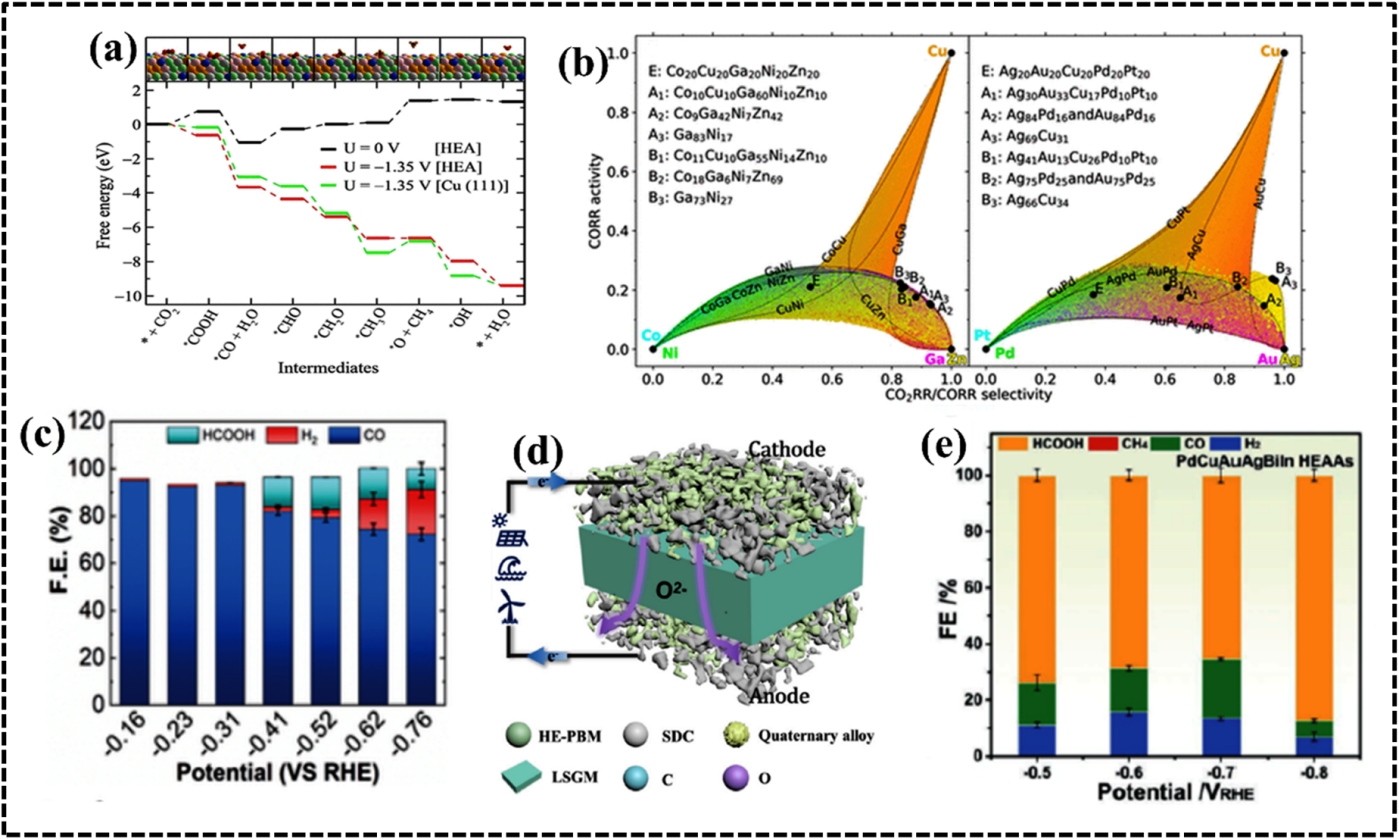
(a) Free energy diagram of the CO_2_ reduction reaction (CO_2_RR) on the HEA surface. Reproduced with permission.^189^ Copyright 2020, American Chemical Society. (b) Activity and selectivity plot of theoretically designed HEAs. Reproduced with permission.^227^ Copyright 2019, Wiley VCH. (c) Faradaic efficiency of the designed HEA for the CO_2_RR. Reproduced with permission.^151^ Copyright 2021, Wiley VCH. (d) Schematic CO_2_ electrolysis procedure. Reproduced with permission.^317^ Copyright 2023, Elsevier B.V. (e) Faradaic efficiency of the HEA for CO_2_ reduction. Reproduced with permission.^318^ Copyright 2023, Wiley VCH.

The electrocatalytic CO_2_RR is a multistep process that involves a number of electron and proton transfer reactions.^151,189,226^ However, high potential and low selectivity often hinder the reaction, leading to a limited reduction product yield. Multiple active sites are essential for facilitating the sequential coupling steps of the electrocatalytic reduction of CO_2_ that can convert simple molecules into more complex chemicals. The complexity of CO_2_ reduction arises from the large number of surface–bound reaction intermediates involved, and a variety of reduction products can be generated. As multi-electron reduction reactions require extensive proton and electron transfers through a variety of intermediates, alloys of different metal elements and compositions can significantly affect the types of products that are formed. Because different active sites promote different reactions, further study is required to fully understand the influence of alloys on the CO_2_RR, especially multi-element alloys (Table 12). Theoretical and experimental understanding suggests that most of the effective catalysts contain Cu or Ag owing to their effective interaction with the intermediate products. Nellaiappan *et al.* used a cast-cum-cryomilling process to synthesis the nanocrystalline equiatomic AuAgPtPdCu HEA for the efficient electrochemical reduction of CO_2_ to hydrocarbon (Fig. 21a).^189^ Although five elements were used, the electrocatalytic activity was primarily associated with the presence of redox-active Cu metal (Cu^2+^/Cu^0^), and other metals only provided a synergistic effect. The first-principles-based DFT analysis showed the reversal in adsorption trends for two out of the total eight intermediates of *OCH_3_ and *O on Cu (111) and HEA surfaces and also confirmed that the conversion of CO_2_ to gaseous products was low. In a similar approach, Rossmeisl *et al.* theoretically screened two HEA systems, CoCuGaNiZn and AgAuCuPdPt for the CO_2_RR.^227^ Their studies again confirmed that the Cu (111) plane of the FCC HEAs of CoCuGaNiZn and AgAuCuPdPt favored the selective reduction of CO_2_ to highly reduced products. They also proposed that the selectivity of the CO_2_RR/CO reduction reaction (CORR) and activity of the CORR are expected to change as the HEA catalysts vary in composition (Fig. 21b). Hence, the conversion efficiency is highly influenced by the specific metallic composition, as well as its variations. This underscores the ability of a model to predict promising catalyst candidates even without prior knowledge of their catalytic properties or the composition of disordered alloys for optimal catalytic performance.

**Table 12:** HEA-based electrode materials for the CO_2_ reduction reaction

Electrode	Synthesis	Electrolyte	Performance & key points	Reference
(MoWVNbTa)S_2_	Chemical vapor deposition method	Aq. 1 M KOH: 1 M choline chloride	Current density: −353, *η*: −0.76 V *vs.* RHE (631 mV), TOF: 209 885 h^−1^, CO Fe: 72%	151
AuAgPdPtCu NP	Cryomilling	0.5 M K_2_SO_4_	CH_4_: 38.2%; C_2_H_4_: 29.5%; CO: 4.9%; H_2_: 27.5%; potential: −0.3 V RHE	189
Pr_0.5_Ba_0.5_Mn_0.2_Fe_0.2_Co_0.2_Ni_0.2_Cu_0.2_O_3−*δ*_	Sol–gel method	—	CO Fe: 95%	317
PdCuAuAGBiIn HEA aerogel	Freeze–thaw method	0.5 M KHCO_3_	C1 Fe: 100% at −0.7 to −1.1 *V*_RHE_; HCOOH FE: 87.5%	318

Roy *et al.* conducted HT screening of HEA-based (Cu, Co, Ni, Zn, and Sn) catalysts through ML for CO_2_ hydrogenation to methanol.^226^ The designed CuCoNiZnSn-catalyst exhibited satisfactory performance in all possible combinations, where different elemental, compositional, and surface microstructural features were used as input parameters. Several theoretical studies have successfully designed HEAs for the CO_2_RR; however, these HEAs have not yet been experimentally verified. An extensive experimental study should be performed to achieve an effective HEA design, so that the HEAs can be used as industrial electrode materials for the CO_2_RR.^316^ Cavin *et al.* synthesized and tested five high-entropy 2D transition-metal dichalcogenide alloys containing four or five transition metals, and identified that the alloy with the highest configurational entropy, that is, (MoWVNbTa)S_2_, exhibited promising performance for CO_2_ electroreduction (Fig. 21c).^151^ Zhang *et al.* demonstrated that high-entropy perovskite oxide Pr_0.5_Ba_0.5_Mn_0.2_Fe_0.2_Co_0.2_Ni_0.2_Cu_0.2_O_3−*δ*_ effectively catalyzes CO_2_ electrolysis in SOECs. The *in situ* formation of Fe–Co–Ni–Cu quaternary alloy nanocatalysts exhibited enhanced catalytic activity for the CO_2_RR, as evidenced by the significantly lower polarizing resistance and higher electrolytic current density (Fig. 21d).^317^ Li *et al.* developed highly active and durable CO_2_RR catalysts by fabricating PdCuAgBiIn HEA aerogels (HEAAs) which demonstrated superior CO_2_RR activity for selectively producing HCOOH over a wide potential range. In a flow cell device with 0.5 M KHCO_3_, the HEAAs achieved a current density of nearly 200 mA cm^−2^ and a faradaic efficiency of HCOOH of 87%.^318^ This remarkable performance is attributed to the strong interactions between different metals and surface unsaturated sites in the HEAAs, which optimize the electronic structures and HCOO* interfacial adsorption, suppressing CO poisoning and competitive H_2_ production to favor HCOOH formation (Fig. 21e).

The literature survey highlights the extensive use of metal species such as Cu, Ag, Au, and Sn as catalysts for CO_2_ electroreduction. To facilitate electroreduction, it is imperative that the charged intermediate CO_2_˙^−^ actively participates, necessitating the development of new electrodes for better stabilization of this molecule. Metals exhibit differing tendencies to temporarily bind intermediates and products to the electrocatalyst surface, leading to their classification into three classes. The first class comprises metals such as Cu, where the most stable bonds are formed between the intermediate and the surface. Cu, being a representative metal of this class, forms highly stable bonds with the charged intermediate CO_2_˙^−^, enabling the production of very reduced products such as alcohols (*e.g.*, CH_3_OH) and hydrocarbons (*e.g.*, CH_4_). The second class includes metals that create weaker bonds compared to Cu, resulting in the predominant production of CO as the main product. Noble metals such as Pd, Au, and Ag, along with others such as Zn, fall into this category. Lastly, the third class consists of metals where HCOO^−^ is the main product due to the weak binding of the intermediate to the catalyst surface. Metals such as Sn, In, Pb, and Hg are examples of this class.

In conclusion, strategic design and the use of high-entropy alloys (HEAs) with active metals can aid in achieving the desired electrode materials tailored for specific value-added products. Additionally, computational studies and the trial-and-error method can concurrently contribute to the design of HEA-based electrode materials for the CO_2_RR process. Overall, these three classes of catalysts yield higher value-added products such as hydrocarbons or alcohols, CO, and HCOO (or HCOOH depending on the acidity). However, it's essential to consider the competitive process of the hydrogen evolution reaction (HER) in CO_2_ electroreduction in aqueous solutions. In the first class of metals (Cu), where the partially reduced intermediate CO_2_˙^−^ is strongly adsorbed, the HER tends to occur predominantly on metals from the other two classes, such as Pt, Ti, Fe, and Ni. Therefore, fine-tuning the binding energies of key reaction intermediates is crucial for achieving selectivity in desired HEA based products and minimizing the specific energy consumption of CO_2_ electroreduction.

#### Alcohol oxidation reaction

Direct alcohol fuel cells (DAFCs) are a type of proton exchange membrane fuel cell that can directly convert alcohols into electricity at the anode. Common fuels used in DAFCs include methanol, ethanol, propanol, glycerin, and ethylene glycol.^72,74,186^ The production of alcohol can be achieved through various processes, making it a promising renewable energy source for power generation. Researchers have investigated various materials as electrocatalysts for AORs, including noble metals, transition-metal oxides, and carbon-based materials. However, none of these materials have met the ideal criteria for AOR catalysts. Recently, HEAs have emerged as a promising new class of electrocatalysts for alcohol oxidation reactions (AORs). Owing to their tunability, by adjusting the composition of the HEA, the electronic structure, and d-band center of the catalyst can be fine-tuned. This, in turn, modulates the adsorption and reactivity of different intermediates. This tunability allows HEAs to be specifically designed for different AORs, such as the MOR or ethanol oxidation reaction (EOR). HEAs have demonstrated superior CO poisoning resistance as compared to single-component catalysts, effectively dissociating CO molecules, leading to enhanced catalytic activity and durability.^72,74,295^ Moreover, HEAs can be designed with multiple types of active sites, each with a specific function, resulting in more efficient and selective AOR catalysts.

Finally, HEAs can be designed to have multiple types of active sites, each with a specific function. For example, a HEA can be designed to have active sites for alcohol adsorption, C=C bond cleavage, and oxidation of toxic substances. This can result in more efficient and selective AOR catalysts (Table 13). The coordination environment of the metal surface sites can be changed by changing the composition of the HEA alloy. This results in metal sites that balance the adsorption energies of reactants, reactors, and intermediates at the adsorption sites, ultimately achieving high activity.

**Table 13:** HEA-based electrode materials for the alcohol oxidation reaction

Electrode	Synthesis	Electrolyte	Performance and key points	Reference
Pd_40_Ni_60_	Melt spinning technique	1.0 M KOH + 0.5 M methanol	*E*_onset_: −0.83 V, *I*_f_/*I*_b_: 9.3	74
Pd_40_Ni_60_	Melt spinning technique	1.0 M KOH + 0.5 M ethanol	*E*_onset_: −0.81 V	74
Pt_52_Fe_11_Co_10_Ni_11_Cu_10_Ag_8_ NPs	Radio-frequency sputter depositions	0.5 M H_2_SO_4_ + 1.0 M CH_3_OH	Mass activity: 462–504 mA mg^−1^; *E*_onset_ = 0.222 V, *E* at *I*_f_: 0.676, *E* at *I*_b_: 0.518, *I*_f_/*I*_b_: 1.02	186
PtRuCuOsIr Nps	Dealloying	0.5 M H_2_SO_4_ + 0.5 M CH_3_OH	Mass activity: 857.5 mA mg_Pt_^−1^, specific activity: 3.0 mA cm^−2^, *I*_f_/*I*_b_: 1.2	319
Pt_18_Ni_26_Fe_15_Co_14_Cu_27_ NPs	Mechanical method	1 M KOH + 1 M CH_3_OH	Mass activity: 15.04 mA mg_Pt_^−1^	295
PdNiCoCuFe nanotube	Template-assisted electrodeposition	0.5 M CH_3_OH + 0.5 M NaOH	Current density of the forward scan of PdNiCoCuFe alloy NTAs is two-times higher than that of Pd NTAs	319
PtFeCoNiCu (HEA-700)	Calcination and annealing	0.1 M HClO_4_+ 0.1 M CH_3_OH	Specific activity: 3.29 mA cm^−2^ (1.40A mg^−1^), *I*_f_/*I*_b_: 1.21	320
PdPtCuAgAu nanowires	One-pot solution-phase route	1 M KOH+ 1 M ethanol	Mass activity: 7.7 A mg_(Pd+Pt)_^−1^, *I*_f_/*I*_b_: 3.4	322
PdAgSn/PtBi HEA NPs	Wet chemical method	1.0 M KOH + 1 M methanol	Mass activity: 2874 mA mg_(Pd+Pt)_^−1^, 5.9- and 4.8-fold higher than that of commercial Pd/C and Pt/C	323
PdAgSn/PtBi HEA NPs	Wet chemical method	1 M KOH + 1 M ethanol	Mass activity: 3386 mA mg_(Pd+Pt)_^−1^, 5.4- and 4.9-times higher than that of commercial Pd/C and Pt/C	323

**Fig. 22:**
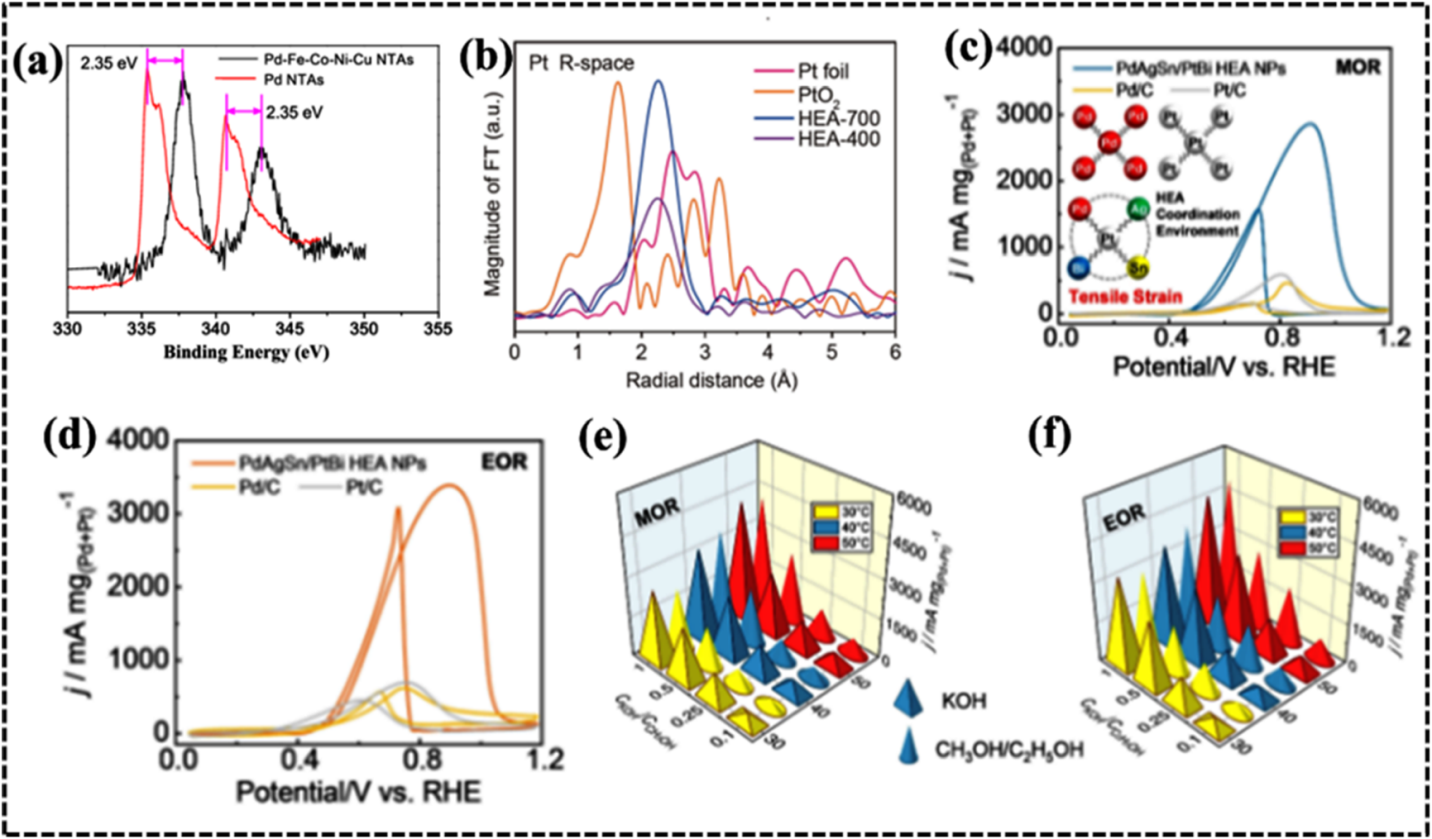
(a) XPS diagram of a PdNiCoCuFe alloy nanotube. Reproduced with permission.^319^ Copyright 2014, Elsevier B.V. (b) Fourier transform of the extended X-ray absorption fine structure spectra of the PtFeCoNiCu HEA for the Pt L3-edge. Reproduced with permission.^244^ Copyright 2021, Spinger Nature. (c and d) Intrinsic methanol oxidation reaction (MOR) and ethanol oxidation reaction (EOR) activities of the designed HEA NPs and (e and f) comparison of the activities of the HEA NPs towards the EOR and MOR. Reproduced with permission.^323^ Copyright 2023, Wiley VCH.

A template-assisted electrodeposition method was employed to fabricate PdNiCoCuFe alloy nanotube arrays (NTAs), exhibiting enhanced methanol oxidation capability achieved by the synergistic interaction between Pd, Ni, Co, Cu, and Fe, which facilitates C–H bond cleavage and hydroxyl species generation (Fig. 22a).^319^ The compositional integrity, geometric arrangement, and strong electronic interaction within the metal provide a synergistic effect and ultimately contribute to the catalytic activity by altering the electronic state of Pd. Therefore, the quinary PdNiCoCuFe alloy NTAs are highly desirable as low-Pd-content catalysts in DAFCs.

The electrochemical AOR process using a HEA-based electrode material involves linear electron and proton transfer processes with critical intermediate orbitals, promoting effective oxidation and bond strength. HEAs provide a suitable electronic environment with multiple surface-active sites, promoting effective electron transfer, facilitating adsorption, and maximizing surface electrical activity, thus facilitating effective conversion.^320^ For example, the MOR activity of the FCC crystalline PtFeCoNiCu HEA was improved by the surface strain strategy.^244^ EXAFS analysis revealed that the shortening of the Pt–Pt bond distance and developed compressive strain significantly improved MOR electrocatalytic performance by downshifting the d-band center (Fig. 22b). Furthermore, DFT calculations indicate that the compressive strain shifts the d-band center away from the Fermi energy, resulting in weaker adsorption of CO on the HEA surface, which improved the MOR performance.

The development of highly efficient direct ethanol fuel cells (DEFCs) faces a challenge in enhancing ethanol C–C bond cleavage through the complete 12-electron EOR. Modern catalysts struggle with incomplete pathways due to CO poisoning, affecting power performance and efficiency. A separated PtPdFeCoNiSnMn HEA (PtPd HEA) was designed to address this issue.^321^ The PtPd HEA exhibited superior activity and stability against the EOR and ORR, respectively, and achieved record performance with a maximum power density of 0.72 W cm^−2^ and stable operation for over 1200 h in DEFCs. Interestingly, Sn was also introduced to promote CO oxidation and C–C bond cleavage; Pt and Pd served as active sites to catalyze the EOR, and the reaction barrier for C–C bond cleavage was significantly reduced at the Pd site of the PtPd HEA because of the synergistic and electronic effects with other elements. Fan *et al.* synthesized PdPtCuAgAu nanowire networks using carboxyl-functionalized surfactants as soft templates. These alloy-based electrocatalysts exhibited enhanced performance in ethanol oxidation, with high mass activity, anti-poisoning ability, superior stability/durability, and fine electrocatalytic kinetics, along with advantages such as anisotropic and thin nanowires.^321,322^

Despite significant advancements in the field of HEAs, controlling their morphology and structure remains a challenge because of the complexities involved in balancing precise HEA surface control with extreme synthesis conditions. Guo *et al.* employed a simple low-temperature synthesis method under atmospheric pressure to fabricate Pd-enriched-HEA-core and Pt-enriched-HEA-shell NPs with a single-phase FCC structure.^323^ Interestingly, both the Pd-enriched-HEA-core and Pt-enriched-HEA-shell NPs exhibited lattice expansion during HEA formation, resulting in tensile strain within the HEA core and shell (Fig. 22c and d). The synthesized PdAgSn/PtBi HEA NPs demonstrated superior electrocatalytic activity and durability for both the MOR and EOR. The specific (mass) activity of PdAgSn/PtBi HEA NPs for the MOR surpassed that of commercial Pd/C and Pt/C catalysts (Fig. 2e and f). Integrating high-entropy elements (HEEs) with Pt and Pd sites at the HEA interface synergistically facilitates a multistep process for the AOR. Consequently, noble metal-based HEA electrodes exhibit enhanced activity compared to their non-noble metal-based counterparts. This is attributed to the structural, electronic, and morphological modifications induced by the addition of noble metals. However, achieving cost-effectiveness through non-noble metal-based HEAs for the AOR remains a challenge, limiting the full potential of this technology in industrial applications.

#### Nitrogen reduction reaction

**Fig. 23:**
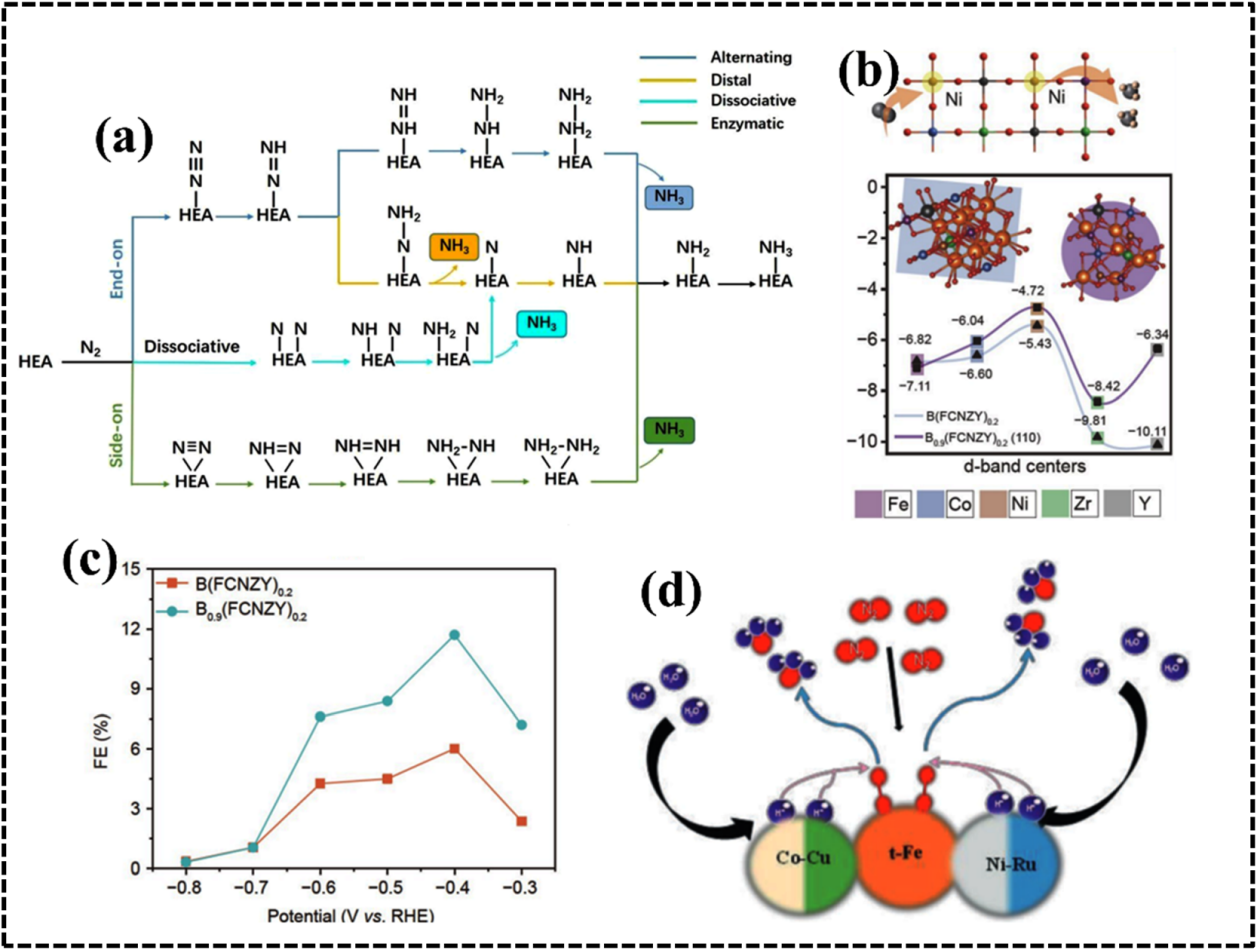
(a) Schematic illustration of different mechanisms for the nitrogen reduction reaction (NRR). Reproduced with permission.^324^ Copyright 2022 Elsevier B.V. (b) d-band centers in the designed high-entropy perovskite oxides. (c) Faradaic efficiency of the designed HEA perovskite toward the NRR, Reproduced with permission.^325^ Copyright 2022 Springer Nature. (d) Mechanism of the HEA for the NRR. Reproduced with permission.^326^ Copyright 2021 Wiley VCH.

The NRR is a sustainable and environment-friendly method for converting N_2_ to NH_3_ under atmospheric conditions. However, the NRR is challenging because of the strong N=N triple bonds in N_2_ and competitive HER. A heterogeneous catalytic pathway was observed, with the adsorption of N_2_ molecules on the electrocatalyst surface, hydrogenation, and desorption of NH_3_ (Fig. 23a).^287^ Understanding the mechanisms of association and dissociation are crucial for designing efficient and selective electrocatalysts for N_2_ to NH_3_ conversion. The activation energy required to break the stable N_2_ triple bond is a major challenge. To improve catalytic capacity, surface control, defect engineering, strain engineering, alloying, composition tuning, and blending strategies are needed. HEAs are also promising for the NRR because of their tunability, which enables the designing of specific NRR catalysts. Proper compositional tuning with early transition metals such as Sc, Y, Ti, and Zr can be a good choice for selective designing of active NRR materials. Yu *et al.* utilized DFT calculations to screen the optimal performance of a FeCoNiCuPd HEA for the NRR. Their findings revealed that the varying metal ratios of HEAs significantly influence the d-band center, consequently affecting the catalytic activity across the HEA surface. Theoretical studies have demonstrated that Ni_0.3_(FeCoCuPd)_0.175_ with an exposed (111) crystal plane exhibits significant NRR activity, achieving an overpotential of 0.34 eV. This assessment was based on the measurements of the adsorption energy of the initial N_2_ and the free energy of intermediates along different pathways. Notably, the bridge site of Fe–Co on the surface emerged as the optimal site for N_2_ adsorption and activation, contributing to the superior NRR catalytic performance. Electronic calculation results indicated that the atomic ratios of HEAs play a crucial role in positioning the d-band center, thereby enhancing the interaction between the metal site and the adsorbate and influencing the catalytic activity. Compared to the secondary HER, the Ni_0.3_(FeCoCuPd)_0.175_ catalyst exhibits a superior selectivity of 99%. This high selectivity is attributed to the stronger adsorption of H, which favors the NRR over the HER. Regardless of the element ratio in HEAs, the PDOS results consistently reveal that optimal N2 adsorption configurations involve Fe or Co atoms. These atoms facilitate electron transfer to N2 molecules during their reduction to NH_3_ (Table 14).^324^

**Table 14:** HEA-based electrode materials for the nitrogen reduction reaction

Electrode	Synthesis	Electrolyte	Performance and key points	Reference
B-doped FeCoNiCuPd	Electrospinning	0.5 M K_2_SO_4_	NH_3_ yield of 24.8 μmol h^−1^ cm^−2^, NH_3_ FE of 39.2%	287
Ni_0.3_(FeCoCuPd)_0.175_	—	—	Ni increases NRR activity	324
RuFeCoNiCuNps	Wet chemical	1 M KOH	NH_3_ yield of 11.4 μg h^−1^ cm^−2^, NH_3_ FE of 39.2%	325

Chu *et al.* synthesized high-entropy perovskite oxides, Ba_*x*_(FeCoNiZrY)_0.2_O_3−*δ*_ (B_*x*_(FCNZY)_0.2_) (*x* = 0.9, 1) (where FCNZY represents Fe, Co, Ni, Zr, and Y, respectively) using a modified sol–gel method.^325^ By modifying the non-stoichiometric metal elements at the A site and introducing oxygen vacancies, the NRR activity of the high-entropy perovskite oxide was significantly enhanced. Employing the d-band center theory, the catalytically active center at the B site was theoretically predicted, identifying Ni as the primary catalytic site. Moreover, Ni, Co, and Fe also served as catalytically active sites in the bulk B(FCNZY)_0.2_, as their energy levels approach the Fermi level, as confirmed *via* the d-band center analysis (Fig. 23b). The free energy values of the intermediate states in the optimal distal pathway reveal that the third protonation step (*NNH_2_ → *NNH_3_) is the rate-determining step, and the increased oxygen vacancies in the high-entropy perovskite facilitate nitrogen adsorption and reduction. The enhanced NRR activity of B_0.9_(FCNZY)_0.2_ is attributed to the additional oxygen vacancies and the synergistic effect between different metal elements at the B site, as corroborated by DFT calculations (Fig. 23c).

High-voltage requirements, low-NH_3_ yield, and poor stability of electrode materials pose significant challenges for the NRR. To address these issues, RuFeCoNiCu HEA NPs were synthesized in an oil phase at atmospheric pressure and low temperature (≤250 °C), demonstrating NRR activity in 0.1 M KOH, 0.1 M Li_2_SO_4_, 0.1 M Na_2_SO_4_, and 0.1 M HCl electrolytes.^326^ The designed HEA NPs exhibited a high NH_3_ yield of 57.1 μg h^−1^ mg_cat_^−1^ at 0.05 V *versus* the reversible hydrogen electrode in 0.1 M KOH, with a corresponding faradaic efficiency of 38.5%. Monte Carlo and DFT calculations suggested that Fe in the alloy is the most favorable site for N_2_ adsorption and activation, whereas Co–Cu and Ni–Ru pairs exhibit excellent surface hydrogenation ability at low overpotential, forming H* on their surfaces. Specifically, t-Fe, b-Fe-Fe, h-Co-Ni-Ni, and s-Ni–Ru are identified as four promising active sites, and t-Fe was identified to be the most effective for the NRR (Fig. 23d). This investigation introduces a novel method for HEA synthesis and demonstrates the application of HEAs in the NRR under various pH conditions. These findings shed light on a new NRR mechanism with potential for commercial applications. While the strategic design of HEA-based electrode materials is still in its early stages, significant progress can be made towards achieving commercially viable performance through selective metal choices, design, and optimization. Additionally, computational studies offer a valuable tool to bridge the gap between current experimental limitations and the efficient evaluation of potential HEAs.

#### Energy storage

**Fig. 24:**
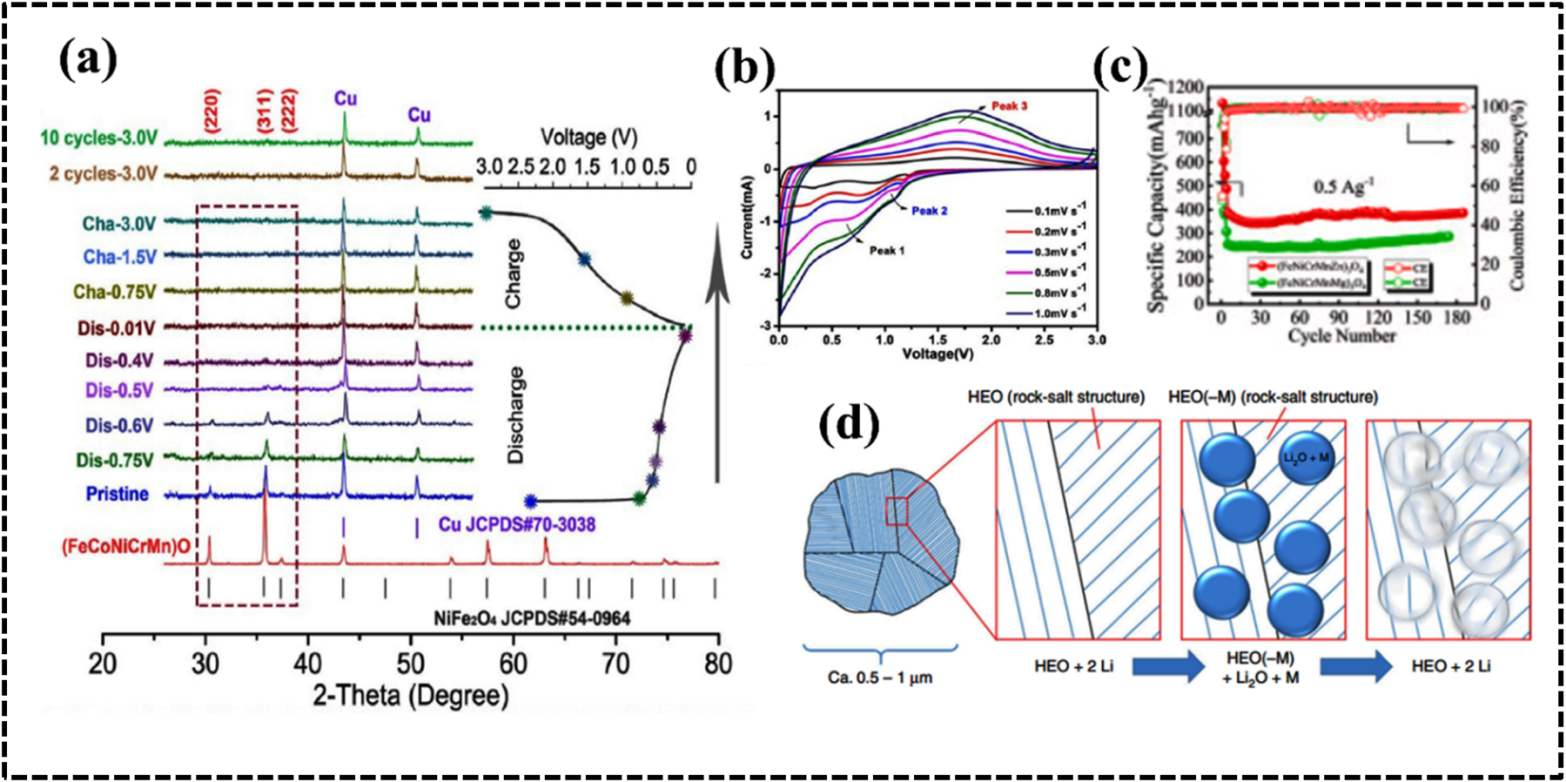
(a) *Ex situ* XRD patterns of high-entropy oxide (HEO) at different states of charge. (b) Kinetics analysis of HEO in Li-ion batteries. Reproduced with permission.^129^ Copyright 2022, Elsevier B.V. (c) Electrochemical performance of HEAs as an anode material. Reproduced with permission.^327^ Copyright 2021, Elsevier Ltd. (d) Schematics of the proposed de-/lithiation mechanism during the conversion reaction of transition-metal-based HEO. M in the figure denotes only the cations in the high-entropy system. Reproduced with permission.^329^ Copyright 2018, Nature Spinger.

Electrochemical energy storage (EES) involves converting electrical energy into chemical energy during charging, and then reversing the process to generate electrical energy again during discharging. EES devices are crucial for integrating renewable energy, powering electric vehicles, and providing backup power. This rapidly evolving field boasts numerous emerging technologies poised to play an increasingly significant role in our energy future. EES devices can be broadly categorized based on their charge storage mechanism: batteries utilize redox-active materials, while supercapacitors rely on electrical double-layer capacitor-type materials.^326^ Depending on the electrochemical nature of HEAs, they can be employed in multiple energy storage processes, including supercapacitors and batteries (Table 15). Qi *et al.* designed a novel spinel-structured HEO, (FeCoNiCrMn)_3_O_4_, which was prepared *via* a high-temperature solid-state reaction and evaluated as an anode for LIBs. *In situ* high-temperature XRD was used to reveal the structure evolution of mixed oxides with the increase in calcination temperature, and a single-phase spinel-structured (FeCoNiCrMn)_3_O_4_ was obtained at 900 °C (HEO-900; Fig. 24a).^129^ The effect of temperature on the structure and electrochemical performance of HEO was investigated, and the HEO-900 anode with commercial mass loading was observed to exhibit higher capacity (discharge/charge, 1034/680 mA h g^−1^) and better rate capability (182 mA h g^−1^ at 2 A g^−1^) than the structures obtained at 950 and 1000 °C (HEO-950 and HEO-1000, respectively) for its moderate particle size; moreover, all three samples showed superior cycling stability.

**Table 15:** HEA-based electrode materials for energy storage processes

Electrode	Synthesis	Electrolyte	Performance & key points	Reference
(FeCoNiCrMn)_3_O_4_	Calcination	1 M LiPF_6_ + ethylene carbonate (EC)/ethyl methyl carbonate (EMC)/dimethyl carbonate (DEC) (1 : 1 : 1)	Coulombic efficiency: 61.6%, discharge/charge specific capacities: 1034 mA h g^−1^/680 mA h g^−1^	129
(FeNiCrMnZn)_3_O_4_	Ball milling	1 M LiPF_6_ + ethylene carbonate/dimethyl carbonate (1 : 1)	Discharge capacity (386.7 mA h g^−1^) at 0.5 A g^−1^ after 185 cycles	327
(Co_0.2_Cu_0.2_Mg_0.2_Ni_0.2_Zn_0.2_)O	Spray pyrolysis	—	Coulombic efficiency: 99.4–99.95%	329
Li_1.3_Mn_0.1_Co_0.1_Mn_0.1_Cr_0.1_Ti_0.1_Nb_0.2_O_1.7_F_0.3_	Solid state method	1 M LiPF_6_ + ethylene carbonate/dimethyl carbonate (1 : 1)	Specific energy: 307 mA h g^−1^ (955 W h kg^−1^)	330
Fe_0.24_Co_0.26_Ni_0.10_Cu_0.15_Mn_0.25_		Lithium nitrate (LiNO_3_, 0.2 M) lithium bis(trifluoromethanesulfonyl)imide (LiTFSI, 1 M) in DME/DOL (1 : 1)	Specific capacity: 1079.5 mA h g_cathode_^−1^	331
(FeCoNiCrMn)_3_O_4_	Arc melting and dealloying	2 M KOH	Specific capacitance of 639 F g^−1^ at 1 A g^−1^ and retention of 80.77% at 10 A g^−1^	332
(FeCoCrMnNi)_3_O_4_	Sol–gel method	1 M KOH	Capacitance: 332.2 F g^−1^ at 0.3 A g^−1^, energy density: 103.8 W h kg^−1^, potential window: −1 to 0.6	333
Fe–Co–Ni–Cu–Zn	Ball milling	3 M KOH	Gravimetric capacitance of 325.17 F g^−1^ at 1 A g^−1^, energy density: 3.82 W h kg^−1^, power density: 325 W kg^−1^	334

The superior electrochemical performance of (FeCoNiCrMn)_3_O_4_ can be ascribed to the optimized electron configurations, smooth ion diffusion paths and abundant active storage sites derived from the unique structure of HEO with multiple metal elements of mixed valence states and different cation radii. The cathodic peak located at 0.25 V is attributed to the stepwise reduction of transition-metal oxides (containing M^4+^/M^3+^ toM^2+^ and further M^2+^ to M^0^) and the formation of an inert solid electrolyte interface layer, indicating HEA-based redox behavior and a battery-type electrode material (Fig. 24b).^327^ This HEO amorphous structure is good for achieving an anode with superior electrochemical performance. The defects in amorphous HEO not only provide more possible active sites for Li^+^ storage, but also open sufficient diffusion paths for Li^+^ through the electrode and regulate electron configurations to accelerate the transport of electrons, giving rise to increased capacity. Furthermore, the disordered structure provides more space for buffer volume changes during the repeated lithiation and delithiation. In another report, Dai *et al.* confirmed that Zn improved the content of trivalent Mn in HEOs, thereby increasing the voltage range of the reaction process, which then enhances the Li storage performance. The comparison between (FeNiCrMnZn)_3_O_4_ and (FeNiCrMnMg)_3_O_4_ suggested that, when compared to Mg, the introduction of Zn improved the diffraction peak intensity and crystallinity of the XRD spectra, which was then reflected in their Li storage performance (Fig. 24c).^327^ Apart from the anodic electrode performance, HEO has received considerable attention as the most promising cathode material for next-generation LIBs.^328^

**Fig. 25:**
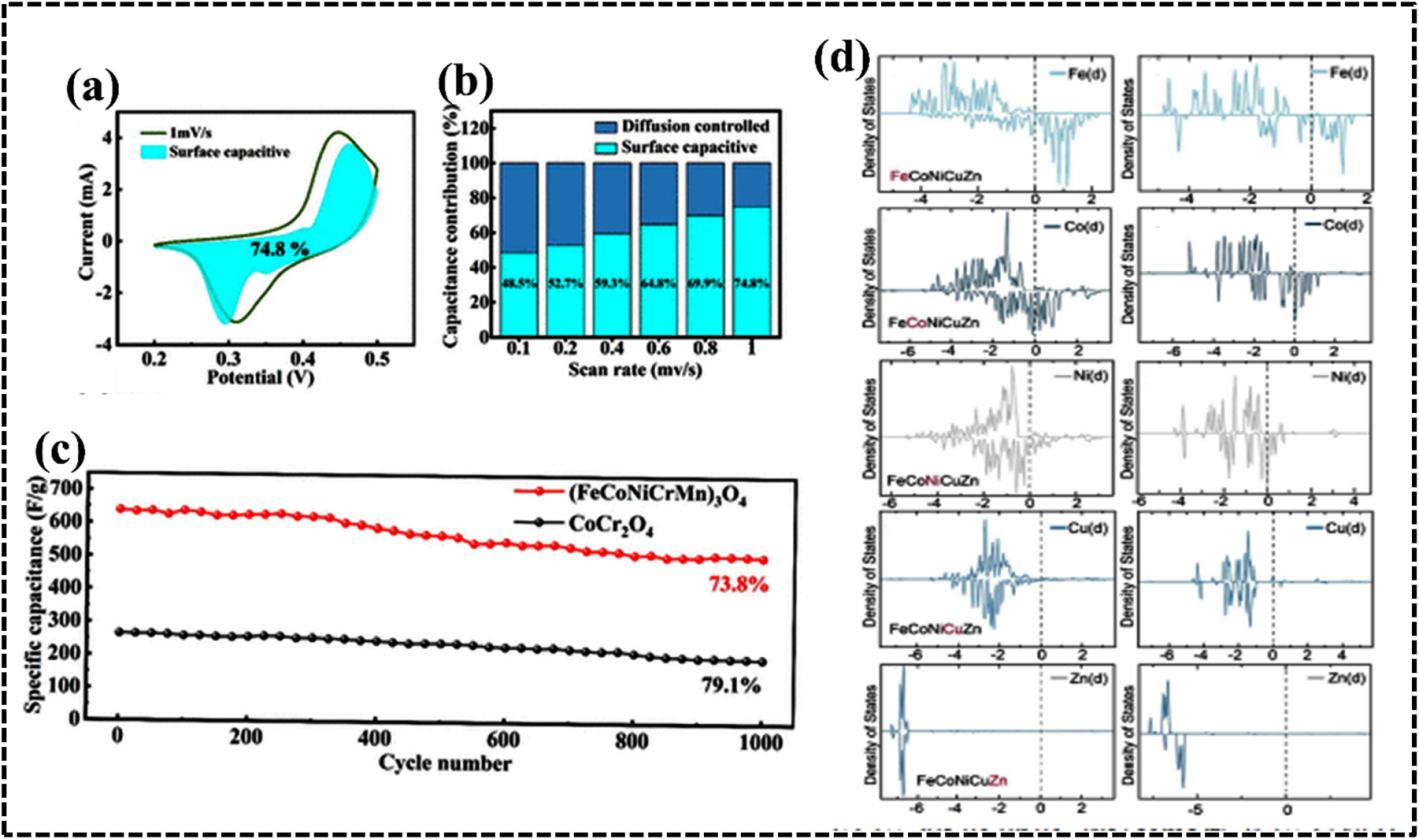
(a–c) Capacitive contribution, separations of diffusion-controlled and capacitive charges at different scan rates, and cycling performances of (FeCoNiCrMn)_3_O_4_/NF and CoCr_2_O_4_/NF. Reproduced with permission.^332^ Copyright 2023, American Chemical Society. (d) Plots of density of states for d-states of Fe, Co, Ni, Cu, and Zn in the FeCoNiCuZn HEA, which is actively used as electrode materials in supercapacitor devices. Reproduced with permission.^334^ Copyright 2023, Elsevier Ltd.

Sarkar *et al.* demonstrated the reversible lithium storage properties of HEOs, underlying mechanism, and influence of entropy stabilization on the electrochemical behavior. It was found that the stabilization effect of entropy brings significant benefits for the storage capacity retention of HEOs and significantly improves the cycling stability of the proposed lithiation or delithiation mechanism during the conversion reaction of (Co_0.2_Cu_0.2_Mg_0.2_Ni_0.2_Zn_0.2_)O HEO. The as-prepared transition-metal-based HEO (TM-HEO) was composed of poly-/nanocrystallites, exhibiting an ordered structure; moreover, during lithiation, some of the divalent metals of TM-HEO reacted with Li to form nano-Li_2_O and nano-M nuclei *via* a conversion reaction (Fig. 24d).^329^ The rock-salt structure was preserved in this state and nanosized nuclei grew inside the rock-salt host structure, causing the buildup of stresses, thus resulting in the introduction of defects. Consequently, the participating ions remained trapped inside the host matrix and could easily diffuse back into the crystal structure in the subsequent oxidation process. Hence, the parent HEO structure was restored after the delithiation process. Therefore, the charge–discharge process is reversible and rapid in the rock-salt-like HEO system. Lun *et al.* demonstrated a cation-disordered rock-salt-type HEO for LIBs where cathode compound, composed of six different transition-metal species, was approximately 40% larger than the capacity of the conventional compound. The incorporation of a large number of TM species into the lattice could reduce the extent of short-range order and significantly improve Li transport.^330^ Gao *et al.* developed a novel sulfur cathode comprising Fe_0.24_Co_0.26_Ni_0.10_Cu_0.15_Mn_0.25_ HEA nanocrystallites embedded on nitrogen-doped carbon.^331^ This unique architecture enabled the cathode to exhibit remarkable electrocatalytic activity in converting solid sulfur into solid discharged products, bypassing the soluble intermediate lithium polysulfide phase. Consequently, the cathode achieved a superior reversible capacity of 1079.5 mA h g_cathode_^−1^, corresponding to a high utilization of 89.4%, with the entire cathode serving as the active material instead of solely the sulfur element. Notably, even under challenging conditions involving a lean electrolyte (3 μL mg^−1^) and ultrahigh sulfur loading (27.0 mg cm^−2^), the cathode retained an impressive discharge capacity of 868.2 mA h g_cathode_^−1^. This groundbreaking approach paves the way for exploring catalytic host materials that can effectively enhance sulfur utilization in lithium–sulfur batteries. Sui *et al.* synthesized (FeCoNiCrMn)_3_O_4_ by dealloying the FeCoNiCrMnAl_95_ alloy in 3 M NaOH, achieving superior supercapacitor characteristics as an anode material, with a specific capacitance of 639 F g^−1^ at 1 A g^−1^ and retention of 80.77% at 10 A g^−1^.^332^ The reduction peak occurs at 0.3 V, which is predominantly caused by the transition-metal oxide reduction (M^4+^/M^3+^ to M^2+^, M^2+^ to M^0^), indicating that the materials rely on noticeable redox reactions for energy storage. The better capacitive performance can be compared with that of CoCr_2_O_4_ (Fig. 25a and b). Ma *et al.* fabricated spinel-phase (FeCoCrMnNi)_3_O_4_ at a low calcination temperature of 450 °C, achieving a high specific surface area of 63.1 m^2^ g^−1^ in an alkaline medium. The specific capacitance was observed to be 332.2 F g^−1^ at a current density of 0.3 A g^−1^ in 1 M KOH electrolyte with a wide potential window of (−1, 0.6). The HEO electrode achieved an energy density of 103.8 W h kg^−1^ by maintaining the designed microstructural arrangement.^333^

Tiwary *et al.* successfully synthesized a Fe–Co–Ni–Cu–Zn HEA using a ball milling method.^334^ This HEA exhibited multivalent quinary characteristics and favorable intermediate intercalation/deintercalation kinetics, making it a promising candidate for supercapacitor applications. The asymmetric liquid device performance was attributed to the presence of multivalent elements with surface oxidation and the availability of d-states near the Fermi level. The device demonstrated stable performance over long-term (up to 5000 cycles) charging–discharging cycles in a voltage window of 1.5 V. It achieved a maximum specific capacitance of approximately 105 F g^−1^ at a scan rate of 5 mV s^−1^. The fabricated liquid-state device exhibited a superior specific energy of 23.82 W h kg^−1^ and specific power of 375 W kg^−1^. The enhanced electronic conductivity and electron transport properties of the HEA alloy contributed to the reduction and formation of split energy levels, as confirmed *via* DFT calculations. The d-band center of the quinary Fe–Co–Ni–Cu–Zn HEA was significantly closer to the Fermi level as compared to that of individual atom nanoclusters, resulting in increased chemical affinity toward oxygen absorbability on the surface of the mesoporous HEA (Fig. 25c). This tailored surface chemistry led to improved chemical stability, enhanced reaction kinetics, and reduced ion diffusion barriers.

Developing and optimizing industrial electrocatalysts pose a significant challenge for the scientific community. Industrial electrode materials must adhere to a set of fundamental requirements that are not typically considered for laboratory-scale or research-based development and synthesis. Generally, electrode materials for industrial applications must meet specific criteria, including (a) a straightforward synthesis approach, (b) a product-efficient synthesis route, (c) environmentally friendly technology, (d) cost effectiveness, and (e) high catalytic efficiency, stability, and selectivity. The feasibility of employing HEAs as industrial electrode materials compared to conventional electrode materials hinges on systematically addressing these criteria. The introduction of HEAs and their derivatives has revolutionized the field by introducing an entirely new class of materials, finding applications in a range of electrochemical processes. HEAs, therefore, stand as promising candidates for revolutionizing catalytic research and, with the right design strategy and understanding of structure–property relationships, can potentially transition into industrial applications. The intrinsic properties of HEAs result in unique characteristics such as high mechanical strength, superior fracture toughness, and corrosion resistance. Utilizing these HEAs in catalytic reactions is a relatively new area of research.

## Summary and outlook

HEAs demonstrate immense potential for designing new, cost-effective catalysts by utilizing low-cost transition metals instead of precious noble metals (Table 16). Precise control over both the electronic and morphological structure of HEAs, including factors like composition, phase distribution, and surface characteristics, is crucial for optimizing their performance in specific electrocatalytic applications. Alongside exploring the appropriate compositional possibilities, a major challenge in fabricating HEAs lies in achieving precise size control, selecting compositions accurately, and controlling morphology. This challenge can be overcome through systematic studies of their crystal structure, phases, electronic arrangement, and stability under various operating conditions. Undoubtedly, the kinetic and thermodynamic parameters of a HEA play significant roles in determining its phase stability, crystal structure, and electronic arrangement, and identifying the most promising composition. Therefore, before designing a HEA system for an electrochemical application, all these parameters should be considered to achieve optimal performance. The systematic design and specific application of HEAs must be addressed through a proper designing approach that considers the theoretical knowledge of the selected elements with dissimilar periodic properties. The catalytic properties of HEAs are complex and multifaceted owing to the involvement of various elements. Although this multiplicity can lead to poor selectivity of products, it also holds the potential for new and unexpected molecular transformations arising from atomic arrangements on the surface of HEAs. The selection of periodic elements is an important consideration before designing HEA-based potential electrode materials for a particular electrochemical process. This is facilitated by the use of advanced computational and experimental techniques.

**Table 16:** Comparison of HEA-based electrode materials with traditional materials as electrode materials

Electrochemical process	Traditional materials	Property/aspect of high-entropy materials (HEMs)	Advantages of HEMs	Challenges of HEMs
HER	Pt, Ni, Mo, MoS_2_, and NiFe	(a) Synergistic effects and a tunable electronic structure and (b) high stability under harsh conditions	(a) Improved activity and durability compared to pure metals. (b) Can be tuned for specific pH conditions	(a) Synthesis complexity and cost. (b) Limited understanding of structure–property relationships
OER	Ir, Ru, Co, RuO_2_, IrO_2_, and LaMnO_3_	(a) Improvement through compositional tuning and (b) high stability, especially in an acidic medium	(a) Enhanced activity and lower overpotential compared to traditional oxides. (b) Potential for cost reduction compared to noble metals	(a) Difficulty in controlling surface composition during synthesis. (b) Long-term stability concerns
ORR	Pt, Ag, and Pt alloy	(a) Comparable or superior activity. (b) High stability with potential for improvement through alloying	(a) Exhibit comparable activity to Pt at significantly lower cost. (b) Potential for improved durability compared to Pt	(a) Achieving high selectivity towards O_2_ reduction (avoiding H_2_O production). (b) Difficulty in fine-tuning activity and selectivity
CO_2_RR	Cu, Sn, Ag, and Cu alloy	(a) Competitive activity through synergistic effects, (b) tunable selectivity towards the desired product, and (c) high stability, especially in aqueous solutions	(a) Tunable product selectivity (CO, HCOOH, CH_4_, *etc.*) based on the composition. (b) Potential for enhanced activity and improved conversion efficiency	(a) Identifying optimal compositions for the desired products. (b) Difficulty in controlling morphology and active sites
NRR	Fe, Mo, and MoS_2_	(a) Tunable electronic structure and (b) tunable selectivity	(a) Achieve high ammonia selectivity and activity at lower overpotentials. (b) Potential for cost-effective alternatives to noble metal catalysts	(a) Identifying optimal compositions for N_2_ activation and H_2_ dissociation. (b) Long-term stability issues for some HEMs
AOR	Pt and Pd	(a) Stability for compositional complexity and (b) the initial cost may be higher but potential for cost reduction with optimization	(a) Potential for lower cost and improved CO poisoning tolerance compared to Pt. (b) Tunable activity and selectivity for specific alcohols	(a) Difficulty in achieving high activity and selectivity simultaneously. (b) Long-term stability needs further investigation
Battery	LiCoO_2_, LiFePO_4_, and carbonaceous material	(a) Potential for high capacity and long cycle life and (b) cost-effectiveness may vary depending on the specific application	(a) High energy density and tunable capacity based on the composition. (b) Potential for improved cycling stability and safety	(a) Understanding complex phase transformations during operation. (b) Optimizing synthesis methods for achieving the desired morphologies
Supercapacitor	Carbonaceous materials and MnO_2_	(a) Potential for high capacitance and fast charge–discharge rates and (b) high stability under cycling conditions	(a) High capacitance and potential for fast charge/discharge rates. (b) Tailoring the pore structure and surface area for enhanced performance	(a) Identifying cost-effective and scalable synthesis methods. (b) Exploring strategies for improving long-term cycling stability

Despite the promising potential of HEA-based electrode materials, several challenges need to be addressed before they can be widely adopted in industrial processes. Fabricating HEAs for industrial applications requires the utilization of suitable synthesis approaches that adhere to moderate reaction conditions that are compatible with industrial requirements. Alongside reaction conditions, product percentage and purity are also crucial design factors for selecting a specific synthesis route that enables controlled composition, size, and uniformity. The catalytic performance of the HEA system is significantly influenced by factors such as crystal structure, phase, morphology, counter anions, and exposed active planes. Various designing techniques and engineering approaches (defect, strain, bandgap, d-band, active crystal plane, *etc.*) are available to control these parameters and facilitate the development of electrode materials that are suitable for industrial processes. During synthesis, the judicious adjustment of reaction conditions, including metal precursors, growth control agents, reducing agents, solvents, surfactants, masking agents, and others, offers flexibility for tailoring the structure and morphology of HEAs. Notably, although numerous studies have explored the impact of the phase structure on the mechanical, magnetic, and corrosion properties of HEAs, research on how the phase structures of HEAs with identical compositions affect their electrocatalytic behavior remains limited. Therefore, further investigation into this area is of paramount importance for future research.

The structure–activity relationship is a crucial parameter in designing more effective electrode materials. In this context, a thorough understanding and in-depth study of individual HEAs are essential for elucidating the mechanism and identifying active intermediates. Advanced physicochemical studies can provide valuable insights into these aspects and inform the design and development of potential electrode materials for specific electrochemical processes. Although the multi-element nature of HEAs can introduce complexity, this can be mitigated through *in situ* studies during electrochemical and HEA design processes. *In situ* studies enable the monitoring and tuning of catalytically active sites, growth processes, morphological changes, composition, chemical valence states, and electronic structures of elements in HEAs. Electrochemical operando studies are expected to be particularly useful in measuring structural evolution and identifying the actual active sites of catalysts. Therefore, developing a more comprehensive and sophisticated set of *in situ* techniques is crucial for achieving a profound understanding of HEA-based catalysts during electrocatalytic processes that help to tune optimized electrode materials for industrial processes such as PEMWEs, PEMFCs, energy storage devices, *etc.*

As HEAs are composed of multiple elements with various compositional possibilities, thereby the structure and activity can alter significantly with compositional and elemental variations of the constituent elements. The most efficient compositional and elemental combination can be effectively assessed using theoretical and computational modelling. Moreover, the desired electrochemical performance may not always match the expected results. Therefore, a comprehensive theoretical study is crucial for designing and developing efficient electrode materials for industrial applications. The involvement of novel computational methods, such as DFT, molecular dynamics, ML, NNs, and HT screening, has facilitated the evaluation of a larger number of active sites and accelerated the understanding of the potential for adjusting adsorption energies for a specific electrochemical process. The identity of the active intermediate in the designed electrode materials can be predicted using theoretical calculations. These advanced techniques have aided in the design of potential electrode materials with improved catalytic performance. However, the theoretical understanding may not always align perfectly with experimental findings. While these models can provide valuable guidance, their predictions must be interpreted with caution and validated experimentally. Therefore, a collaborative approach seamlessly integrating theoretical calculations with experimental verification is crucial for advancing HEAs as efficient electrocatalysts and realizing tangible industrial benefits.

**Fig. 26:**
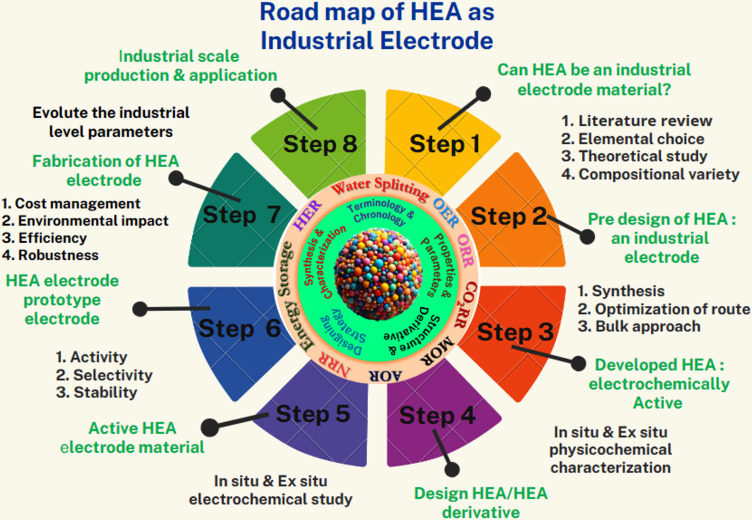
Road map of HEA based electrodes to use as industrial electrode materials.

The vast composition space and unique high-entropy structure of HEAs offer promising opportunities for catalyzing various reactions. However, further research efforts are required to fully unlock their potential. HEA-based heterogeneous catalysts offer four core effects that provide distinct advantages compared to mono- or bimetallic electrode materials or electrocatalysts. Numerous electrochemical processes utilize HEA systems that exhibit superior electrochemical performance when compared to other state-of-the-art materials. However, there remains significant scope for designing new HEA-based electrode materials with low cost and robust performance. Prior to utilizing any HEA-based electrode material in an industrial process, its activity, selectivity, and stability must be carefully evaluated. The selection of appropriate elements and the maintenance of compositional integrity are crucial factors in designing efficient electrode materials. The applicability of the HEA system is determined by the suitable choice and optimization of these parameters. In addition to pristine HEAs, their derivatives also hold immense potential for various electrochemical processes. In some cases, these derivatives have demonstrated superior electrochemical activity when compared to pristine HEAs. Therefore, through proper tuning and optimization, HEA-based materials can be designed to serve as highly effective electrode materials in industrial electrochemical processes. Likewise, the formation of pre-catalysts can be an effective approach to enhance electrochemical performance and achieve optimal results. Furthermore, there remains a gap between industry and academia. Extensive research and development efforts are needed to realize the goal of utilizing HEAs in industrial electrochemical processes. The following approach can offer a pathway to achieve this goal (Fig. 26).

One of the main challenges is the design of HEAs for electrochemical processes. In addition, it is important to develop a cost-effective and scalable manufacturing process while maintaining the desired composition and properties of the HEA. To address this issue, a thorough literature review of electrochemical processes should be conducted. After understanding the process mechanisms using specific benchmark materials, potential HEA materials can be identified. In addition, modern computational science would also be helpful in supporting and assisting in the design of the most efficient electrode materials. The most compositionally active materials can be identified through thorough theoretical studies and optimization of synthetic pathways using trial-and-error methods. It is accurate to conclude that computational studies alone cannot be used to choose the most suitable HEA for industrial electrochemical applications. Tuning the composition and structure of HEAs to maximize their performance in a particular electrochemical process is another challenge. This requires a deep understanding of the relationship between composition, microstructure, and electrochemical properties. Ensuring the long-term stability and durability of HEAs under realistic operating conditions is essential for their industrial adoption; HEAs must withstand the harsh environments and cyclic stresses encountered in electrochemical processes without significant degradation.

Therefore, all the designed and optimized HEAs should be tested under real operating conditions in the device and prototype levels. In order to calculate the efficiency of the designed HEA, appropriate stability tests should be performed on the HEA system. Moreover, physicochemical study helps to evaluate the proper mechanistic routes to find out the active intermediate of the materials. An improved electrode performance can be achieved by the modifying the electronic structure, morphology, crystal face, lattice structure, the composition, *etc.* In addition, HEAs need to be compatible with existing industrial processes and technologies, including electrode fabrication methods and device integration. It is crucial that HEAs can be seamlessly integrated into existing systems without compromising performance; HEAs offer promising properties, but their commercial feasibility depends on their cost-effectiveness when compared to conventional materials. Optimizing manufacturing processes to reduce production costs and improve material efficiency is essential to transfer the technology from the lab to the industry level. Addressing these challenges will require interdisciplinary efforts involving materials scientists, electrochemists, engineers, and industry partners. Collaboration between academia and industry can facilitate the translation of fundamental research findings into practical applications. Moreover, continued investment in research and development, along with advancements in manufacturing and characterization techniques, will be essential for unlocking the full potential of HEAs as industrial electrode materials across a wide range of electrochemical applications.

This study intends to offer a comprehensive overview of the strengths and limitations of HEA-based electrode materials in electrochemical applications. Through critical evaluation of current state-of-the-art research, the study aims to guide future research directions and contribute to the development of more effective HEA-based electrode materials for industrial purposes. With ongoing advancements, HEA-based electrode materials are anticipated to showcase significant potential for diverse industrial applications in the near future, thereby generating economic and environmental benefits.

## Author contributions

Saikat Bolar: conceptualization, writing – original draft, and writing – review & editing. Yoshikazu Ito: visualization and writing – review & editing. Takeshi Fujita: conceptualization, writing – review & editing, and funding acquisition.

## Conflicts of interest

There are no conflicts to declare.

## Supplementary Material
